# HVSeeker: a deep-learning-based method for identification of host and viral DNA sequences

**DOI:** 10.1093/gigascience/giaf037

**Published:** 2025-05-15

**Authors:** Abdullatif Al-Najim, Sven Hauns, Van Dinh Tran, Rolf Backofen, Omer S Alkhnbashi

**Affiliations:** Information and Computer Science Department, King Fahd University of Petroleum and Minerals, Dhahran 34462, Saudi Arabia; Bioinformatics group,Department of Computer Science, University of Freiburg, Georges-Köhler-Allee 101, 79110 Freiburg, Germany; Information and Computer Science Department, King Fahd University of Petroleum and Minerals, Dhahran 34462, Saudi Arabia; Bioinformatics group,Department of Computer Science, University of Freiburg, Georges-Köhler-Allee 101, 79110 Freiburg, Germany; Signalling Research Centres BIOSS and CIBSS, University of Freiburg, Schänzlestr. 18, 79104 Freiburg, Germany; Center for Applied and Translational Genomics (CATG), Mohammed Bin Rashid University of Medicine and Health Sciences(MBRU), Dubai Healthcare City, 505055 Dubai, United Arab Emirates; College of Medicine, Mohammed Bin Rashid University of Medicine and Health Sciences (MBRU), Dubai Healthcare City, 505055 Dubai, United Arab Emirates

**Keywords:** genomics, bacteria, phages, metagenomic, deep learning

## Abstract

**Background:**

Bacteriophages are among the most abundant organisms on Earth, significantly impacting ecosystems and human society. The identification of viral sequences, especially novel ones, from mixed metagenomes is a critical first step in analyzing the viral components of host samples. This plays a key role in many downstream tasks. However, this is a challenging task due to their rapid evolution rate. The identification process typically involves two steps: distinguishing viral sequences from the host and identifying if they come from novel viral genomes. Traditional metagenomic techniques that rely on sequence similarity with known entities often fall short, especially when dealing with short or novel genomes. Meanwhile, deep learning has demonstrated its efficacy across various domains, including the bioinformatics field.

**Results:**

We have developed HVSeeker—a host/virus seeker method—based on deep learning to distinguish between bacterial and phage sequences. HVSeeker consists of two separate models: one analyzing DNA sequences and the other focusing on proteins. In addition to the robust architecture of HVSeeker, three distinct preprocessing methods were introduced to enhance the learning process: padding, contigs assembly, and sliding window. This method has shown promising results on sequences with various lengths, ranging from 200 to 1,500 base pairs. Tested on both NCBI and IMGVR databases, HVSeeker outperformed several methods from the literature such as Seeker, Rnn-VirSeeker, DeepVirFinder, and PPR-Meta. Moreover, when compared with other methods on benchmark datasets, HVSeeker has shown better performance, establishing its effectiveness in identifying unknown phage genomes.

**Conclusions:**

These results demonstrate the exceptional structure of HVSeeker, which encompasses both the preprocessing methods and the model design. The advancements provided by HVSeeker are significant for identifying viral genomes and developing new therapeutic approaches, such as phage therapy. Therefore, HVSeeker serves as an essential tool in prokaryotic and phage taxonomy, offering a crucial first step toward analyzing the host–viral component of samples by identifying the host and viral sequences in mixed metagenomes.

**Key points:**

- We introduce HVSeeker, a novel deep-learning method for classification of bacteria and phage genomes.
- We create three different strategies for creating genomic input sequences and benchmark their effectiveness.
- Data preprocessing with padding achieved better results than using contigs assembly or a sliding window
- HVSeeker compares favorably to alternative classification methods, e.g., Seeker, Rnn-VirSeeker,DeepVirFinder, and PPR-Meta, even on low-homology datasets.
- Additionally we finetune a small ProtBert-based model to provide an additional mechanism to evaluate genomic sequences.

## Introduction

Viruses, the most common organisms on the planet [1], significantly affect both ecosystems and human health [2]. Capable of infecting a wide range of species, including humans and bacteria, viruses exert a profound effect on bacteria populations. Phages, which specifically target bacteria, have a meaningful impact on their host and also influence human health. This underscores the importance for humans of the interaction between bacteria and phages [3]. Phages infect bacteria by first injecting their viral DNA into the bacteria, after breaking down the cell wall with endolysins. In the next step, the phage DNA either integrates into the bacterial DNA or initiates a lytic cycle, using the bacterial replication instruments to reproduce its DNA [4]. Ultimately, the viral genome and proteins assemble to form a new virion [4].

Due to the constant competition between phages and bacteria, the former have developed defense mechanisms that can be used as an alternative to antibiotics [5]. Furthermore, due to their ability to lyse bacterial cell walls, phage-derived endolysins have been suggested as an antimicrobial agent [6], making the phage–bacteria relationship a hot research topic [6]. Because phages insert their DNA into the bacterial host as part of their replication process, it is crucial to distinguish the phage-derived sequences from the bacterial sequences in any genome found in nature. The detection of phage within the host genome may have a significant impact on studying and understanding such viruses; however, the task is time consuming and requires extensive laboratory work [7]. One possible approach to overcome such problems is to analyze metagenomic data that embed virus information as this is shorter than the full genome, ranging from 600 to 25,000 base pairs (bp) [8], which requires less time and effort. Metagenomics is the study of metagenomes from various environmental samples [8]. It is divided into two main areas: structural and functional metagenomics. Structural metagenomics primarily focuses on gene structure, whereas functional metagenomics examines the functions of genes, specifically the proteins they encode [9]. To classify the origin of a genome found in the environment, sequencing methods can be used.

Earlier sequencing methods were based on gene similarity.

In other words, it is required for a found genome to be similar to a known virus genome to classify it as an organism of that virus. This works by comparing the found genes to already known genes by creating an alignment. Examples of tools built based on similarity method are Kraken2 [10], Centrifuge [11], and FALCON-meta [12]. However, this method suffers from different shortcomings such as its poor ability to discover new viruses because no universal viral marker gene currently exists [13].

Another proposed method is called binning, which is implemented in tools such as MetaWatt [14] and CONCOT [15]. Binning introduces an additional step after the assembly method which groups the given contig into categories that correspond to a biological taxon—a classification used to denote a grouping of organisms, which can range from a single species to broader categories such as genus or family, reflecting various levels of the biological hierarchy [8].

In addition, classical sequence comparison approaches such as BLAST [16,17] have been employed (for a discussion, see [8]).

In addition to thes algorithmic approaches, researchers have also investigated the use machine learning (ML) approaches for classifying metagenomic sequences, due to the success of ML in various bioinformatics applications. Ren et al. [18] developed VirFinder, a logistic regression model to identify viral sequences given the genome. The proposed method can identify viruses within sequences of varying lengths, ranging from 500 to 10,000 bp. When testing VirFinder on NCBI data, however, it was found that VirFinder performs better on larger sequence lengths, which is often unrealistic. Expanding on this work, a new method called DeepVirFinder was introduced [19], serving the same objective as VirFinder. This deep-learning approach uses a convolutional neural network (CNN) to identify viral sequences within DNA viral sequences. Unlike the original VirFinder algorithm, DeepVirFinder has been improved to identify viral sequences with shorter lengths, specifically between 150 and 3,000 bp. Additionally, this method is capable of identifying viral sequences in real human gut metagenomic samples.

Several other deep-learning-based models employing different neuronal architectures have been developed for metagenomics-based tasks. Thus, Seeker [20] solves the problem of differentiating phage sequences from bacterial ones and is based on long-short-term memory (LSTM) architecture. Liu et al. [21] proposed RNN-VirSeeker, a deep-learning method for viral sequence identification, that also utilizes LSTMs and outperforms other state-of-the-art methods. PPR-META [22] is a deep-learning-based method that classifies contigs into phage, plasmid, and chromosome categories. The method was tested on artificial and real genomes of different lengths, ranging from 100 to 10,000 bp, and successfully outperformed other state-of-art methods. Finally, VIDHOP [23] is a method that can identify the original host of the virus in addition to the potential host genome. Two different deep-learning methods have proposed, the first one based on LSTM, while the other one combines LSTM with CNN.

Despite these advances, previous state-of-the-art methods often underperform, particularly in identifying new genomes, as we demonstrate in our benchmark based on a viral metagenomic dataset from infant guts. Additionally, authors fail to provide a method for cross-verifying classifications based on DNA sequences with those based on proteins derived from these DNA sequences. Our tool outperforms previous approaches in the mentioned benchmark and also introduces a method to classify protein sequences that vastly outperforms hidden Markov model (HMM)-based methods.

This study proposes a deep-learning-based method, HVSeeker, to enhance the identification of host and viral sequences in metagenomic data. We developed a robust approach for HVSeeker by testing different preprocessing methods and validating the performance across different degrees of homology. Combined with model architectures particular sensitive to sequence data, this allows us to create an approach that outperforms previous models. Experimental results demonstrate that HVSeeker can accurately identify both short and long host and viral sequences in metagenomes, outperforming a variety of widely used methods, e.g., Seeker, Rnn-VirSeeker, DeepVirFinder, and PPR-Meta.

## Methods

### Data collection and description

The research data for this study were gathered from well-known bioinformatics databases, including the National Center for Biotechnology Information (NCBI) [24] and the Integrated Microbial Genomes & Microbiomes—Viruses (IMGVR) [25,26]. The data consist of bacterial and phage DNA sequences. Each file contains a complete genome of different lengths and some metadata, e.g., the unique ID for each sequence and its length. The full dataset comprises 536 and 2,687 bacterial and phage sequences, respectively.

### Data preprocessing

The initial dataset consists of various DNA sequences encapsulated within FASTA files. These sequences vary in both biological origins and lengths. A preliminary task in our methodology involves the extraction of proteins from these diverse DNA sequences, which correspond to multiple classes. Thus, the input data for the subsequent training models constitute both DNA and protein sequences.

To ensure consistency in our data processing, we adjust the length of each DNA sequence to a uniform standard. In this process, we split the entire genome into segments of 1,000 bp, a procedure that often results in sequences shorter than the designated length as the genomes are usually not a multiple of our subsequence length (i.e., 1,000 bp).

To address this issue, we use three distinct strategies. The first strategy involves sequence padding, wherein we repetitively cycle through the sequence until it attains the required length. When padding sequences that are naturally shorter than the maximum number of base pairs, they are extended by duplicating their own sequence, therefore completing the short sequence by repeating it in the same order. The second strategy, which we termed the contigs assembly approach, is a two-fold process: we initially combine multiple shorter leftover sequences to generate a new, longer sequence, and split it into subsequences of our chosen length. Subsequently we apply padding to any residual segment that has not been incorporated into another sequence. The last strategy is a sliding-window process. In other words, for each sequence, we will select the first 1,000 bp, and continue the processes by moving the window by 100 bp until the end of the DNA. These techniques allow us to process input data of arbitrary length.

Upon achieving DNA sequences of uniform length, we proceed to eliminate any exact duplicate sequences.

Subsequently, we use one-hot encoding to transform the contigs from their original nucleotide form (adenine, cytosine, guanine, thymine, or ACGT) to a binary matrix of 0s and 1s. This conversion facilitates easier processing and interpretation of the genetic data by our computational model.

**Figure 1:**
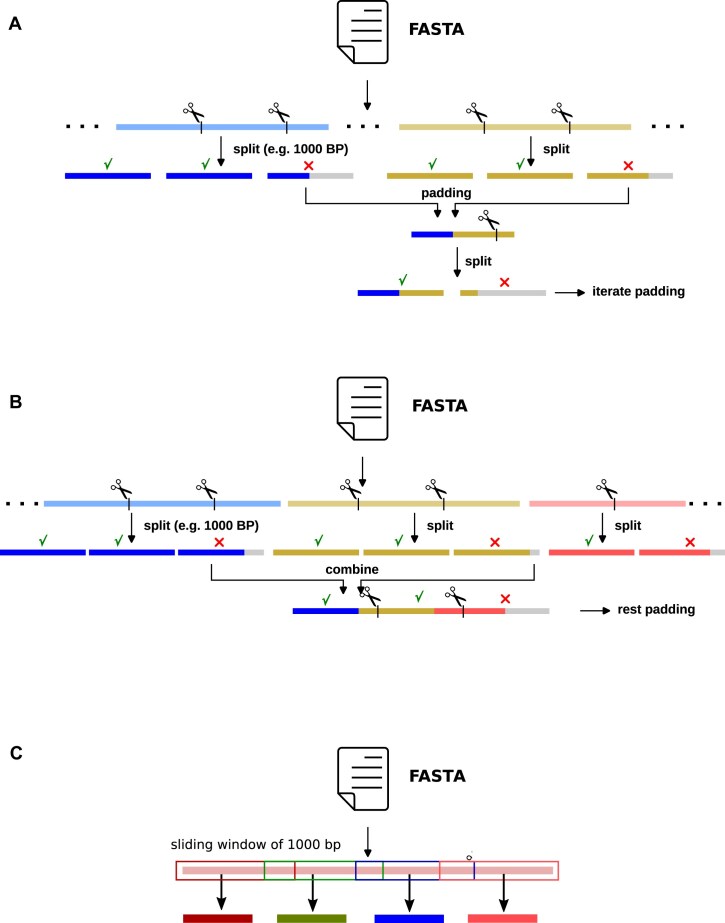
(A) Data preprocessing with padding. We cycle through the sequence until it attains the required length. (B) Data preprocessing with the contigs assembly method. Initially, we combine multiple shorter sequences to generate a longer sequence, which is then split again into subsequences of length 1,000 bp. Finally, we apply padding to any residual sequence. (C) Data preprocessing with the 1,000/100 bp sliding window method. We use a window of 1,000 bp to slide over the input DNA sequence in 100 bp steps.

In the final preprocessing step, we adopt an undersampling approach to balance the classes in our dataset. This procedure ensures that our model does not exhibit bias towards any particular class, leading to more reliable and generalizable predictions. With these steps, we conclude the preprocessing of the genomic sequence data. Following preprocessing, the dataset increased to 565,760 DNA sequences. For the model training process, we employed the holdout method, a recommended approach for larger datasets. The allocation of the data was set at 80% for the training set, with the remaining 20% equally split between validation and testing, at 10% each. To prevent overfitting, we used an early-stopping technique, which terminates the training when the performance on the validation set degrades. A visual summary of the can be found in Fig. 1A, B, and C.

Our model for classifying proteins, based on their amino acid sequences, uses a dataset comprising 98,720 unique phage sequences and 122,366 unique bacterial sequences. To ensure a broad representation of protein diversity, we employ BLAST to limit sequence homology between sets. This approach allows us to effectively capture the wide range of diversity found in bacterial and phage proteins. For testing purposes, we divided the data into 80% training data and 20% test data and repeated this process five times.

These steps encapsulate the preprocessing stage of our research which will create two datasets, one for HVSeeker-DNA and another for HVSeeker-Protein:

- Extraction of proteins from DNA sequences.
- Standardization of DNA sequence lengths to 1,000 bp.
- Handling of shorter sequences via padding, a combination of multiple sequences, or a sliding-window process.
- Removal of duplicate sequences.
- Transformation of nucleotide sequences to a binary matrix using one-hot encoding.
- Class balance in the dataset achieved through undersampling.

### The proposed models

To leverage the availability of both DNA and protein sequences, we develop two distinct models. The first model, called HVSeeker-DNA, is an LSTM-based model that takes as input DNA sequences with a length of 1,000 bp. LSTMs are primarily designed for sequential data where each input influences subsequent or preceding entries. Similarly, in DNA sequences, each nucleotide is related to its context, providing distinct meaning compared to individual elements. Therefore, we consider LSTMs to be an optimal choice for addressing DNA-related challenges. For the second one, HVSeeker-Protein, we adopt ProteinBERT [27] as a pretraining model and perform a fine-tuning phase using the constructed protein sequences. ProteinBERT has been trained on diverse protein sequence corpora in a self-supervised manner, making it possible to have a generic representation for every input protein sequence. In the following, each model will be described in detail.

#### HVSeeker-DNA structure

**Figure 2:**
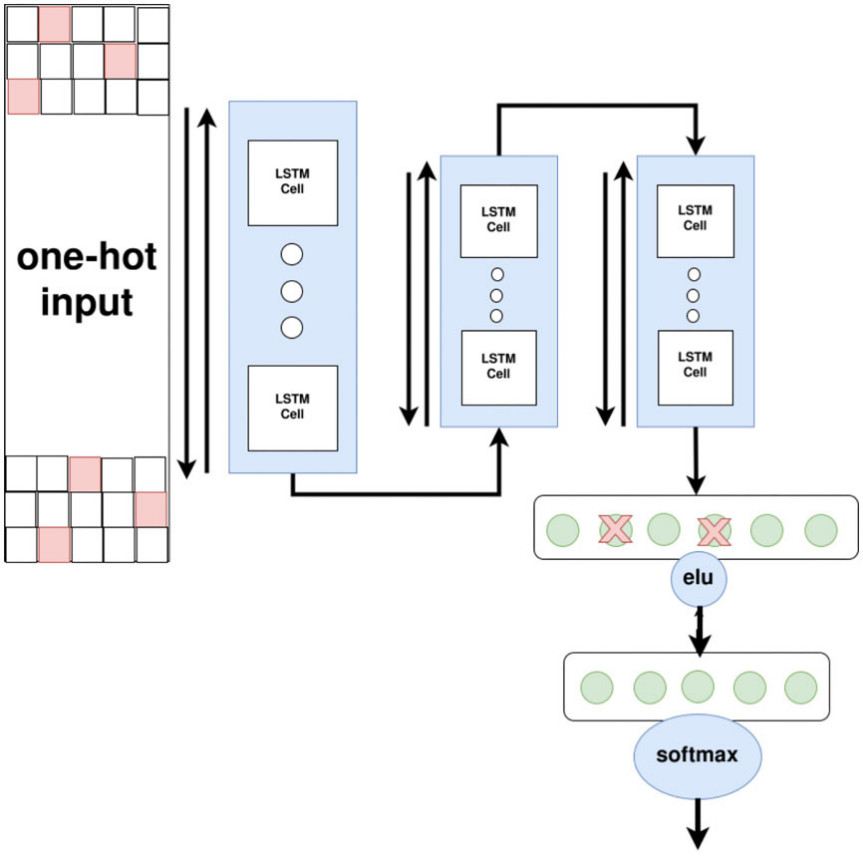
HVSeeker-DNA Architecture. First, the DNA data is encoded using different principles (padding, contigs-assembly, sliding window). We then process the one-hot encoded DNA using three bidirectional LSTM layers, followed by a linear layer with a moderate amount of dropout (0.2) and an elu activation function, and an output layer.

HVSeeker-DNA consists of three bidirectional connected LSTM units, followed by two fully connected layers, and then a softmax activation function for the final prediction. The first LSTM unit reads each data entry as a 6 × 1,000 matrix due to the one-hot encoding process, then it will output a vector of length 150 to be passed to the next two LSTM models. Then, a fully connected layer with the elu activation function and dropout of 0.2 will read the output of the last LSTM model and pass it to the next fully connected layer before using the softmax activation function for the final prediction. A visual summary of the model is presented in Fig. 2

#### HVSeeker-protein structure

In the event that the classification provided by the first model requires support with additional information about the proteins expressed by the DNA, we offer a second model that relies solely on the expressed protein sequences. To classify proteins as either phage or bacterial, we utilize embeddings generated by ProteinBERT. These embeddings accurately represent the input proteins due to the extensive dataset of training proteins used in ProteinBERT [27]. The model’s architecture includes a transformer with four attention heads and six layers, featuring a key size of 64. It has been trained on a large dataset of 160 million protein sequences, focusing on a reconstruction task. To enhance the fine-tuned model’s precision in predicting protein types, we employ a Bayesian optimizer. This optimizer uses an expected improvement acquisition function, initiating with eight random starts and conducting 25 evaluations on 1,000 proteins sampled from the training set. These evaluations fine-tune the learning rate, the number of training epochs, and the learning rate decay factor, optimizing the model’s performance.

To evaluate the effectiveness of the optimization and fine-tuning process, we conduct an assessment using five-fold cross-validation for the optimization process for five runs. For each test set, we use BLAST to ensure that sequence homology between the training and test set does not exceed 0.95. By employing this method, we can rigorously test the model across different subsets of the data, guaranteeing that our assessment of its performance is both fair and reliable.

### Evaluation criteria

To evaluate the effectiveness of the proposed models we focus on four key metrics: accuracy, precision, recall, and *F*1 score.

To evaluate model performance accuracy


(1) \begin{eqnarray*}
Accuracy = \frac{TP + TN}{TP + FP + TN + FN}
\end{eqnarray*}

is commonly used to calculate the ratio of correctly predicted instances to the total number of predictions.

However, accuracy can be misleading in the case of imbalanced datasets. Therefore, relying solely on accuracy is insufficient, and other metrics must be considered.

When wanting to focus on the positive class, precision


(2) \begin{eqnarray*}
Precision = \frac{TP}{TP + FP}
\end{eqnarray*}

can be used to measures the proportion of correctly predicted positive instances relative to all predicted positives.

Measuring the proportion of correctly predicted positive instances out of actual positives, recall


(3) \begin{eqnarray*}
Recall = \frac{TP}{TP + FN}
\end{eqnarray*}

is a crucial metric for addressing the impact of model bias in imbalanced datasets.

To balance precision and recall, the *F*1 measure


(4) \begin{eqnarray*}
F1\,\, measure = 2 \times \frac{Precision \times Recall}{Precision + Recall}
\end{eqnarray*}

provides a robust metric, offering a more comprehensive view of a model’s predictive strength.

## Results and discussion

To assess the effectiveness of the algorithm, four experiments were designed and executed. In the first experiment, we trained the model across a variety of sequence lengths to determine the optimal length for bacteriophage prediction. In the second experiment, we performed a self-comparison of our model under three distinct preprocessing conditions to understand the impact of these variations on the model’s performance. The third experiment compared our model with other models reported in the literature, on unseen data sequences from the same environment as our training set. The last experiment involved benchmarking our model against others in the literature using a standardized dataset. All the experiments are further discussed in the following sections.

### Optimizing sequence length for improved prediction performance

**Figure 3:**
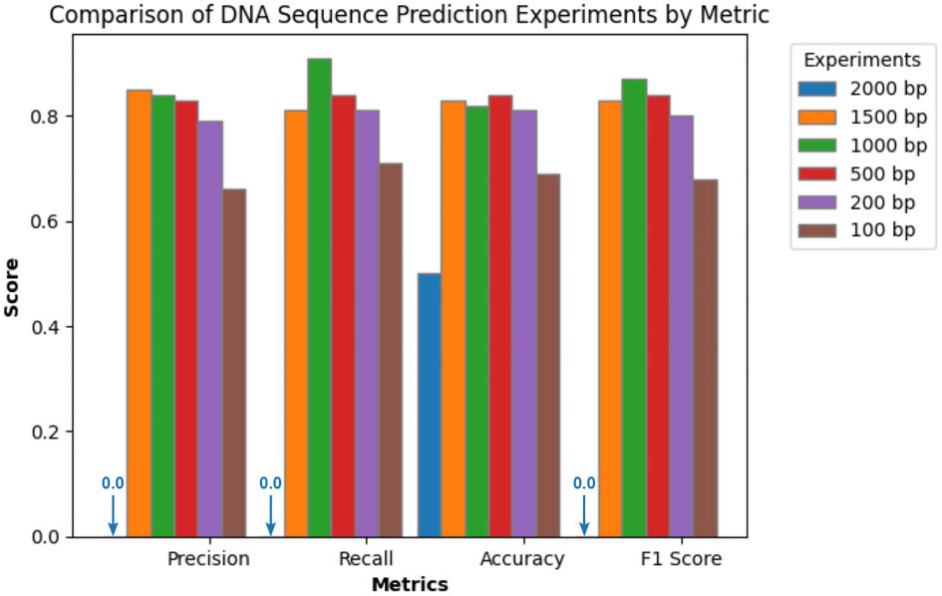
Comparison on different sequence lengths in terms of precision, recall, accuracy, and *F*1 score. We see the best performance in terms of the *F*1 score when using a length of 1,000 bp and the best performance in terms of recall for a length of 1,500 bp. Length 2,000 bp achieved a score 0 for all metrics except for accuracy due to overfitting. Here, HVSeeker potentially fails to capture local sequence properties as effectively as with smaller sequence sizes. Overall, we can conclude that excessively large sequence lengths overfit, while overly small sequence lengths underperformed.

To find the best sequence length for bacteriophage prediction and to assess model performance across different sequence lengths, we trained our model using sequences of 2,000, 1,500, 1,000, 500, 200, and 100 bp. We used padding as a preprocessing method for shorter sequences to ensure consistent input lengths. Figure 3 displays a comparative analysis of model performance, presenting precision, recall, accuracy, and *F*1 score for each evaluated sequence length. Figure 3 shows that the model trained on 2,000 bp sequences consistently predicted the bacteria class, indicating potential overfitting and an inability to generalize. This could be due to the longer sequence lengths introducing noise. On the other hand, the model trained on 1,000 bp sequences showed the best performance in terms of *F*1 score, outperforming other models with its recall rate, despite the 1,500 bp model achieving slightly higher precision. Conversely, the model with 100 bp sequences underperformed, as expected due to the reduced informational content of shorter sequences for bacteriophage identification. The findings also indicate that sequence lengths between 1,500 and 2,000 bp resulted in similar accuracy levels. This suggests that there is a threshold beyond which shorter sequence lengths start to noticeably affect model performance. Based on these results, subsequent experiments focused solely on the 1,000 bp sequence length, which demonstrated the most balanced performance across all evaluated metrics.

### Evaluating the impact of preprocessing techniques on model accuracy

**Figure 4:**
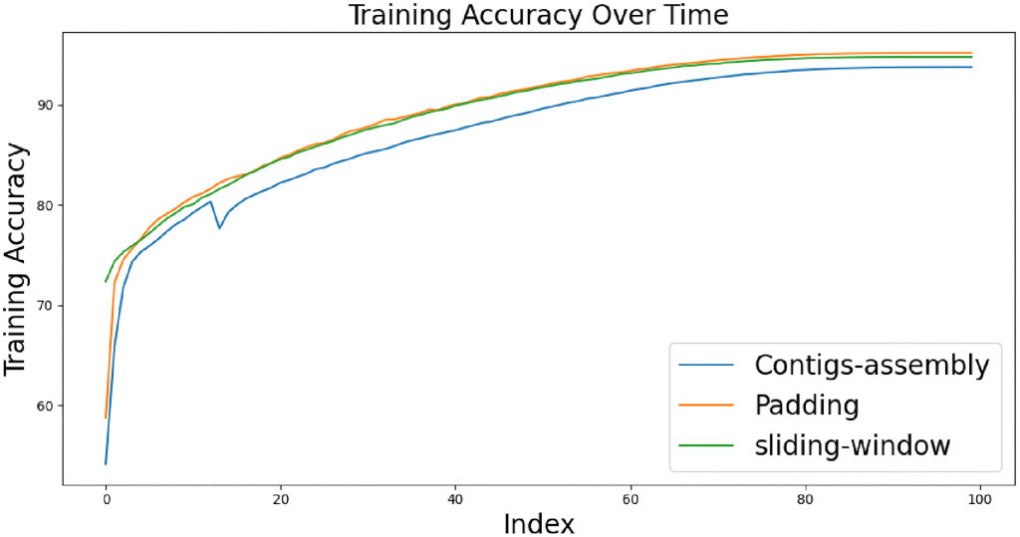
Training accuracy of the three models. All models converge relatively quickly to their maximal performance with small differences between the accuracies of the embedding methods.

**Figure 5:**
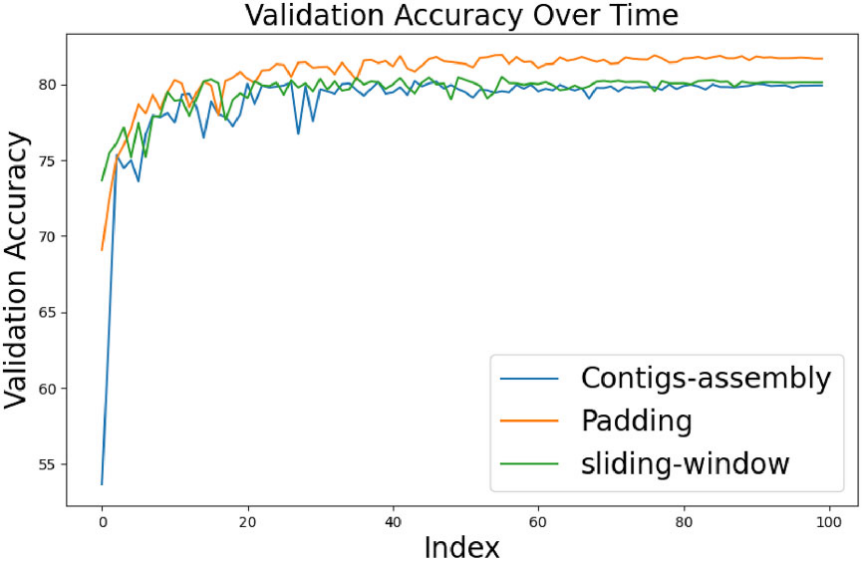
Validation accuracy of the three models. All models converge relatively quickly to their maximal performance with small differences between the accuracies of the embedding methods.

In the Methods, we introduced three data preprocessing methods: padding, assembly of shorter sequences method, and sliding windows. To assess the impact of each preprocessing technique, we trained a separate model for each method. The training and validation accuracies of these models are shown in Figs 4 and 5 respectively. According to these figures, the sliding-window method initially led to higher accuracy during the early training epochs. However, as training progressed, all three models converged to comparable levels of accuracy for both the training and validation phases. Despite the overall similarity in performance, a closer inspection reveals that the padding method slightly outperformed the others in terms of validation accuracy, whereas the assembly of contigs method was slightly behind. This could be attributed to the padding method’s tendency to duplicate nucleotides, potentially providing the model with more consistent training data. In contrast, the assembly method, which might combine sequences from varied origins, could introduce a higher degree of variability and confusion to the model’s learning process. Eventually, the three models achieved validation accuracy exceeding 80%, which indicates a robust model architecture capable of adapting to various preprocessing strategies. Another interesting observation is that all three methods achieved similar scores across all metrics, unlike others that may be biased towards recall or precision, such as DeepVirFinder or PPR-Meta. This consistency could be attributed to the balanced dataset used for training HVSeeker, highlighting the importance of the undersampling step in preprocessing. Additionally, this demonstrates the robustness of HVSeeker’s preprocessing and architecture, achieving an excellent balance between generalization and adapting to the training data.

### Benchmarking the proposed method against existing tools

To extend our evaluation of the proposed models, we compared them with existing bacteriophage classification approaches found in the literature, specifically Seeker [20], Rnn-VirSeeker [21], DeepVirFinder [19], and PPR-Meta [22]. For a fair comparison, we ensured that each algorithm was trained using the same dataset introduced in our study. Table 1 outlines the final results of each model on unseen testing data. As shown in the table, the three proposed models outperformed both Seeker and Rnn-VirSeeker in terms of precision, recall, accuracy, and *F*1 score. While both DeepVirFinder and PPR-Meta achieved higher precision score, they achieved lower recall, accuracy, and *F*1 score. Notably, all the models posted comparable results across various evaluation metrics, suggesting that our model construction was robust enough to mitigate potential biases. Conversely, Seeker’s model yielded an *F*1 score of approximately 58%, hinting at a need for more extensive data to enhance its performance. In contrast, Rnn-VirSeeker appeared to struggle with underfitting, being unable to adapt to the complexity of the training data; it only achieved an accuracy of roughly 50% on the training data. This underperformance could stem from the label-encoding technique RNN-Seeker uses to encode its data, which assigns numerical values to nucleotides. This method is generally not recommended because it can prioritize some features over others and suggests an order between nucleotides, generally not found in the natural world, which potentially skews the data analysis and interpretation. In the case of DeepVirFinder and PPR-Meta, these models exhibit high precision but low recall. This indicates that while they are correctly identifying phages, many actual phage genomes remain undetected, thus lacking comprehensive accuracy and *F*1 score in phage prediction.

**Table 1:** Comparison of the proposed approach with different preprocessing methods against Seeker, Rnn-seeker DeepVirFinder, and PPR-Meta. We find that our method outperforms others in most of the used metrics, specifically in accuracy and *F*1 score, regardless of the input embedding used. Using padding and a sliding-window approach both result in similar good results, with both methods showing a slight advantage over contigs assembly.

	Precision	Recall	Accuracy	*F*1 score
Padding	83.72%	**90.07%**	**87.25%**	**86.78%**
Contigs assembly	80.76%	80.02%	80.30%	80.39%
Sliding window	83.40%	82.43%	82.81%	82.91%
Seeker	56.98%	58.95%	59.39%	57.95%
Rnn-VirSeeker	00.00%	00.00%	50.00%	00.00%
DeepVirFinder	90.50%	65.14%	79.23%	75.75%
PPR-Meta	**92.00%**	67.00%	81.00%	77.00%

### Performance on low-homology datasets

To challenge the generalization ability of our model, we use our previously created test set and remove all sequences above a certain homology cut-off by using BLAST to compare the sequences to our training set. Based on the resulting alignment files, we create five different homology datasets. The results for all datasets can be found in Supplementary Figs S3–S7. HVSeeker consistently achieves the best *F*1 score for all data splits. When using a homology cut-off of 60%, HVSeeker still achieves an *F*1 score of 68.60% with a precision of 57.70% and a recall of 84.80%. Other methods usually do not balance precision and recall as well, resulting in considerably lower *F*1 scores. The scores can be found in Table 2.

**Table 2:** Comparsion of the proposed approach with previous methods against Seeker, Rnn-seeker DeepVirFinder, and PPR-Meta on a low-homology dataset of 60% maximum homology. We find HVSeeker achieves the highest *F*1 core with 68.60%, roughly 10% better than the second-best method DeepVirFinder with 57.06%. Additionally used low-homology sets can also be found in the Supplementary Tables S3–S7.

	Precision	Recall	Accuracy	*F*1 score
HVSeeker	57.70%	84.80%	70.30%	**68.60**%
Seeker	38.29%	**98.51%**	38.57%	55.15%
Rnn-VirSeeker	00.00%	00.00%	62.00%	00.00%
DeepVirFinder	63.33%	51.93%	70.03%	57.06%
PPR-Meta	**78.00%**	44.00%	**74.00%**	56.00%

### Performance on a diverse viral metagenomic dataset from an infant gut metagenomic dataset

A key challenge in bacteriophage detection is the recognition of new sequence patterns. To evaluate our algorithm’s capability in this regard, we conducted a performance comparison using benchmark datasets collected from [28] with Seeker, Rnn-VirSeeker, DeepVirFinder, and PPR-Meta. The study analyzed viral diversity in the fecal viromes of 647 one-year-olds from the Copenhagen Prospective Studies on Asthma in Childhood 2010 (COPSAC2010). Fecal samples were successfully collected and viromes were characterized for 647 children at one year of age, with metagenomes sequenced in parallel. The study’s authors identified 10,000 viral species from 248 virus-family-level clades, with 232 being newly discovered, primarily from the Caudoviricetes class. Hosts for 79% of the phages were determined using CRISPR spacers from bacterial metagenomes of the same children. The results of our comparison are detailed in Table 3. The proposed algorithm consistently outperformed all methods across all evaluated metrics except accuracy. Notably, our algorithm demonstrated a greater recall relative to precision, which can be attributed to the benchmark dataset’s composition—featuring a higher count of negative than positive instances. With a final *F*1 score of 0.767, closely mirroring its performance on testing data, our model proves its proficiency in accurately processing sequences from varied environments, comparably to how it performs with the training set’s environment. Conversely, Seeker, with an *F*1 score of 0.578, DeepVirFinder with an *F*1 score of 0.417, and PPR-Meta with an *F*1 score of 44, adapt to the data during training only to a limited extent, suggesting either insufficient data or inadequate model complexity. Meanwhile, Rnn-VirSeeker exhibited underfitting, indicating a failure to learn effectively from the training data.

**Table 3:** Comparison on benchmark data. Again HVSeeker outperforms the alternative method Seeker by an impressive margin of 18.9 for the *F*1 score and 18.83 in terms of the classification accuracy. Meanwhile, Rnn-VirSeeker seems to be unable to learn properly from the provided data.

	Precision	Recall	Accuracy	*F*1 score
HVSeeker	**67.01%**	**89.74%**	65.23%	**76.73%**
Seeker	42.92%	88.43%	46.40%	57.79%
Rnn-VirSeeker	00.00%	00.00%	14.46%	00.00%
DeepVirFinder	32.18%	59.04%	74.22%	41.66%
PPR-Meta	34.00%	61.00%	**77.00%**	44.00%

### Protein-based classification

We report the average performance over all five runs of our method assessed on our test sets, achieving an average area under the curve (AUC) value of 0.89. This indicates a strong performance in the protein-based classification task. Additionally, the classification accuracy across all splits is 82%, further demonstrating the method’s effectiveness. The weighted *F*1 score stands at 0.82, with a weighted precision of 0.82 and a weighted recall of 0.82. The additional information created by this classification mechanism can be used reliably to support the prediction created purely based on the DNA. For the minority of the sequences of a length from 571 to 1,144 we find a similar AUC value of 0.88, indicating the stability of the results for greater sequence lengths. The weighted *F*1 score stands at 0.84, with a weighted precision of 0.84 and a weighted recall of 0.84. To compare our method with a baseline approach, we constructed HMMs for all five subsets of the training dataset. Initially, we generated multiple sequence alignments using MAFFT (FFT-NS-2)  [29], followed by the construction of HMMs with HMMbuild  [30]. The subsequent search over the test data yielded only twelve correct hits above the standard reporting threshold. This result indicates the model’s inability to adequately represent the diversity of the input data, translating to an accuracy of approximately 0.0051%.

## Conclusion

In this study, we introduced HVSeeker, a novel methodology designed to differentiate between viral and bacterial genomic sequences. HVSeeker surpasses existing state-of-the-art methods, e.g., DeepVirFinder, PPR-Meta, RNN-VirSeeker, and Seeker, in performance on two benchmarks and additionally evaluates corresponding proteins, offering improved insights. Unlike previous methods HVSeeker can work with DNA input as well as protein inputs, allowing researchers to combine evidence for both. This advancement is important for accurately identifying viral genomes and therefore the creation of new therapeutic approaches such as phage therapy. Identifying the host and viral sequences in mixed metagenomes is the initial step toward analyzing the host viral component of samples. This process is crucial for downstream work. HVseeker should prove to be an essential tool in prokaryotic and phage taxonomy, as well as in bacterial–host interactions.

## Availability of source code and requirements

- Project name: HVSeeker [31]
- Project home page:  https://github.com/BackofenLab/HVSeeker
- Programming language: Python
- Other requirements: To install software requirements we provide a conda environment file for HVSeeker-DNA and HVSeeker-Protein in the github repo
- License: MIT licence
- in bio.tools registered as hvseeker
- rrid: SCR_026120
- An archival copy of the github repository is available via software heritage [32]
- See DOME-ML registry for more details [33]
- Additionally DNA sample data [34] [ABVK02000003,ABZV01000005, ACKO02000018, AFMC02000011, AFSV01000040, CP000303, CP000304,CP000386, CP000387, NC_000866, NC_000867, NC_001317, NC_001330 ] and protein sample data [35] are available online.

## Supplementary Material

giaf037_Supplemental_File

giaf037_Authors_Response_To_Reviewer_Comments_Original_Submission

giaf037_Authors_Response_To_Reviewer_Comments_Revision_1

giaf037_Authors_Response_To_Reviewer_Comments_Revision_2

giaf037_Authors_Response_To_Reviewer_Comments_Revision_3

giaf037_Authors_Response_To_Reviewer_Comments_Revision_4

giaf037_GIGA-D-24-00282_Original_Submission

giaf037_GIGA-D-24-00282_Revision_1

giaf037_GIGA-D-24-00282_Revision_2

giaf037_GIGA-D-24-00282_Revision_3

giaf037_GIGA-D-24-00282_Revision_4

giaf037_GIGA-D-24-00282_Revision_5

giaf037_Reviewer_1_Report_Original_Submission Diogo Pratas -- 8/1/2024

giaf037_Reviewer_1_Report_Revision_1 Diogo Pratas -- 10/22/2024

giaf037_Reviewer_2_Report_Original_Submission Satoshi Hiraoka -- 8/18/2024

giaf037_Reviewer_2_Report_Revision_1 Satoshi Hiraoka -- 10/19/2024

## Data Availability

The model used is available under https://github.com/BackofenLab/HVSeeker [31]. DOME-ML annotations are available via the DOME registry under accession igr5x3a1vs [33].

## Additional Files

**Supplementary Table S1**: Comperative performance metrics of sequence identification methods on test set.

**Supplementary Table S2**: Performance metrics of HVSeeker, Seeker, and Rnn-VirSeeker on unseen benchmark dataset.

**Supplementary Table S3**: Comparative performance metrics of sequence identification methods on test dataset with a maximum of 95% homology.

**Supplementary Table S4**: Comparative performance metrics of sequence identification methods on test dataset with a maximum of 90% homology.

**Supplementary Table S5**: Comparative performance metrics of sequence identification methods on test dataset with a maximum of 80% homology.

**Supplementary Table S6**: Comparative performance metrics of sequence identification methods on test dataset with a maximum of 70% homology.

**Supplementary Table S7**: Comparative performance metrics of sequence identification methods on test dataset with a maximum of 60% homology.

## Abbreviations

CNN: convolutional neural network; HMM: hidden Markov model; IMGVR: Integrated Microbial; LSTM: long-short-term-memory; ML: machine-learning; NCBI: National Center for Biotechnology Information; Pfam: protein family.
