# Supplementary material for: HVSeeker: a deep-learning-based method for identification of host and viral DNA sequences
Source: Gigascience. 2025 May 15;14:giaf037. doi: 10.1093/gigascience/giaf037 (PMC12080225; doi:10.1093/gigascience/giaf037)
Supplement: giaf037_Supplemental_File [file giaf037_supplemental_file.pdf]

Overview

Metagenome classification is essential for advancements in health and ecology. Despite progress in machine learning reducing time and cost, the analysis of short genetic sequences from environmental samples remains a challenge. Our study presents HVSeeker, a deep learning tool designed to refine the detection of host and viral sequences within metagenomic datasets. HVSeeker successfully can identify both short and longer sequences. This document outlines the benchmarking of HVSeeker against existing methods, namely Seeker and RNN-VirSeeker, to showcase its efficacy and accuracy in sequence identification.

HVSeeker

HVSeeker is introduced in two variants: HVSeeker-DNA and HVSeeker-Protein. Since both Seeker and Rnn-VirSeeker are DNA-based models, we benchmark HVSeeker-DNA against them only.

HVSeeker-DNA

- Architecture consisting of three bidirectional LSTM (Long Short-Term Memory) units connected sequentially.
- Followed by two densely connected layers, culminating in a softmax activation function for final sequence classification.
- Each input sequence is transformed into a 6×1000 matrix through one-hot encoding, which is then processed by the first LSTM unit to produce a 150-length vector.
- This vector is sequentially passed through the remaining LSTM units and a fully connected layer with an ELU (Exponential Linear Unit) activation function and a dropout rate of 0.2 to prevent overfitting.
- The final layer uses a softmax activation function to generate the prediction probabilities.

Training Parameters

- The model undergoes training for 100 epochs
- A batch size of 64 is used
- The Adam optimizer is chosen, with 0.001 learning rate being employed

Seeker

The Seeker model is part of an earlier work focused on the identification of viral contigs. Seeker is an alignment-free tool that distinguishes between bacterial and phage DNA sequences using a deep learning framework. The model is designed to provide accurate classification without the need for traditional sequence alignment methods. For more information on the Seeker model and to access its codebase, visit the following GitHub repository: https://github.com/gussow/seeker/tree/master

Rnn-VirSeeker

The RNN-VirSeeker is a deep learning based model for the identification of viral contigs from metagenomic datasets in FASTA format. It is particularly adept at recognizing viral sequences even when they are as short as 500 base pairs, which is often a challenge in metagenomics. To explore the RNN-VirSeeker model, refer to its GitHub repository: https://github.com/crazyinter/RNN-VirSeeker/tree/master

DeepVirFinder

DeepVirFinder is a deep learning-based tool that detects viral sequences in metagenomic data. It leverages advanced neural network architectures to distinguish viral sequences from non-viral sequences. To further explore DeepVirFinder refer to the following repostery: https://github.com/jessieren/DeepVirFinder

PPR-Meta

PPR-Meta is a computational tool designed for classifying metagenomic sequences into three categories: prokaryotic, phage, and plasmid. This tool employs deep learning techniques to enhance the accuracy of classification. To further know about PPR-Meta visit the repostry: https://github.com/zhenchengfang/PPR-Meta

benchmarking procedure

The performance of our model is evaluated using widely recognized metrics that provide insights into its classification accuracy and reliability.

Evaluation Criteria
The following metrics are used to assess the model's effectiveness:

- Accuracy: The ratio of correctly predicted observations to the total observations
- Precision: The ratio of correctly predicted positive observations to the total predicted positive observations
- Recall (Sensitivity): The ratio of correctly predicted positive observations to all actual positives
- F1 Score: The weighted average of Precision and Recall, where the best score is 1 and the worst is 0

Dataset Preparation
The dataset comprises 565,760 DNA sequences:

- Training Set: 452,608 sequences (80% of the dataset) are used for training the model.
- Validation Set: 56,576 sequences (10% of the dataset) are used to fine-tune the model parameters.
- Testing Set: 56,576 sequences (10% of the dataset) are used for the final evaluation of the model.

Training Seeker model steps
- **Prepare Dataset:** Execute the dataset_creator.py script to format the dataset appropriately for Seeker
- **Train Model:** Run the train_model.py script to start the training process. The model will be trained for 100 epochs using a batch size of 27. python train_model.py –bacteria sample_bacteria_training.txt –phage sample_phage_training.txt –out sample_model.h5
- **Save Model:** The trained model will be saved in the same directory with the specified output file name (e.g., sample_model.h5).
- **Test Model:** Use the test_model.py file to load the trained model and assess its performance on the test dataset. This step will generate the accuracy metrics and a confusion matrix visualization.

After following these steps, you will have a trained Seeker model ready for evaluating its performance on identifying viral contigs from metagenomic data.

Training Rnn-VirSeeker steps:
- **Prepare Dataset:** Run the rnn_seeker_dataset_creator.py file to format the dataset for Rnn-VirSeeker.

- **Prepare Output Format:** Execute the rnn_seeker_output_creator.py script to format the output for Rnn-VirSeeker.
- **Train the Model:** Use the train.py script to start the training process. The model will undergo 100 epochs with a batch size of 256.
- **Save the Model:** The trained model will be saved automatically in the same directory
- **Test the Model:** Run the test.py script to load the trained model and evaluate its accuracy on the test data. This will also generate a confusion matrix

Results

The table below presents a detailed comparison of the performance metrics for HVSeeker, Seeker, and Rnn-VirSeeker. These metrics include Precision, Recall, Accuracy, and F1-Score

Method	Precision	Recall	Accuracy	F1-Score
Padding	83.91%	90.84%	82.01%	87.23%
Contigs-assembly 80.76%	80.02%	80.30%	80.39%	
Sliding-window	83.40%	82.43%	82.81%	82.91%
Seeker	56.98%	58.95%	59.39%	57.95%
Rnn-VirSeeker	0%	0%	50%	0%

Table 1. Comparative performance metrics of sequence identification methods on test dataset

Method	Precision	Recall	Accuracy	F1-Score
HVSeeker	67.01%	89.74%	65.23%	76.73%
Seeker	42.92%	88.43%	46.40%	57.79%
Rnn-VirSeeker	0%	0%	14.46%	0%

Table 2. Comparative performance metrics of sequence identification methods on test dataset

The tables clearly indicates that HVSeeker outperforms the other tools across all measured metrics. HVSeeker's F1-score is particularly noteworthy, demonstrating its ability to accurately identify relevant sequences while minimizing false positives.

results of tools in datasets of limited homology

To test the abilities of different models to generalize to data very much unlike the training set, we sepearted the data according to different homology conditions using BLAST. This results in different homology subsets, ranking from a maximum of 95% to 60%.

	Precision	Recall	Accuracy	F1-Score
HVSeeker	60.60%	86.20%	73.10%	**71.10%**
Seeker	38.45%	**98.69%**	38.72%	55.34%
Rnn-VirSeeker	0%	0%	62%	0%
DeepVirFinder	69.98%	57.37%	74.13%	63.05%
PPR-Meta	**82%**	51%	**77%**	63%

Table 3. Comparative performance metrics of sequence identification methods on test dataset with a maximum of 95% homology

	Precision	Recall	Accuracy	F1-Score
HVSeeker	59.10%	85.50%	72.40%	**69.80%**
Seeker	37.34%	**98.71%**	37.65%	54.19%
Rnn-VirSeeker	0%	0%	63%	0%
DeepVirFinder	68.12%	55.24%	72.88%	61.06%
PPR-Meta	**80%**	46%	**75%**	58%

Table 4. Comparative performance metrics of sequence identification methods on test dataset with a maximum of 90% homology

	Precision	Recall	Accuracy	F1-Score
HVSeeker	58.50%	84.50%	71.10%	**69.10%**
Seeker	38.18%	**98.64%**	38.74%	55.05%
Rnn-VirSeeker	0%	0%	62%	0%
DeepVirFinder	64.46%	50.14%	70.38%	56.40%
PPR-Meta	**79%**	43%	**74%**	55%

Table 5. Comparative performance metrics of sequence identification methods on test dataset with a maximum of 80% homology

Conclusion

In summary, the comparison of different tools for identifying sequences shows that HVSeeker outperforms the others with the highest scores in all evaluation metrics. On the other hand, Seeker has moderate performance, and Rnn-VirSeeker does not perform well at all, with zero scores in most categories. These results highlight that the choice of tool can significantly impact the success of sequence identification tasks. Padding is the most reliable method based on this comparison. As technology advances, efforts should be made to improve these tools for better accuracy and efficiency in bioinformatics.

For more details please refer to: https://github.com/bulatef/HVSeeker/tree/main/Supplementary

	Precision	Recall	Accuracy	F1-Score
HVSeeker	57.70%	84.80%	70.40%	**68.60%**
Seeker	38.28%	**98.51%**	38.56%	55.14%
Rnn-VirSeeker	0%	0%	61%	0%
DeepVirFinder	63.33%	51.54%	69.98%	56.83%
PPR-Meta	**78%**	43%	**74%**	56%

Table 6. Comparative performance metrics of sequence identification methods on test dataset with a maximum of 70% homology

	Precision	Recall	Accuracy	F1-Score
HVSeeker	57.70%	84.80%	70.30%	**68.60%**
Seeker	38.29%	**98.51%**	38.57%	55.15%
Rnn-VirSeeker	0%	0%	62%	0%
DeepVirFinder	63.33%	51.93%	70.03%	57.06%
PPR-Meta	**78%**	44%	**74%**	56%

Table 7. Comparative performance metrics of sequence identification methods on test dataset with a maximum of 60% homology
