# Supplementary material for: HVSeeker: a deep-learning-based method for identification of host and viral DNA sequences
Source: Gigascience. 2025 May 15;14:giaf037. doi: 10.1093/gigascience/giaf037 (PMC12080225; doi:10.1093/gigascience/giaf037)
Supplement: giaf037_GIGA-D-24-00282_Revision_3 [file giaf037_giga-d-24-00282_revision_3.pdf]

GigaScience

HVSeeker: A Deep Learning-based Method for Identification of Host and Viral DNA sequences

--Manuscript Draft--

**Manuscript Number:**	GIGA-D-24-00282R3
**Full Title:**	HVSeeker: A Deep Learning-based Method for Identification of Host and Viral DNA sequences
**Article Type:**	Technical Note
**Funding Information:**	Deutsche Forschungsgemeinschaft ([BA 2168/23-1/2) — Prof Rolf Backofen
**Abstract:**	Motivation: Bacteriophages are among the most abundant organisms on Earth, significantly impacting ecosystems and human society. The identification of viral sequences, especially novel ones, from mixed metagenomes is a critical first step in analyzing the viral components of host samples. This plays a key role in many downstream tasks. However, this is a challenging task due to their rapid evolution rate. The identification process typically involves two steps: distinguishing viral sequences from the host and identifying if they come from novel viral genomes. Traditional metagenomic techniques that rely on sequence similarity with known entities often fall short, especially when dealing with short or novel genomes. Meanwhile, deep learning has demonstrated its efficacy across various domains, including the Bioinformatics field.<br>Results: We have developed HVSeeker, a deep learning-based method for distinguishing between bacterial and phage sequences. HVSeeker consists of two separate models: one analyzing DNA sequences and the other focusing on proteins. This method has shown promising results on sequences with various lengths, ranging from 200 to 1500 base pairs. Tested on both NCBI and IMGVR databases, HVSeeker outperformed several methods from the literature such as Seeker, Rnn-VirSeeker, DeepVirFinder, and PPR-Meta. Moreover, when compared with other methods on benchmark datasets, HVSeeker has shown better performance, establishing its effectiveness in identifying unknown phage genomes.<br>Availability: A Python implementation of HVseeker and the Python code developed for this study have been provided on Github https://github.com/bulatef/HVSeeker.
**Corresponding Author:**	Omer Alkhnbashi, PhD<br>MBRU: Mohammed Bin Rashid University of Medicine and Health Sciences<br>Dubai, UNITED ARAB EMIRATES
**Corresponding Author Secondary Information:**	
**Corresponding Author's Institution:**	MBRU: Mohammed Bin Rashid University of Medicine and Health Sciences
**Corresponding Author's Secondary Institution:**	
**First Author:**	Omer Alkhnbashi, PhD
**First Author Secondary Information:**	
**Order of Authors:**	Omer Alkhnbashi, PhD
	Abdullatif Al-Najim
	Sven Hauns
	Rolf Backofen
	Van Dinh Tran
**Order of Authors Secondary Information:**	
**Response to Reviewers:**	Dear Dr. Hans<br>Editor-in-Chief of GiGascience

Powered by Editorial Manager® and ProduXion Manager® from Aries Systems Corporation

	Herewith we submit the revised version of our manuscript “HVSeeker: A Deep Learning-based Method for Identification of Host and Viral DNA sequences”.<br><br>We have answered each point and have modified our submission accordingly. We have also carefully considered the reviewer’s comments and integrated them into a thoroughly revised manuscript version.<br><br>We thank the reviewers for their supportive comments and constructive feedback and the editor for the opportunity to submit a revised manuscript. Our response to each comment below is in blue.<br><br>I am looking forward to hearing from you.<br><br>Sincerely yours,
**Additional Information:**	
**Question**	**Response**
Are you submitting this manuscript to a special series or article collection?	No
**Experimental design and statistics**<br><br>Full details of the experimental design and statistical methods used should be given in the Methods section, as detailed in our Minimum Standards Reporting Checklist. Information essential to interpreting the data presented should be made available in the figure legends.<br><br>Have you included all the information requested in your manuscript?	Yes
**Resources**<br><br>A description of all resources used, including antibodies, cell lines, animals and software tools, with enough information to allow them to be uniquely identified, should be included in the Methods section. Authors are strongly encouraged to cite Research Resource Identifiers (RRIDs) for antibodies, model organisms and tools, where possible.<br><br>Have you included the information requested as detailed in our Minimum	Yes

Powered by Editorial Manager® and ProduXion Manager® from Aries Systems Corporation

Standards Reporting Checklist?	
**Availability of data and materials**<br><br>All datasets and code on which the conclusions of the paper rely must be either included in your submission or deposited in publicly available repositories (where available and ethically appropriate), referencing such data using a unique identifier in the references and in the “Availability of Data and Materials” section of your manuscript.<br><br>Have you have met the above requirement as detailed in our Minimum Standards Reporting Checklist?	Yes

Manuscript
Click here to access/download;Manuscript;HVSeeke-Manuscript.pdf

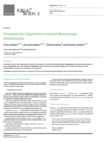

GigaScience, 2023, 1–8

doi: xx.xxxx/xxxx
Manuscript in Preparation
Paper

PAPER

HVSeeker: A Deep Learning-based Method for Identification of Host and Viral DNA sequences

Abdullatif Al-Najim[1,†], Sven Hauns[2,†], Van Dinh Tran[1], Rolf Backofen[2, 3,*] and Omer S. Alkhnbashi[4, 5,*]

[1]Information and Computer Science department, King Fahd University of Petroleum and Minerals, Dhahran, 34462, Saudi Arabia and [2]Bioinformatics group,Department of Computer Science, University of Freiburg,Georges-Köhler-Allee 101,79110,Germany and [3]Signalling Research Centres BIOSS and CIBSS, University of Freiburg, Schänzlestr. 18, 79104 Freiburg, Germany and [4]Center for Applied and Translational Genomics (CATG) and [5]College of Medicine, Mohammed Bin Rashid University of Medicine and Health Sciences, Dubai Healthcare City, Dubai, United Arab Emirates

[*]To whom correspondence should be addressed: omer.alkhnbashi@dubaihealth.ae;backofen@informatik.uni-freiburg.de
[†] shared first authorship

Abstract

Background Bacteriophages are among the most abundant organisms on Earth, significantly impacting ecosystems and human society. The identification of viral sequences, especially novel ones, from mixed metagenomes is a critical first step in analyzing the viral components of host samples. This plays a key role in many downstream tasks. However, this is a challenging task due to their rapid evolution rate. The identification process typically involves two steps: distinguishing viral sequences from the host and identifying if they come from novel viral genomes. Traditional metagenomic techniques that rely on sequence similarity with known entities often fall short, especially when dealing with short or novel genomes. Meanwhile, deep learning has demonstrated its efficacy across various domains, including the Bioinformatics field.
Results We have developed HVSeeker - a host/virus seeker method - based on deep learning for distinguishing between bacterial and phage sequences. HVSeeker consists of two separate models: one analyzing DNA sequences and the other focusing on proteins. In addition to the robust architecture of HVSeeker, three distinct preprocessing methods were introduced to enhance the learning process: padding, contigs assembly, and sliding window. This method has shown promising results on sequences with various lengths, ranging from 200 to 1500 base pairs. Tested on both NCBI and IMGVR databases, HVSeeker outperformed several methods from the literature such as Seeker, Rnn-VirSeeker, DeepVirFinder, and PPR-Meta. Moreover, when compared with other methods on benchmark datasets, HVSeeker has shown better performance, establishing its effectiveness in identifying unknown phage genomes.
Conclusions These results demonstrate the exceptional structure of HVSeeker, which encompasses both the preprocessing methods and the model design. The advancements provided by HVSeeker are significant for identifying viral genomes and developing new therapeutic approaches, such as phage therapy. Therefore, HVSeeker serves as an essential tool in prokaryotic and phage taxonomy, offering a crucial first step toward analyzing the host-viral component of samples by identifying the host and viral sequences in mixed metagenomes.

Key words: Genomics, Bacteria, Phages, Deep Learning

Introduction

Viruses, the most common organisms on the planet [1], significantly affect both ecosystems and human health [2]. Capable of

Compiled on: December 23, 2024.
Draft manuscript prepared by the author.

Key Points

- We introduce HVSeeker , a novel DeepLearning method for classification of bacteria and phage genomes.
- We create three different strategies for creating genomic input sequences and benchmark their effectiveness.
- Data preprocessing with padding achieved better results than using contigs assembly or a sliding window
- HVSeeker compares favorably to alternative classification method, Seeker, Rnn-VirSeeker,DeepVirFinder, PPR-Meta, even on low-homology datasets.
- Additionally we finetune a small ProtBert based model to provide an additional mechanism to evaluate genomic sequences.

infecting a wide range of species, including humans and bacteria, viruses exert a profound effect on bacteria populations. Phages, which specifically target bacteria, have a meaningful impact on their host and are also influencing human health. This underscores the importance of the role that the interaction between bacteria and phages has on humans [3]. Phages infect bacteria by first injecting their viral DNA into the bacteria, after breaking down the cell wall with endolysins. In the next step, the phage DNA either integrates into the bacterial DNA or initiates a lytic cycle, using the bacterial replication instruments to reproduce its DNA [4]. Ultimately, the viral genome and proteins assemble to form a new virion [4].

Due to the constant competition between phages and bacteria, the former have developed defense mechanisms that can be used as an alternative to antibiotics [5]. Furthermore, due to their ability to lyse bacterial cell walls, phage-derived endolysins were suggested as an antimicrobial agent [6]. For that purpose, the phage-bacteria relationship is a hot topic in recent research [6]. Since phages insert their DNA into the bacterial host as part of their replication process, it is crucial to distinguish the phage-derived sequences from the bacterial sequences in any genome found in nature. The detection of phage within the host genome may have a significant impact on studying and understanding such viruses, however, the task is time-consuming and needs extensive lab work [7]. One possible approach to overcome such problems is to analyze metagenomic data that embed virus information as it is shorter than the full genome, ranging from 600 to 25K base pairs (bp) [8], which requires less time and effort. Metagenomics is the study of metagenomes from various environmental samples [8]. It is divided into two main areas: structural and functional metagenomics. Structural metagenomics primarily focuses on gene structure, whereas functional metagenomics examines the functions of genes, specifically the proteins they encode [9]. To classify the origin of a genome found in the environment, sequencing methods can be used. Earlier sequencing methods were based on gene similarity. In other words, it is required for a found genome to be similar to the known virus genome to classify it as an organism of that virus. This works by comparing the found genes to already known genes by creating an alignment. Examples of tools built based on similarity method are Kraken2 [10], Centrifuge [11], and FALCON-meta [12]. However, this method suffers from different shortcomings such as its low ability to discover new viruses since there is no universal viral marker gene exists at the moment [13].

Another proposed method is called binning, which is implemented in tools like MetaWatt [14] and CONCOT [15]. Binning introduces an additional step after the assembly method which groups the given contig into categories that correspond to a biological taxon—a classification used to denote a grouping of organisms, which can range from a single species to broader categories like genus or family, reflecting various levels of the biological hierarchy [8]. In addition, classical sequence comparison approaches such as BLAST [16, 17], has been employed (for a discussion, see [8]).

In addition to this algorithmic approaches, researchers have also investigated the use machine learning (ML) approaches for classifying metagenomic sequences, due to the success of ML in various bioinformatics applications. J. Ren et al [18], developed VirFinder, a logistic regression model to identify viral sequences given the genome. The proposed method can identify viruses within sequences of varying lengths, ranging from 500 to 10K bp. Testing VirFinder over NCBI data, however, it was found that VirFinder performs better on larger sequence lengths, which is often unrealistic. Expanding on this work, a new method called DeepVirFinder was introduced [19], serving the same objective as VirFinder. This deep learning approach uses a convolutional neural network (CNN) to identify viral sequences within DNA viral sequences. Unlike the original VirFinder algorithm, DeepVirFinder has been improved to identify viral sequences with shorter lengths, specifically between 150 and 3000 bp. Additionally, this method is capable of identifying viral sequences in real human gut metagenomic samples.

There are several other deep-learning based models employing different neuronal architectures for metagenomics based task. Thus, Seeker [20] solves the problem of differentiating phage sequences from bacterial ones and is based on long-short-term memory (LSTM) architecture. F. Liu et al [21] proposed RNN-VirSeeker, a deep-learning method for viral sequence identification also utilizing LSTMs and outperforms other state-of-the-art methods. PPR-META [22] is a deep learning based method that classifies contigs into phage, plasmid, and chromosome categories. The method was tested on artificial and real genomes of different lengths, ranging from 100 to 10,000 bp, and successfully outperformed other state-of-art methods. Finally, VIDHOP [23] is a method that can identify the original host of the virus in addition to the potential host genome. Two different deep learning methods were proposed the first one was based on LSTM, while the other one combines LSTM with CNN.

Despite these advances, previous state-of-the-art methods often underperform, particularly in identifying new genomes, as we demonstrate in our benchmark based on a viral metagenomic dataset from infant guts. Additionally, authors fail to provide a method for cross-verifying classifications based on DNA sequences with those based on proteins derived from these DNA sequences. Our tool outperforms previous approaches in the mentioned benchmark and also introduces a method to classify protein sequences, which vastly outperforms hidden markov model (HMM) based methods.

This study proposes a deep learning-based method, HVSeeker, to enhance the identification of host and viral sequences in metagenomic data. We developed a robust approach for HVSeeker by testing different preprocessing methods and validating the performance across different degrees of homology. Combined with models architectures particular sensitive to sequence data, this allows us to create an approach that outperforms previous models. Experimental results demonstrate that HVSeeker can accurately identify both short and long host and viral sequences in metagenomes, outperforming a variety of widely used methods: Seeker, Rnn-VirSeeker, DeepVirFinder, and PPR-Meta.

Methods

Data Collection & Description

The research data for this study was gathered from well-known bioinformatics databases, including the National Center for Biotechnology Information (NCBI) [24] and the Integrated Microbial Genomes & Microbiomes - Viruses (IMGVR) [25, 26]. The data consists of Bacterial and Phage DNA sequences. Each file contains a complete genome of different lengths and some metadata, e.g. the unique ID for each sequence and its length. The full dataset comprises 536 and 2687 bacterial and phage sequences, respectively.

Data Preprocessing

The initial dataset consists of various DNA sequences encapsulated within Fasta files. These sequences vary in both biological origins and lengths. A preliminary task in our methodology involves the extraction of proteins from these diverse DNA sequences, which correspond to multiple classes. Thus, the input data for the subsequent training models constitute both DNA and protein sequences.

To ensure consistency in our data processing, we adjust the length of each DNA sequence to a uniform standard. In this process, we split the entire genome into segments of 1000 base pairs (bp), a procedure that often results in sequences shorter than the designated length as the genomes are usually not a multiple of our subsequence length (i.e. 1000bp).

To address this issue, we employ three distinct strategies. The first strategy involves sequence padding, wherein we repetitively cycle through the sequence until it attains the required length. When padding sequences that are naturally shorter than the maximum number of base pairs, they are extended by duplicating their own sequence, therefore completing the short sequence by repeating it in the same order. The second strategy, which we termed contigs assembly approach, is a two-fold process: we initially combine multiple shorter left-over sequences to generate a new, longer sequence and split it into subsequences of our chosen length. Subsequently we apply padding to any residual segment that hasn't been incorporated into another sequence. The last strategy is a sliding-window process. In other words, for each sequence, we will select the first 1000 bp, and continue the processes by moving the window by 100bp until the end of the DNA. These techniques allow us to process input data of arbitrary length.

Upon achieving DNA sequences of uniform length, we proceed to eliminate any exact duplicate sequences.

Subsequently, we employ one-hot encoding to transform the contigs from their original nucleotide form (adenine, cytosine, guanine, thymine, or ACGT) to a binary matrix of 0s and 1s. This conversion facilitates easier processing and interpretation of the genetic data by our computational model.

In the final preprocessing step, we adopt an undersampling approach to balance the classes in our dataset. This procedure ensures that our model does not exhibit bias towards any particular class, leading to more reliable and generalizable predictions. With these steps, we conclude the preprocessing of the genomic sequence data. Following preprocessing, the dataset increased to 565,760 DNA sequences. For the model training process, we employed the holdout method, a recommended approach for larger datasets. The allocation of the data was set at 80% for the training set, with the remaining 20% equally split between validation and testing, at 10% each. To prevent overfitting, we employed an early-stopping technique, which terminates the training when the performance on the validation set degrades. The below points provide a summary of the preprocessing methodology adopted in this research. Additionally, a visual summary of the same can be found in Figures ??, ??, and ??.

Our model for classifying proteins, based on their amino acid sequences, uses a dataset comprising 98720 unique phage sequences and 122366 unique bacterial sequences. To ensure a broad representation of protein diversity, we employ BLAST to limit sequence homology between sets. This approach allows us to effectively capture the wide range of diversity found in bacterial and phage proteins. For testing purposes, we divided the data into 80% training data and 20% test data and repeated this process 5 times.

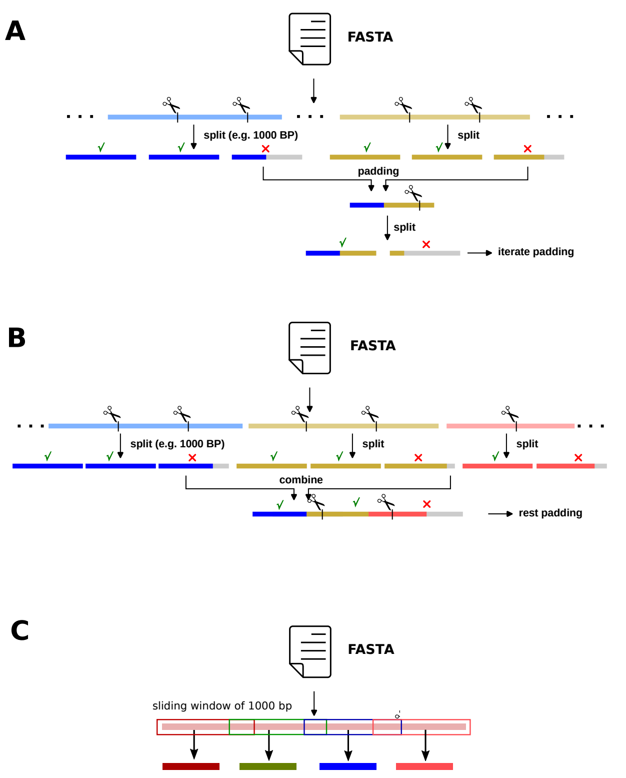

Figure 1. A) Data preprocessing with padding. We cycle through the sequence until it attains the required length. B) Data preprocessing with contigs assembly method. Initially, we combine multiple shorter sequences to generate a longer sequence, which is then split again into subsequences of length 1000bp. Finally, we apply padding to any residual sequence. C) Data preprocessing with 1000/100 bp sliding window method. We use a window of 1000 bp size to slide over the input DNA sequence in 100 bp steps.

These steps encapsulate the preprocessing stage of our research which will create two datasets, one for HVSeeker-DNA and another for HVSeeker-Protein:

- Extraction of proteins from DNA sequences.
- Standardization of DNA sequence lengths to 1000 bp.
- Handling of shorter sequences via padding, a combination of multiple sequences, or a sliding-window process.
- Removal of duplicate sequences.
- Transformation of nucleotide sequences to a binary matrix using one-hot encoding.
- Class balance in the dataset achieved through undersampling.

The proposed models

To leverage the availability of both DNA and protein sequences, we develop two distinguished models. The first model, called HVSeeker-DNA, is an LSTM-based model that takes as input DNA sequences with a length of 1000 bps. LSTMs are primarily designed for sequential data where each input influences subsequent or preceding entries. Similarly, in DNA sequences, each nucleotide is related to its context, providing distinct meaning compared to individual elements. Therefore, we consider LSTMs to be an optimal choice for addressing DNA-related challenges. For the second one,

HVSeeker-Protein, we adopt the ProteinBERT [27] as a pre-train model and perform a fine-tuning phase using the constructed protein sequences. ProteinBERT has been trained on diverse protein sequence corpus in a self-supervised manner. Therefore, it allows to have a generic representation for every input protein sequence. In the following, each model will be described in detail.

HVSeeker-DNA structure

HVSeeker-DNA consists of three bidirectional connected LSTM units, followed by two fully connected layers, and then a softmax activation function for the final prediction. The first LSTM unit reads each data entry as a 6×1000 matrix due to the one-hot encoding process, then it will output a vector of length 150 to be passed to the next two LSTM models. Then, a fully connected layer with the elu activation function and dropout of 0.2 will read the output of the last LSTM model and pass it to the next fully connected layer before using the softmax activation function for the final prediction. A visual summary of the model is presented in Figure 2

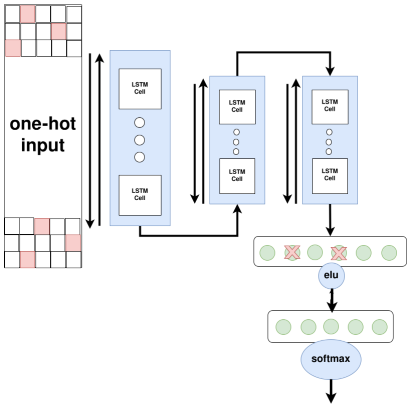

Figure 2. HVSeeker-DNA Architecture. First, the DNA data is encoded using different principles (padding, contigs-assembly, sliding window). We then process the one-hot encoded DNA using 3 bidirectional LSTM layers, followed by a linear layer with a moderate amount of dropout (0.2) and an elu activation function, and an output layer.

HVSeeker-Protein structure

In the event that the classification provided by the first model requires support with additional information about the proteins expressed by the DNA, we offer a second model that relies solely on the expressed protein sequences. To classify proteins as either phage or bacterial, we utilize embeddings generated by ProteinBERT. These embeddings accurately represent the input proteins, thanks to the extensive dataset of training proteins used in ProteinBERT [27]. The model's architecture includes a transformer with four attention heads and six layers, featuring a key size of 64. It has been trained on a large dataset of 160 million protein sequences, focusing on a reconstruction task. To enhance the finetuned model's precision in predicting protein types, we employ a Bayesian optimizer. This optimizer uses an expected improvement acquisition function, initiating with eight random starts and conducting 25 evaluations on 1000 proteins sampled from the training set. These evaluations fine-tune the learning rate, the number of training epochs, and the learning rate decay factor, optimizing the model's performance.

To evaluate the effectiveness of the optimization and fine-tuning process, we conduct an assessment using 5-fold cross-validation for the optimization process for five runs. For each test set, we use BLAST to ensure that sequence homology between the training and test set does not exceed 0.95. By employing this method, we can rigorously test the model across different subsets of the data, guaranteeing that our assessment of its performance is both fair and reliable.

Evaluation Criteria

To evaluate the effectiveness of the proposed models we focus on four key metrics: Accuracy, Precision, Recall, and F-1 score.

To evaluate model performance accuracy

$$Accuracy = \frac{TP + TN}{TP + FP + TN + FN} \quad (1)$$

is commonly used to calculate the ratio of correctly predicted instances to the total number of predictions.

However, accuracy can be misleading in the case of imbalanced datasets. Therefore, relying solely on accuracy is insufficient, and other metrics must be considered.

When wanting to focus on the positive class, precision

$$Precision = \frac{TP}{TP + FP} \quad (2)$$

can be used to measures the proportion of correctly predicted positive instances relative to all predicted positives.

Measuring the proportion of correctly predicted positive instances out of actual positives, recall

$$Recall = \frac{TP}{TP + FN} \quad (3)$$

is a crucial metric for addressing the impact of model bias in imbalanced datasets.

To balance precision and recall, the F1 measure

$$F1\ measure = 2 \times \frac{Precision \times Recall}{Precision + Recall} \quad (4)$$

provides a robust metric, offering a more comprehensive view of a model's predictive strength.

Results & Discussion

To assess the effectiveness of the algorithm, four experiments were designed and executed. In the first experiment, we trained the model across a variety of sequence lengths to determine the optimal length for Bacteriophage prediction. In the second experiment, we performed a self-comparison of our model under three distinct preprocessing conditions to understand the impact of these variations on the model's performance. The third experiment compared our model with other models reported in the literature, on unseen data sequences from the same environment as our training set. The last experiment involved benchmarking our model against others in the literature using a standardized dataset. All the experiments are further discussed in the following sections.

Optimizing Sequence Length for Improved Prediction Performance

To find the best sequence length for Bacteriophage prediction and to assess model performance across different sequence lengths, we trained our model using sequences of 2000, 1500, 1000, 500, 200, and 100 base pairs. We used padding as a preprocessing method for shorter sequences to ensure consistent input lengths. Figure 3 displays a comparative analysis of model performance, presenting Precision, Recall, Accuracy, and F1-score for each evaluated sequence length. Figure 3 shows that the model trained on 2000

bp sequences consistently predicted the bacteria class, indicating potential overfitting and an inability to generalize. This could be due to the longer sequence lengths introducing noise. On the other hand, the model trained on 1000 bp sequences showed the best performance in terms of F1-score, outperforming other models with its Recall rate, despite the 1500 bp model achieving slightly higher Precision. Conversely, the model with 100 bp sequences underperformed, as expected due to the reduced informational content of shorter sequences for Bacteriophage identification. The findings also indicate that sequence lengths between 1500-2000 bp resulted in similar accuracy levels. This suggests that there is a threshold beyond which shorter sequence lengths start to noticeably affect model performance. Based on these results, subsequent experiments focused solely on the 1000 bp sequence length, which demonstrated the most balanced performance across all evaluated metrics.

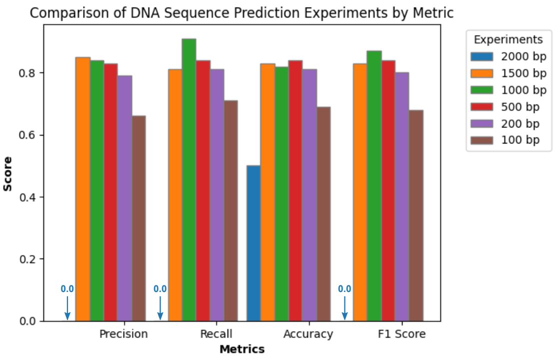

Figure 3. Comparison on different sequence lengths in terms of Precision, Recall, F1-score, and Accuracy. We see the best performance in terms of the F1-score when using a length of 1000 bp and the best performance in terms of recall for a length of 1500 bp. Length 2000 bp achieved a score 0 for all metrics except for accuracy due to overfitting. Here, HVSeeker potentially fails to capture local sequence properties as effectively as with smaller sequence sizes. Overall, we can conclude that excessively large sequence lengths overfit, while overly small sequence lengths underperformed.

Evaluating the Impact of Preprocessing Techniques on Model Accuracy

In section , we introduce three preprocessing methods of the data: padding method, assembly of shorter sequences method, and sliding window method. To assess the impact of each preprocessing technique, we trained a separate model for each method. The training and validation accuracies of these models are shown in Figures 4, 5 respectively. According to these figures, the sliding window method initially led to higher accuracy during the early training epochs. However, as training progressed, all three models converged to comparable levels of accuracy for both the training and validation phases. Despite the overall similarity in performance, a closer inspection reveals that the padding method slightly outperformed the others in terms of validation accuracy, whereas the assembly of contigs method was slightly behind. This could be attributed to the padding method's tendency to duplicate nucleotides, potentially providing the model with more consistent training data. In contrast, the assembly method, which might combine sequences from varied origins, could introduce a higher degree of variability and confusion to the model's learning process. Eventually, the three models achieved validation accuracy exceeding 80% which indicates a robust model architecture capable of adapting to various preprocessing strategies. Another interesting observation is that all three methods achieved similar scores across all metrics, unlike others that may be biased towards recall or precision, like DeepVirFinder or PPR-Meta. This consistency could be attributed to the balanced dataset used for training HVSeeker, highlighting the importance of the undersampling step in preprocessing. Additionally, this demonstrates the robustness of HVSeeker's preprocessing and architecture, achieving an excellent balance between generalization and adapting to the training data.

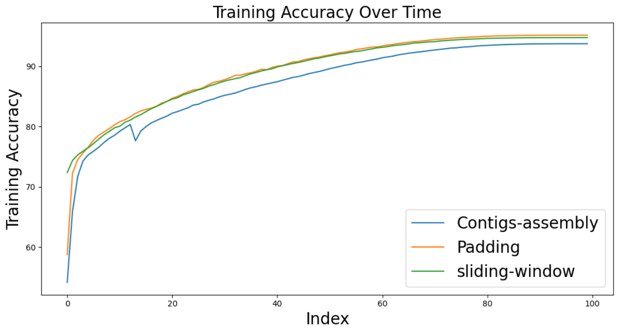

Figure 4. Training accuracy of the three models. All models converge relatively quickly to their maximal performance with small differences between the accuracies of the embedding methods.

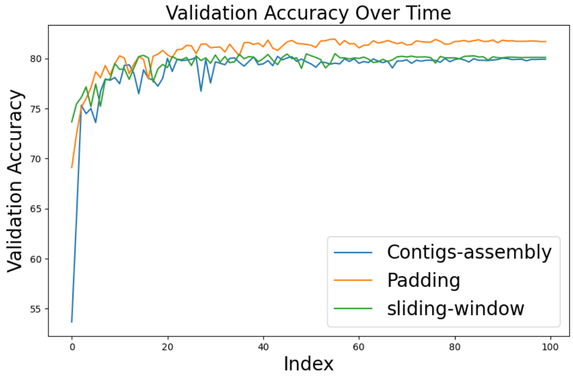

Figure 5. Validation accuracy of the three models. All models converge relatively quickly to their maximal performance with small differences between the accuracies of the embedding methods.

Benchmarking the Proposed Method Against Existing Tools

To extend our evaluation of the proposed models, we compared them with existing bacteriophage classification approaches found in the literature, specifically Seeker [20], Rnn-VirSeeker [21], DeepVirFinder [19], and PPR-Meta [22]. For a fair comparison, we ensured that each algorithm was trained using the same dataset introduced in our study. Table 1 outlines the final results of each model on unseen testing data. As shown in the table, the three proposed models outperformed both Seeker and Rnn-VirSeeker in terms of Precision, Recall, Accuracy, and F1 Score. While both DeepVirFinder and PPR-Meta achieved higher precision score, they achieved lower Recall, Accuracy, and F1 score. Notably, all the models posted comparable results across various evaluation metrics, suggesting that our model construction was robust enough to mitigate potential biases. Conversely, Seeker's model yielded an F1 score of approximately 58%, hinting at a need for more extensive data to enhance its performance. In contrast, Rnn-VirSeeker appeared to struggle with underfitting, being unable to adapt to the complexity of the training data, it only achieved an accuracy of roughly 50% on the

training data. This underperformance could stem from the label encoding technique RNN-Seeker employed for encoding their data, which assigns numerical values to nucleotides. This method is generally not recommended because it can prioritize some features over others and suggests an order between nucleotides, generally not found in the natural world, which potentially skews the data analysis and interpretation. In the case of DeepVirFinder and PPR-Meta, these models exhibit high precision but low recall. This indicates that while they are correctly identifying phages, many actual phage genomes remain undetected, thus lacking comprehensive accuracy and F1 score in phage prediction.

	Precision	Recall	Accuracy	F1-Score
Padding	83.72%	**90.07%**	**87.25%**	**86.78%**
Contigs-assembly	80.76%	80.02%	80.30%	80.39%
Sliding-window	83.40%	82.43%	82.81%	82.91%
Seeker	56.98%	58.95%	59.39%	57.95%
Rnn-VirSeeker	00.00%	00.00%	50.00%	00.00%
DeepVirFinder	90.50%	65.14%	79.23%	75.75%
PPR-Meta	**92.00%**	67.00%	81.00%	77.00%

Table 1. Comparison of the proposed approach with different preprocessing methods against Seeker, Rnn-seeker DeepVirFinder, and PPR-Meta. We find that our method outperforms others in most of the used metrics, specifically in Accuracy and F1 score, regardless of the input embedding used. Both using padding and a sliding-window approach result in similar good results, with both methods showing a slight advantage over using contigs-assembly.

Performance on low-homology datasets

To challenge the generalization ability of our model, we use our previously created test set and remove all sequences above a certain homology cutoff by using BLAST to compare the sequences to our training set. Based on the resulting alignment files, we create five different homology datasets. The results for all datasets can be found in supplementary figure S3-S7. HVSeeker consistently achieves the best F1-Score for all data splits. When using a homology cutoff of 60%, HVSeeker still achieves an F1-Score of 68.60% with a precision of 57.70% and a recall of 84.80%. Other methods usually do not balance precision and recall as well, resulting in a considerably lower F1-Score.

	Precision	Recall	Accuracy	F1-Score
HVSeeker	57.70%	84.80%	70.30%	**68.60%**
Seeker	38.29%	**98.51%**	38.57%	55.15%
Rnn-VirSeeker	00.00%	00.00%	62.00%	00.00%
DeepVirFinder	63.33%	51.93%	70.03%	57.06%
PPR-Meta	**78.00%**	44.00%	**74.00%**	56.00%

Table 2. Comparsion of the proposed approach with previous methods against Seeker, Rnn-seeker DeepVirFinder, and PPR-Meta on a low-homology dataset of 60% maximum homology. We find HVSeeker achieves the highest F1-Score with 68.60%, roughly 10% better than the second best method DeepVirFinder with 57.06%. Additionally used low-homology sets can also be found in the supplementary under Table S3-S7.

Performance on a diverse viral metagenomic dataset from infant guts metagenomic dataset

A key challenge in bacteriophage detection is the recognition of new sequence patterns. To evaluate our algorithm's capability in this regard, we conducted a performance comparison using benchmark datasets collected from [28] with Seeker, Rnn-VirSeeker, DeepVirFinder, and PPR-Meta. The study analyzed viral diversity in the fecal viromes of 647 one-year-olds from the Copenhagen Prospective Studies on Asthma in Childhood 2010 (COPSAC2010). Fecal samples were successfully collected and viromes were characterized for 647 children at one year, with metagenomes sequenced in parallel. The study's authors identified 10,000 viral species from 248 virus family-level clades, with 232 being newly discovered, primarily from the Caudoviricetes class. Hosts for 79% of the phages were determined using CRISPR spacers from bacterial metagenomes of the same children. The results of our comparison are detailed in Table 3. The proposed algorithm consistently outperformed all methods across all evaluated metrics except accuracy. Notably, our algorithm demonstrated a greater recall relative to precision, which can be attributed to the benchmark dataset's composition—featuring a higher count of negative than positive instances. With a final F1 Score of 0.767, closely mirroring its performance on testing data, our model proves its proficiency in accurately processing sequences from varied environments, comparably to how it performs with the training set's environment. Conversely, Seeker, with an F1 Score of 0.578, DeepVirFinder with an F1 score of 0.417, and PPR-Meta with 44, adapt to the data during training only to a limited extent, suggesting either insufficient data or inadequate model complexity. Meanwhile, Rnn-VirSeeker exhibited underfitting, indicating a failure to learn effectively from the training data.

	Precision	Recall	Accuracy	F1-Score
HVSeeker	**67.01%**	**89.74%**	65.23%	**76.73%**
Seeker	42.92%	88.43%	46.40%	57.79%
Rnn-VirSeeker	00.00%	00.00%	14.46%	00.00%
DeepVirFinder	32.18%	59.04%	74.22%	41.66%
PPR-Meta	34.00%	61.00%	**77.00%**	44.00%

Table 3. Comparison on benchmark data. Again HVSeeker outperforms the alternative method Seeker by an impressive margin of 18.9 for the F1-Score and 18.83 in terms of the classification accuracy. Meanwhile, Rnn-VirSeeker seems to be unable to learn properly from the provided data.

Protein based classification

We report the average performance over all five runs of our method assessed on our test-sets, achieving an average AUC value of 0.89. This indicates a strong performance in the protein-based classification task. Additionally, the classification accuracy across all splits is 82%, further demonstrating the method's effectiveness. The weighted F1-score stands at 0.82, with a weighted precision of 0.82 and a weighted recall of 0.82. The additional information created by this classification mechanism can be used reliably to support the prediction created purely based on the DNA. For the minority of the sequences of a length from 571 to 1144 we find a similar AUC value of 0.88, indicating the stability of the results for greater sequence lengths. The weighted F1-score stands at 0.84, with a weighted precision of 0.84 and a weighted recall of 0.84. To compare our method with a baseline approach, we constructed Hidden Markov Models (HMMs) for all five subsets of the training dataset. Initially, we generated multiple sequence alignments using MAFFT (FFT-NS-2) [29], followed by the construction of HMMs with HMMbuild [30]. The subsequent search over the test data yielded only 12 correct hits above the standard reporting threshold. This result indicates the model's inability to adequately represent the diversity of the input data, translating to an accuracy of approximately 0.0051%.

Conclusion

In this study, we introduced HVSeeker, a novel methodology designed to differentiate between viral and bacterial genomic sequences. HVSeeker surpasses the existing state-of-the-art methods, DeepVirFinder, PPR-Meta, RNN-VirSeeker, and Seeker, in performance on two benchmarks and additionally evaluates corresponding proteins, offering improved insights. Unlike previous methods HVSeeker can work with DNA input as well as protein inputs, allowing researchers to combine evidence for both. This advancement is important for accurately identifying viral genomes and therefore the creation of new therapeutic approaches such as phage therapy. Identifying the host and viral sequences in mixed metagenomes is the initial step toward analyzing the host viral component of samples. This process is crucial for downstream work. HVseeker proves to be an essential tool in prokaryotic and phage taxonomy, as well as in bacterial-host interactions.

Availability of source code and requirements

- Project name: HVSeeker [31]
- Project home page: https://github.com/BackofenLab/HVSeeker
- Programming language: Python
- Other requirements: To install software requirements we provide a conda environment file for HVSeeker-DNA and HVSeeker-Protein in the github repo
- License: MIT licence
- in bio.tools registered as hvseeker
- rrid: SCR_026120
- An archival copy of the github repository is available via software heritage [32]
- see DOME-ML questionary for more details [33]

Data availability

The data belonging to the study can be found in the *Giga*DB of the publication. The used model is available under https://github.com/BackofenLab/HVSeeker.

Additional Files

- Supplementary table S1: Comperative performance metrics of sequence identification methods on test set
- Supplementary table S2: Performance metrics of HVSeeker, Seeker, and Rnn-VirSeeker on unseen benchmark dataset
- Supplementary table S3: Comparative performance metrics of sequence identification methods on test dataset with a maximum of 95% homology
- Supplementary table S4: Comparative performance metrics of sequence identification methods on test dataset with a maximum of 90% homology
- Supplementary table S5: Comparative performance metrics of sequence identification methods on test dataset with a maximum of 80% homology
- Supplementary table S6: Comparative performance metrics of sequence identification methods on test dataset with a maximum of 70% homology
- Supplementary table S7: Comparative performance metrics of sequence identification methods on test dataset with a maximum of 60% homology

Declarations

List of abbreviations

- LSTM: Long-short-term-memory.
- bp: base-pairs
- ML: machine-learning
- Pfam: protein family
- CNN: convolutional neural network
- HMM: hidden markov model
- NCBI: National Center for Biotechnology Information
- IMGVR: Integrated Microbial Genomes & Microbiomes - Viruses

Consent for publication

Not applicable

Competing Interests

The author(s) declare that they have no competing interests.

Funding

Deutsche Forschungsgemeinschaft Grant, BA 2168/23, Much more than Defence: the Multiple Functions and Facets of CRISPR–Cas. And Deutsche Forschungsgemeinschaft, Grant, BA 2168/25-1, Einluss von RNA-bindenden Proteinen und mRNA-Strukturen auf alternative Translation-Regulationsmechanismen im entzündlichen Tumorgeschehen. The article processing charge is funded by the Baden-Wuerttemberg Ministry of Science, Research and Art and the University of Freiburg in the funding programme Open Access Publishing

Author's Contributions

Al-Najim and S.H. developed the software. Al-Najim, S.H. and O.S.A wrote the initial draft of the manuscript. O.S.A. conceived the study and analyses. O.S.A and V.D.T. data acquisition. O.S.A., and R.B. oversaw the project. All authors reviewed, contributed to, and approved the manuscript.

Acknowledgements

The authors acknowledge support by the High Performance and Cloud Computing Group at the Zentrum für Datenverarbeitung of the University of Tübingen, the state of Baden-Württemberg through bwHPC and the German Research Foundation (DFG) through grant no INST 37/935-1 FUGG. The authors also would like to thank King Fahd University of Petroleum and Minerals and thank the reviewers for their valuable suggestions.

References

1. Paez-Espino D, Eloe-Fadrosh EA, Pavlopoulos GA, Thomas AD, Huntemann M, Mikhailova N, et al. Uncovering Earth's virome. Nature 2016;536:425–430.
2. Woolhouse M, Gaunt E. Ecological origins of novel human pathogens. Critical Reviews in Microbiology 2007;33:231–242.
3. Zhang Z, Cai Z, Tan Z, Lu C, Jiang T, Zhang G, et al. Rapid identification of human-infecting viruses. Transboundary and Emerging Diseases 2019;66(6):2517–2522.
4. Elst NV, Meyer E. Potential therapeutic application of bacteriophages and phage-derived endolysins as alternative treatment of bovine mastitis. Vlaams Diergeneeskundig Tijdschrift 2018;87(4):181–187.

5. Stone E, Campbell K, Grant I, McAuliffe O. Understanding and exploiting phage–host interactions. Viruses 2019;11(6):567.
6. Weber-Dabrowska B, Jonczyk-Matysiak E, zaczek M, Lobocka M, Lusiak-Szelachowska M, Gorski A. Bacteriophage procurement for therapeutic purposes. Frontiers in Microbiology 2016;7.
7. Brown TA. Genomes. 2 ed. Bios Scientific Publishers; 2002.
8. Tonkovic P, Kalajdziski S, Zdravevski E, Lameski P, Corizzo R, Pires IM, et al. Literature on applied machine learning in metagenomic classification: A scoping review. Biology 2020;9(12):453.
9. Alves Ld, Westmann CA, Lovate GL, de Siqueira GM, Borelli TC, Guazzaroni ME. Metagenomic approaches for understanding new concepts in Microbial Science. International Journal of Genomics 2018;2018:1–15.
10. Wood DE, Lu J, Langmead B. Improved metagenomic analysis with Kraken 2. Genome Biology 2019;20:257.
11. Kim D, Song L, Breitwieser FP, Salzberg SL. Centrifuge: rapid and sensitive classification of metagenomic sequences. Genome Research 2016;26:1721–1729.
12. Pratas D, Hosseini M, Grilo G, Pinho AJ, Silva RM, Caetano T, et al. Metagenomic Composition Analysis of an Ancient Sequenced Polar Bear Jawbone from Svalbard. Genes 2018;9(9):445.
13. Pappas N, Roux S, Hölzer M, Lamkiewicz K, Mock F, Marz M, et al. Virus bioinformatics. Encyclopedia of Virology 2021;p. 124–132.
14. Strous M, Kraft B, Bisdorf R, Tegetmeyer HE. The binning of metagenomic contigs for microbial physiology of mixed cultures. Frontiers in Microbiology 2012;3.
15. Alneberg J, Bjarnason BS, de Bruijn I, Schirmer M, Quick J, Ijaz UZ, et al. Binning metagenomic contigs by coverage and composition. Nature Methods 2014;11(11):1144–1146.
16. Khandelwal I, Sharma A, Agrawal PK, Shrivastava R. Bioinformatics database resources. Library and Information Services for Bioinformatics Education and Research 2017;p. 45–90.
17. Raza K. Application of Data Mining in Bioinformatics. Journal of Computer Science and Engineering 2012;.
18. Ren J, Ahlgren NA, Lu YY, Fuhrman JA, Sun F. Virfinder: A novel K-mer based tool for identifying viral sequences from assembled metagenomic data. Microbiome 2017;5(1).
19. Ren J, Song K, Deng C, Ahlgren NA, Fuhrman JA, Li Y, et al. Identifying viruses from metagenomic data using deep learning. Quantitative Biology 2020;8(1):64–77.
20. Auslander N, Gussow AB, Benler S, Wolf YI, Koonin EV. Seeker: Alignment-free identification of bacteriophage genomes by Deep Learning. Nucleic Acids Research 2020;48(21).
21. Liu F, Miao Y, Liu Y, Hou T. RNN-VirSeeker: A deep learning method for identification of short viral sequences from metagenomes. IEEE/ACM Transactions on Computational Biology and Bioinformatics 2020;p. 1–1.
22. Fang Z, Tan J, Wu S, Li M, Xu C, Xie Z, et al. PPR-Meta: A tool for identifying phages and plasmids from metagenomic fragments using deep learning. GigaScience 2019;8(6).
23. Mock F, Viehweger A, Barth E, Marz M. VIDHOP, viral host prediction with Deep Learning. Bioinformatics 2020;37(3):318–325.
24. National Center for Biotechnology Information (NCBI), NCBI Database; 2023. https://www.ncbi.nlm.nih.gov, accessed: 2023-06-27.
25. Grigoriev IV, Nordberg H, Shabalov I, Aerts A, Cantor M, Goodstein D, et al. The Genome Portal of the Department of Energy Joint Genome Institute. Nucleic acids research 2012;40(D1):D26–D32.
26. Nordberg H, Cantor M, Dusheyko S, Hua S, Poliakov A, Shabalov I, et al. The genome portal of the Department of Energy Joint Genome Institute: 2014 updates. Nucleic acids research 2014;42(D1):D26–D31. PMID: 24225321; PMCID: PMC3965075.
27. Brandes N, Ofer D, Peleg Y, Rappoport N, Linial M. ProteinBERT: a universal deep-learning model of protein sequence and function. Bioinformatics 2022;38:2102–2110.
28. Shah SA, Deng L, Thorsen J, Pedersen AG, Dion MB, Castro-Mejía JL, et al. Expanding known viral diversity in the healthy infant gut. Nature Microbiology 2023;8(5):986–998. https://doi.org/10.1038/s41564-023-01345-7.
29. Katoh K, Standley DM. MAFFT multiple sequence alignment software version 7: improvements in performance and usability. Mol Biol Evol 2013;30(4):772–80. PMID: 23329690; PMCID: PMC3603318.
30. Eddy SR. Accelerated profile HMM searches. PLOS Comp Biol 2011;.
31. HVSeeker (2024). HVSeeker (Version 1.0);, https://github.com/BackofenLab/HVSeeker.
32. Al-Najjim A, Hauns S, Dinh VT, Backofen R, Alkhnbashi O. HVSeeker: A Deep Learning-based Method for Identification of Host and Viral DNA sequences (Version 1) [Computer software]. Software Heritage; 2024, https://archive.softwareheritage.org/swh:1:snp:02520873e4566c63c913d962d9fd01d81410f348;origin=https://github.com/bulatef/HVSeeker.
33. DOME-ML Annotations for "HVSeeker: A Deep Learning-based Method for Identification of Host and Viral DNA sequences"; 2024, https://registry.dome-ml.org/review/igr5x3a1vs.

Click here to access/download
Supplementary Material
HVSeeke_Supplementary.pdf

 Click here to access/download;Personal Cover;Cover-letter.pdf

Dear Dr. Hans
Editor-in-Chief of GiGascience

Herewith we submit the revised version of our manuscript “HVSeeker: A Deep Learning-based Method for Identification of Host and Viral DNA sequences”.

We have answered each point and have modified our submission accordingly. We have also carefully considered the reviewer’s comments and integrated them into a thoroughly revised manuscript version.

We thank the reviewers for their supportive comments and constructive feedback and the editor for the opportunity to submit a revised manuscript. Our response to each comment below is in blue.

I am looking forward to hearing from you.

Sincerely yours,

Dr. Omer Alkhnbashi

Point-to-point response to reviewer comments

1) At GigaScience we treat github repos, online datasets and other internet resources as citable items. Please add the following items to the bibliography as separate, numbered references: (please refer to our instructions for authors for the correct format).

[xx] HVSeeker. Github repository. 2024. https://github.com/bulatef/HVSeeker
Author's response: Done

[xx] HVSeeker [Software Heritage]. 2024.
https://archive.softwareheritage.org/swh:1:snp:02520873e4566c63c913d962d9fd01d81410f348;origin=https://github.com/bulatef/HVSeeker
Author's response: Done

[xx] DOME-ML Annotations for "HVSeeker: A Deep Learning-based Method for Identification of Host and Viral DNA sequences". DOME Registry. 2024. https://registry.dome-ml.org/review/igr5x3a1vs

Author's response: Done

Please cite these resources by reference number from the data availability section.
(To cite specific data items in your github, such as the DNA and protein sample data, you may refer to sub-folders of the github repo in the text, without making a separate reference item for each sub-folder).
Author's response: Done

For the software heritage archive, please write something along the lines " An archival copy of the github repository is available via software heritage [xx]".
Author's response: Done

2) In addition, please register any new software application in the bio.tools and SciCrunch.org databases to receive RRID (Research Resource Identification Initiative ID) and biotoolsID identifiers, and include these in your manuscript (in the software availability section).
Author's response: Done

3) Please structure your abstract according to the instructions for Technical Notes ("Background - Results - Conclusions")
Author's response: Done

4) At this stage you may remove any highlighting that was made for the purpose of peer review.

Author’s response: Done

5) Please also ensure that your revised manuscript conforms to the journal style, which can be found in the Instructions for Authors on the journal homepage.

Author’s response: Done
