# Supplementary material for: HVSeeker: a deep-learning-based method for identification of host and viral DNA sequences
Source: Gigascience. 2025 May 15;14:giaf037. doi: 10.1093/gigascience/giaf037 (PMC12080225; doi:10.1093/gigascience/giaf037)
Supplement: giaf037_GIGA-D-24-00282_Revision_4 [file giaf037_giga-d-24-00282_revision_4.pdf]

GigaScience

HVSeeker: A Deep Learning-based Method for Identification of Host and Viral DNA sequences

--Manuscript Draft--

**Manuscript Number:**	GIGA-D-24-00282R4
**Full Title:**	HVSeeker: A Deep Learning-based Method for Identification of Host and Viral DNA sequences
**Article Type:**	Technical Note
**Funding Information:**	Deutsche Forschungsgemeinschaft ([BA 2168/23-1/2) — Prof Rolf Backofen
**Abstract:**	Motivation: Bacteriophages are among the most abundant organisms on Earth, significantly impacting ecosystems and human society. The identification of viral sequences, especially novel ones, from mixed metagenomes is a critical first step in analyzing the viral components of host samples. This plays a key role in many downstream tasks. However, this is a challenging task due to their rapid evolution rate. The identification process typically involves two steps: distinguishing viral sequences from the host and identifying if they come from novel viral genomes. Traditional metagenomic techniques that rely on sequence similarity with known entities often fall short, especially when dealing with short or novel genomes. Meanwhile, deep learning has demonstrated its efficacy across various domains, including the Bioinformatics field.<br>Results: We have developed HVSeeker, a deep learning-based method for distinguishing between bacterial and phage sequences. HVSeeker consists of two separate models: one analyzing DNA sequences and the other focusing on proteins. This method has shown promising results on sequences with various lengths, ranging from 200 to 1500 base pairs. Tested on both NCBI and IMGVR databases, HVSeeker outperformed several methods from the literature such as Seeker, Rnn-VirSeeker, DeepVirFinder, and PPR-Meta. Moreover, when compared with other methods on benchmark datasets, HVSeeker has shown better performance, establishing its effectiveness in identifying unknown phage genomes.<br>Availability: A Python implementation of HVseeker and the Python code developed for this study have been provided on Github https://github.com/bulatef/HVSeeker.
**Corresponding Author:**	Omer Alkhnbashi, PhD<br>MBRU: Mohammed Bin Rashid University of Medicine and Health Sciences<br>Dubai, UNITED ARAB EMIRATES
**Corresponding Author Secondary Information:**	
**Corresponding Author's Institution:**	MBRU: Mohammed Bin Rashid University of Medicine and Health Sciences
**Corresponding Author's Secondary Institution:**	
**First Author:**	Omer Alkhnbashi, PhD
**First Author Secondary Information:**	
**Order of Authors:**	Omer Alkhnbashi, PhD
	Abdullatif Al-Najim
	Sven Hauns
	Rolf Backofen
	Van Dinh Tran
**Order of Authors Secondary Information:**	
**Response to Reviewers:**	Dear Dr. Hans<br>Editor-in-Chief of GiGascience

Powered by Editorial Manager® and ProduXion Manager® from Aries Systems Corporation

	Herewith we submit the revised version of our manuscript “HVSeeker: A Deep Learning-based Method for Identification of Host and Viral DNA sequences”<br><br>We have carefully addressed all the comments and suggestions provided by the journal and integrated them into a thoroughly revised manuscript version.<br><br>Additionally, we would like to take this opportunity to thank you, the reviewers, and the editorial team for the acceptance of our paper. We greatly appreciate the constructive feedback and guidance throughout the review process, which has significantly contributed to improving our manuscript. Our response to each comment below is in blue.<br><br>I am looking forward to hearing from you.<br><br>Sincerely yours,<br>Dr. Omer Alkhnbashi<br><br>--<br><br>Point-to-point response to reviewer comments<br><br>- In the data availability section, you write "The data belonging to the study can be found in the GigaDB of the publication." - As far as I understood, our curators didn't feel it was necessary to prepare a GigaDB set, as the data and code are available form other sources (e.g. Software Heritage) - if that's the case, you can delete this sentence.<br><br>Author’s response: Done<br><br>- Also in the data availability section, you write "The used model is available under https://github.com/BackofenLab/HVSeeker" - please also cite the URL here, i.e " "The used model is available via the github repository [31]"<br><br>Author’s response: Done<br><br>- In the Data availability section, please also include the sentence "DOME-ML annotations are available vie the DOME registry under accession igr5x3a1vs [33]"<br><br>Author’s response: Done<br><br>- Importantly, at this stage, we need editable files (word or LaTex source code and bib/style files) - please upload them to EM in addition to an converted PDF.<br><br>Author’s response: Done
**Additional Information:**	
**Question**	**Response**
Are you submitting this manuscript to a special series or article collection?	No
**Experimental design and statistics**	Yes

Powered by Editorial Manager® and ProduXion Manager® from Aries Systems Corporation

Full details of the experimental design and statistical methods used should be given in the Methods section, as detailed in our Minimum Standards Reporting Checklist. Information essential to interpreting the data presented should be made available in the figure legends.<br><br>Have you included all the information requested in your manuscript?	
**Resources**<br><br>A description of all resources used, including antibodies, cell lines, animals and software tools, with enough information to allow them to be uniquely identified, should be included in the Methods section. Authors are strongly encouraged to cite Research Resource Identifiers (RRIDs) for antibodies, model organisms and tools, where possible.<br><br>Have you included the information requested as detailed in our Minimum Standards Reporting Checklist?	Yes
**Availability of data and materials**<br><br>All datasets and code on which the conclusions of the paper rely must be either included in your submission or deposited in publicly available repositories (where available and ethically appropriate), referencing such data using a unique identifier in the references and in the "Availability of Data and Materials" section of your manuscript.<br><br>Have you have met the above requirement as detailed in our Minimum Standards Reporting Checklist?	Yes

LaTeX - Main Document (TeX file)
Click here to access/download;LaTeX - Main Document (TeX file);HVSeeke-Manuscript.tex

```
This is pdfTeX, Version 3.141592653-2.6-1.40.26 (TeX Live 2024)
(preloaded format=pdflatex 2024.8.2)  3 FEB 2025 01:17
entering extended mode
 restricted \write18 enabled.
 %&-line parsing enabled.
**hvseeke-manuscript.tex
(./HVSeeke-Manuscript.tex
LaTeX2e <2024-06-01> patch level 2
L3 programming layer <2024-05-27>
(./oup-contemporary.cls
Document Class: oup-contemporary 2023/06/12, v1.2
(c:/texlive/2024/texmf-dist/tex/latex/base/article.cls
Document Class: article 2024/02/08 v1.4n Standard LaTeX document class
(c:/texlive/2024/texmf-dist/tex/latex/base/size10.clo
File: size10.clo 2024/02/08 v1.4n Standard LaTeX file (size option)
)
\c@part=\count194
\c@section=\count195
\c@subsection=\count196
\c@subsubsection=\count197
\c@paragraph=\count198
\c@subparagraph=\count199
\c@figure=\count266
\c@table=\count267
\abovecaptionskip=\skip49
\belowcaptionskip=\skip50
\bibindent=\dimen141
) (c:/texlive/2024/texmf-dist/tex/latex/base/inputenc.sty
Package: inputenc 2024/02/08 v1.3d Input encoding file
\inpenc@prehook=\toks17
\inpenc@posthook=\toks18
) (c:/texlive/2024/texmf-dist/tex/latex/base/fontenc.sty
Package: fontenc 2021/04/29 v2.0v Standard LaTeX package
) (c:/texlive/2024/texmf-dist/tex/generic/iftex/ifpdf.sty
Package: ifpdf 2019/10/25 v3.4 ifpdf legacy package. Use iftex instead.
(c:/texlive/2024/texmf-dist/tex/generic/iftex/iftex.sty
Package: iftex 2022/02/03 v1.0f TeX engine tests
)) (c:/texlive/2024/texmf-dist/tex/latex/microtype/microtype.sty
Package: microtype 2024/03/29 v3.1b Micro-typographical refinements (RS)
(c:/texlive/2024/texmf-dist/tex/latex/graphics/keyval.sty
Package: keyval 2022/05/29 v1.15 key=value parser (DPC)
\KV@toks@=\toks19
) (c:/texlive/2024/texmf-dist/tex/latex/etoolbox/etoolbox.sty
Package: etoolbox 2020/10/05 v2.5k e-TeX tools for LaTeX (JAW)
\etb@tempcnta=\count268
)
\MT@toks=\toks20
\MT@tempbox=\box52
\MT@count=\count269
LaTeX Info: Redefining \noprotrusionifhmode on input line 1061.
LaTeX Info: Redefining \leftprotrusion on input line 1062.
\MT@prot@toks=\toks21
LaTeX Info: Redefining \rightprotrusion on input line 1081.
LaTeX Info: Redefining \textls on input line 1392.
```

```
\MT@outer@kern=\dimen142
LaTeX Info: Redefining \textmicrotypecontext on input line 2013.
\MT@listname@count=\count270
(c:/texlive/2024/texmf-dist/tex/latex/microtype/microtype-pdftex.def
File: microtype-pdftex.def 2024/03/29 v3.1b Definitions specific to
pdftex (RS)

LaTeX Info: Redefining \lsstyle on input line 902.
LaTeX Info: Redefining \lslig on input line 902.
\MT@outer@space=\skip51
)
Package microtype Info: Loading configuration file microtype.cfg.
(c:/texlive/2024/texmf-dist/tex/latex/microtype/microtype.cfg
File: microtype.cfg 2024/03/29 v3.1b microtype main configuration file
(RS)
)) (c:/texlive/2024/texmf-dist/tex/latex/euler/euler.sty
Package: euler 1995/03/05 v2.5
Package: `euler' v2.5 <1995/03/05> (FJ and FMi)
LaTeX Font Info:    Redeclaring symbol font `letters' on input line 35.
LaTeX Font Info:    Encoding `OML' has changed to `U' for symbol font
(Font)              `letters' in the math version `normal' on input line
35.
LaTeX Font Info:    Overwriting symbol font `letters' in version `normal'
(Font)                  OML/cmm/m/it --> U/eur/m/n on input line 35.
LaTeX Font Info:    Encoding `OML' has changed to `U' for symbol font
(Font)              `letters' in the math version `bold' on input line
35.
LaTeX Font Info:    Overwriting symbol font `letters' in version `bold'
(Font)                  OML/cmm/b/it --> U/eur/m/n on input line 35.
LaTeX Font Info:    Overwriting symbol font `letters' in version `bold'
(Font)                  U/eur/m/n --> U/eur/b/n on input line 36.
LaTeX Font Info:    Redeclaring math symbol \Gamma on input line 47.
LaTeX Font Info:    Redeclaring math symbol \Delta on input line 48.
LaTeX Font Info:    Redeclaring math symbol \Theta on input line 49.
LaTeX Font Info:    Redeclaring math symbol \Lambda on input line 50.
LaTeX Font Info:    Redeclaring math symbol \Xi on input line 51.
LaTeX Font Info:    Redeclaring math symbol \Pi on input line 52.
LaTeX Font Info:    Redeclaring math symbol \Sigma on input line 53.
LaTeX Font Info:    Redeclaring math symbol \Upsilon on input line 54.
LaTeX Font Info:    Redeclaring math symbol \Phi on input line 55.
LaTeX Font Info:    Redeclaring math symbol \Psi on input line 56.
LaTeX Font Info:    Redeclaring math symbol \Omega on input line 57.
\symEulerFraktur=\mathgroup4
LaTeX Font Info:    Overwriting symbol font `EulerFraktur' in version
`bold'
(Font)                  U/euf/m/n --> U/euf/b/n on input line 63.
LaTeX Info: Redefining \oldstylenums on input line 85.
\symEulerScript=\mathgroup5
LaTeX Font Info:    Overwriting symbol font `EulerScript' in version
`bold'
(Font)                  U/eus/m/n --> U/eus/b/n on input line 93.
LaTeX Font Info:    Redeclaring math symbol \aleph on input line 97.
LaTeX Font Info:    Redeclaring math symbol \Re on input line 98.
LaTeX Font Info:    Redeclaring math symbol \Im on input line 99.
```

```
LaTeX Font Info:    Redeclaring math delimiter \vert on input line 101.
LaTeX Font Info:    Redeclaring math delimiter \backslash on input line
103.
LaTeX Font Info:    Redeclaring math symbol \neg on input line 106.
LaTeX Font Info:    Redeclaring math symbol \wedge on input line 108.
LaTeX Font Info:    Redeclaring math symbol \vee on input line 110.
LaTeX Font Info:    Redeclaring math symbol \setminus on input line 112.
LaTeX Font Info:    Redeclaring math symbol \sim on input line 113.
LaTeX Font Info:    Redeclaring math symbol \mid on input line 114.
LaTeX Font Info:    Redeclaring math delimiter \arrowvert on input line
116.
LaTeX Font Info:    Redeclaring math symbol \mathsection on input line
117.
\symEulerExtension=\mathgroup6
LaTeX Font Info:    Redeclaring math symbol \coprod on input line 125.
LaTeX Font Info:    Redeclaring math symbol \prod on input line 125.
LaTeX Font Info:    Redeclaring math symbol \sum on input line 125.
LaTeX Font Info:    Redeclaring math symbol \intop on input line 130.
LaTeX Font Info:    Redeclaring math symbol \ointop on input line 131.
LaTeX Font Info:    Redeclaring math symbol \braceld on input line 132.
LaTeX Font Info:    Redeclaring math symbol \bracerd on input line 133.
LaTeX Font Info:    Redeclaring math symbol \bracelu on input line 134.
LaTeX Font Info:    Redeclaring math symbol \braceru on input line 135.
LaTeX Font Info:    Redeclaring math symbol \infty on input line 136.
LaTeX Font Info:    Redeclaring math symbol \nearrow on input line 153.
LaTeX Font Info:    Redeclaring math symbol \searrow on input line 154.
LaTeX Font Info:    Redeclaring math symbol \nwarrow on input line 155.
LaTeX Font Info:    Redeclaring math symbol \swarrow on input line 156.
LaTeX Font Info:    Redeclaring math symbol \Leftrightarrow on input line
157.
LaTeX Font Info:    Redeclaring math symbol \Leftarrow on input line 158.
LaTeX Font Info:    Redeclaring math symbol \Rightarrow on input line
159.
LaTeX Font Info:    Redeclaring math symbol \leftrightarrow on input line
160.
LaTeX Font Info:    Redeclaring math symbol \leftarrow on input line 161.
LaTeX Font Info:    Redeclaring math symbol \rightarrow on input line
163.
LaTeX Font Info:    Redeclaring math delimiter \uparrow on input line
166.
LaTeX Font Info:    Redeclaring math delimiter \downarrow on input line
168.
LaTeX Font Info:    Redeclaring math delimiter \updownarrow on input line
170.
LaTeX Font Info:    Redeclaring math delimiter \Uparrow on input line
172.
LaTeX Font Info:    Redeclaring math delimiter \Downarrow on input line
174.
LaTeX Font Info:    Redeclaring math delimiter \Updownarrow on input line
176.
LaTeX Font Info:    Redeclaring math symbol \leftharpoonup on input line
177.
LaTeX Font Info:    Redeclaring math symbol \leftharpoondown on input
line 178.
```

```
LaTeX Font Info:    Redeclaring math symbol \rightharpoonup on input line
179.
LaTeX Font Info:    Redeclaring math symbol \rightharpoondown on input
line 180
.
LaTeX Font Info:    Redeclaring math delimiter \lbrace on input line 182.
LaTeX Font Info:    Redeclaring math delimiter \rbrace on input line 184.
\symcmmigroup=\mathgroup7
LaTeX Font Info:    Overwriting symbol font `cmmigroup' in version `bold'
(Font)                  OML/cmm/m/it --> OML/cmm/b/it on input line 200.
LaTeX Font Info:    Redeclaring math accent \vec on input line 201.
LaTeX Font Info:    Redeclaring math symbol \triangleleft on input line
202.
LaTeX Font Info:    Redeclaring math symbol \triangleright on input line
203.
LaTeX Font Info:    Redeclaring math symbol \star on input line 204.
LaTeX Font Info:    Redeclaring math symbol \lhook on input line 205.
LaTeX Font Info:    Redeclaring math symbol \rhook on input line 206.
LaTeX Font Info:    Redeclaring math symbol \flat on input line 207.
LaTeX Font Info:    Redeclaring math symbol \natural on input line 208.
LaTeX Font Info:    Redeclaring math symbol \sharp on input line 209.
LaTeX Font Info:    Redeclaring math symbol \smile on input line 210.
LaTeX Font Info:    Redeclaring math symbol \frown on input line 211.
LaTeX Font Info:    Redeclaring math accent \grave on input line 245.
LaTeX Font Info:    Redeclaring math accent \acute on input line 246.
LaTeX Font Info:    Redeclaring math accent \tilde on input line 247.
LaTeX Font Info:    Redeclaring math accent \ddot on input line 248.
LaTeX Font Info:    Redeclaring math accent \check on input line 249.
LaTeX Font Info:    Redeclaring math accent \breve on input line 250.
LaTeX Font Info:    Redeclaring math accent \bar on input line 251.
LaTeX Font Info:    Redeclaring math accent \dot on input line 252.
LaTeX Font Info:    Redeclaring math accent \hat on input line 254.
) (c:/texlive/2024/texmf-dist/tex/latex/merriweather/merriweather.sty
Package: merriweather 2022/09/20 (Bob Tennent) Supports
Merriweather(Sans) font
s for all LaTeX engines.
(c:/texlive/2024/texmf-dist/tex/generic/iftex/ifxetex.sty
Package: ifxetex 2019/10/25 v0.7 ifxetex legacy package. Use iftex
instead.
) (c:/texlive/2024/texmf-dist/tex/generic/iftex/ifluatex.sty
Package: ifluatex 2019/10/25 v1.5 ifluatex legacy package. Use iftex
instead.
) (c:/texlive/2024/texmf-dist/tex/latex/base/textcomp.sty
Package: textcomp 2024/04/24 v2.1b Standard LaTeX package
) (c:/texlive/2024/texmf-dist/tex/latex/xkeyval/xkeyval.sty
Package: xkeyval 2022/06/16 v2.9 package option processing (HA)
(c:/texlive/2024/texmf-dist/tex/generic/xkeyval/xkeyval.tex
(c:/texlive/2024/te
xmf-dist/tex/generic/xkeyval/xkvutils.tex
\XKV@toks=\toks22
\XKV@tempa@toks=\toks23
)
\XKV@depth=\count271
```

```
File: xkeyval.tex 2014/12/03 v2.7a key=value parser (HA)
)) (c:/texlive/2024/texmf-dist/tex/latex/base/fontenc.sty
Package: fontenc 2021/04/29 v2.0v Standard LaTeX package
) (c:/texlive/2024/texmf-dist/tex/latex/fontaxes/fontaxes.sty
Package: fontaxes 2020/07/21 v1.0e Font selection axes
LaTeX Info: Redefining \upshape on input line 29.
LaTeX Info: Redefining \itshape on input line 31.
LaTeX Info: Redefining \slshape on input line 33.
LaTeX Info: Redefining \swshape on input line 35.
LaTeX Info: Redefining \scshape on input line 37.
LaTeX Info: Redefining \sscshape on input line 39.
LaTeX Info: Redefining \ulcshape on input line 41.
LaTeX Info: Redefining \textsw on input line 47.
LaTeX Info: Redefining \textssc on input line 48.
LaTeX Info: Redefining \textulc on input line 49.
)) (c:/texlive/2024/texmf-dist/tex/latex/mathastext/mathastext.sty
Package: mathastext 2024/07/27 v1.4b Use the text font in math mode (JFB)

Package mathastext Info: Starting the math mode configuration.
\mst@exists@muskip=\muskip17
\mst@forall@muskip=\muskip18
\mst@prime@muskip=\muskip19
\mst@do@nonletters=\toks24
\mst@undo@nonletters=\toks25
\mst@do@easynonletters=\toks26
\mst@undo@easynonletters=\toks27
\symmtoperatorfont=\mathgroup8
\symmtletterfont=\mathgroup9
(   mathastext:   ) ! and ?
(   mathastext:   ) punctuation: , . : ; and \colon
LaTeX Info: Redefining \relbar on input line 1201.
LaTeX Info: Redefining \rightarrowfill on input line 1202.
LaTeX Info: Redefining \leftarrowfill on input line 1205.
(   mathastext:   ) + and =
LaTeX Info: Redefining \Relbar on input line 1298.
(   mathastext:   ) adding = ; and + to \nfss@catcodes
(   mathastext:   ) parentheses ( ) [ ] and slash /
(   mathastext:   ) alldelims: < > \backslash \setminus | \vert \mid \{
\}
LaTeX Font Info:    Redeclaring math symbol \setminus on input line 1364.
LaTeX Info: Redefining \models on input line 1383.
(   mathastext:   ) \# \mathdollar \% \&
(   mathastext:   ) \imath and \jmath
LaTeX Font Info:    Overwriting math alphabet `\Mathnormalbold' in
version `nor
mal'
(Font)                  T1/Merriwthr-OsF/b/it --> T1/Merriwthr-OsF/b/it
on inpu
t line 2863.
LaTeX Font Info:    Overwriting math alphabet `\Mathnormalbold' in
version `bol
d'
(Font)                  T1/Merriwthr-OsF/b/it --> T1/Merriwthr-OsF/b/it
on inpu
```

```
t line 2863.
LaTeX Font Info:    Overwriting symbol font `mtletterfont' in version
`normal'
(Font)                  T1/Merriwthr-OsF/m/it --> T1/Merriwthr-OsF/m/it
on inpu
t line 2863.
LaTeX Font Info:    Overwriting symbol font `mtletterfont' in version
`bold'
(Font)                  T1/Merriwthr-OsF/m/it --> T1/Merriwthr-OsF/b/it
on inpu
t line 2863.
LaTeX Font Info:    Overwriting symbol font `mtoperatorfont' in version
`normal
'
(Font)                  T1/Merriwthr-OsF/m/n --> T1/Merriwthr-OsF/m/n on
input
line 2863.
LaTeX Font Info:    Overwriting symbol font `mtoperatorfont' in version
`bold'
(Font)                  T1/Merriwthr-OsF/m/n --> T1/Merriwthr-OsF/b/n on
input
line 2863.
LaTeX Font Info:    Overwriting math alphabet `\Mathbf' in version
`normal'
(Font)                  T1/Merriwthr-OsF/b/n --> T1/Merriwthr-OsF/b/n on
input
line 2863.
LaTeX Font Info:    Overwriting math alphabet `\Mathbf' in version `bold'
(Font)                  T1/Merriwthr-OsF/b/n --> T1/Merriwthr-OsF/b/n on
input
line 2863.
LaTeX Font Info:    Overwriting math alphabet `\Mathit' in version
`normal'
(Font)                  T1/Merriwthr-OsF/m/it --> T1/Merriwthr-OsF/m/it
on inpu
t line 2863.
LaTeX Font Info:    Overwriting math alphabet `\Mathit' in version `bold'
(Font)                  T1/Merriwthr-OsF/m/it --> T1/Merriwthr-OsF/b/it
on inpu
t line 2863.
LaTeX Font Info:    Overwriting math alphabet `\Mathsf' in version
`normal'
(Font)                  T1/MerriwthrSans-OsF/m/n --> T1/MerriwthrSans-
OsF/m/n o
n input line 2863.
LaTeX Font Info:    Overwriting math alphabet `\Mathsf' in version `bold'
(Font)                  T1/MerriwthrSans-OsF/m/n --> T1/MerriwthrSans-
OsF/b/n o
n input line 2863.
LaTeX Font Info:    Overwriting math alphabet `\Mathtt' in version
`normal'
(Font)                  T1/lmtt/m/n --> T1/lmtt/m/n on input line 2863.
LaTeX Font Info:    Overwriting math alphabet `\Mathtt' in version `bold'
(Font)                  T1/lmtt/m/n --> T1/lmtt/b/n on input line 2863.
```

```
(   mathastext:   ) Latin letters in the `normal', resp. `bold',
(   mathastext:   ) math versions are now set up to use the fonts
(   mathastext:   ) T1/Merriwthr-OsF/m/it, resp. T1/Merriwthr-OsF/b/it.
(   mathastext:   ) Other characters (digits, ...) and \log-like names
will be
(   mathastext:   ) typeset with the n shape.
(   mathastext:   ) \hbar
(   mathastext:   ) minus as endash
(   mathastext:   ) The italic option is in effect.
(   mathastext:   ) \HUGE has been (re)-defined.
(   mathastext:   ) mathastext has declared larger sizes for subscripts.
(   mathastext:   ) To keep LaTeX defaults, use option
`defaultmathsizes'.

Package mathastext Info: Loading is complete.  You can now use
\Mathastext to
(mathastext)             modify the normal and bold math versions.  Use
it
(mathastext)             with optional argument or use \MTDeclareVersion
to
(mathastext)             declare additional math versions.
) (c:/texlive/2024/texmf-dist/tex/latex/relsize/relsize.sty
Package: relsize 2013/03/29 ver 4.1
) (c:/texlive/2024/texmf-dist/tex/latex/ragged2e/ragged2e.sty
Package: ragged2e 2023/06/22 v3.6 ragged2e Package
\CenteringLeftskip=\skip52
\RaggedLeftLeftskip=\skip53
\RaggedRightLeftskip=\skip54
\CenteringRightskip=\skip55
\RaggedLeftRightskip=\skip56
\RaggedRightRightskip=\skip57
\CenteringParfillskip=\skip58
\RaggedLeftParfillskip=\skip59
\RaggedRightParfillskip=\skip60
\JustifyingParfillskip=\skip61
\CenteringParindent=\skip62
\RaggedLeftParindent=\skip63
\RaggedRightParindent=\skip64
\JustifyingParindent=\skip65
) (c:/texlive/2024/texmf-dist/tex/latex/xcolor/xcolor.sty
Package: xcolor 2023/11/15 v3.01 LaTeX color extensions (UK)
(c:/texlive/2024/texmf-dist/tex/latex/graphics-cfg/color.cfg
File: color.cfg 2016/01/02 v1.6 sample color configuration
)
Package xcolor Info: Driver file: pdftex.def on input line 274.
(c:/texlive/2024/texmf-dist/tex/latex/graphics-def/pdftex.def
File: pdftex.def 2024/04/13 v1.2c Graphics/color driver for pdftex
) (c:/texlive/2024/texmf-dist/tex/latex/graphics/mathcolor.ltx)
Package xcolor Info: Model `cmy' substituted by `cmy0' on input line
1350.
Package xcolor Info: Model `hsb' substituted by `rgb' on input line 1354.
Package xcolor Info: Model `RGB' extended on input line 1366.
Package xcolor Info: Model `HTML' substituted by `rgb' on input line
1368.
```

```
Package xcolor Info: Model `Hsb' substituted by `hsb' on input line 1369.
Package xcolor Info: Model `tHsb' substituted by `hsb' on input line
1370.
Package xcolor Info: Model `HSB' substituted by `hsb' on input line 1371.
Package xcolor Info: Model `Gray' substituted by `gray' on input line
1372.
Package xcolor Info: Model `wave' substituted by `hsb' on input line
1373.
) (c:/texlive/2024/texmf-dist/tex/latex/colortbl/colortbl.sty
Package: colortbl 2024/07/06 v1.0i Color table columns (DPC)
(c:/texlive/2024/texmf-dist/tex/latex/tools/array.sty
Package: array 2024/06/14 v2.6d Tabular extension package (FMi)
\col@sep=\dimen143
\ar@mcellbox=\box53
\extrarowheight=\dimen144
\NC@list=\toks28
\extratabsurround=\skip66
\backup@length=\skip67
\ar@cellbox=\box54
)
\everycr=\toks29
\minrowclearance=\skip68
\rownum=\count272
) (c:/texlive/2024/texmf-dist/tex/latex/graphics/graphicx.sty
Package: graphicx 2021/09/16 v1.2d Enhanced LaTeX Graphics (DPC,SPQR)
(c:/texlive/2024/texmf-dist/tex/latex/graphics/graphics.sty
Package: graphics 2024/05/23 v1.4g Standard LaTeX Graphics (DPC,SPQR)
(c:/texlive/2024/texmf-dist/tex/latex/graphics/trig.sty
Package: trig 2023/12/02 v1.11 sin cos tan (DPC)
) (c:/texlive/2024/texmf-dist/tex/latex/graphics-cfg/graphics.cfg
File: graphics.cfg 2016/06/04 v1.11 sample graphics configuration
)
Package graphics Info: Driver file: pdftex.def on input line 106.
)
\Gin@req@height=\dimen145
\Gin@req@width=\dimen146
) (c:/texlive/2024/texmf-dist/tex/latex/xpatch/xpatch.sty
(c:/texlive/2024/texm
f-dist/tex/latex/l3kernel/expl3.sty
Package: expl3 2024-05-27 L3 programming layer (loader)
(c:/texlive/2024/texmf-dist/tex/latex/l3backend/l3backend-pdftex.def
File: l3backend-pdftex.def 2024-05-08 L3 backend support: PDF output
(pdfTeX)
\l__color_backend_stack_int=\count273
\l__pdf_internal_box=\box55
))
Package: xpatch 2020/03/25 v0.3a Extending etoolbox patching commands
(c:/texlive/2024/texmf-dist/tex/latex/l3packages/xparse/xparse.sty
Package: xparse 2024-05-08 L3 Experimental document command parser
)) (c:/texlive/2024/texmf-dist/tex/latex/environ/environ.sty
Package: environ 2014/05/04 v0.3 A new way to define environments
(c:/texlive/2024/texmf-dist/tex/latex/trimspaces/trimspaces.sty
Package: trimspaces 2009/09/17 v1.1 Trim spaces around a token list
)
```

```
\@envbody=\toks30
) (c:/texlive/2024/texmf-dist/tex/latex/lastpage/lastpage.sty
Package: lastpage 2024/07/07 v2.1c lastpage: 2.09 or 2e? (HMM)
(c:/texlive/2024/texmf-dist/tex/latex/lastpage/lastpage2e.sty
Package: lastpage2e 2024/07/07 v2.1c Decide which 2e lastpage version to
use (H
MM)
(c:/texlive/2024/texmf-dist/tex/latex/lastpage/lastpagemodern.sty
Package: lastpagemodern 2024-07-07 v2.1c Refers to last page's name (HMM;
JPG)
\c@lastpagecount=\count274
)
)) (c:/texlive/2024/texmf-dist/tex/latex/graphics/rotating.sty
Package: rotating 2016/08/11 v2.16d rotated objects in LaTeX
(c:/texlive/2024/texmf-dist/tex/latex/base/ifthen.sty
Package: ifthen 2024/03/16 v1.1e Standard LaTeX ifthen package (DPC)
)
\c@r@tfl@t=\count275
\rotFPtop=\skip69
\rotFPbot=\skip70
\rot@float@box=\box56
\rot@mess@toks=\toks31
) (c:/texlive/2024/texmf-dist/tex/latex/graphics/lscape.sty
Package: lscape 2020/05/28 v3.02 Landscape Pages (DPC)
) (c:/texlive/2024/texmf-dist/tex/latex/tools/afterpage.sty
Package: afterpage 2023/07/04 v1.08 After-Page Package (DPC)
\AP@output=\toks32
\AP@partial=\box57
\AP@footins=\box58
) (c:/texlive/2024/texmf-dist/tex/latex/textpos/textpos.sty
Package: textpos 2022/07/23 v1.10.1
Package textpos Info: choosing support for LaTeX3 on input line 60.
\TP@textbox=\box59
\TP@holdbox=\box60
\TPHorizModule=\dimen147
\TPVertModule=\dimen148
\TP@margin=\dimen149
\TP@absmargin=\dimen150
Grid set 16 x 16 = 37.34424pt x 52.81541pt
\TPboxrulesize=\dimen151
\TP@ox=\dimen152
\TP@oy=\dimen153
\TP@tbargs=\toks33
TextBlockOrigin set to 0pt x 0pt
) (c:/texlive/2024/texmf-dist/tex/latex/url/url.sty
\Urlmuskip=\muskip20
Package: url 2013/09/16  ver 3.4  Verb mode for urls, etc.
) (c:/texlive/2024/texmf-dist/tex/latex/newfloat/newfloat.sty
Package: newfloat 2023/10/01 v1.2 Defining new floating environments (AR)
Package newfloat Info: `rotating' package detected.
) (c:/texlive/2024/texmf-dist/tex/latex/mdframed/mdframed.sty
Package: mdframed 2013/07/01 1.9b: mdframed
(c:/texlive/2024/texmf-dist/tex/latex/kvoptions/kvoptions.sty
```

```
Package: kvoptions 2022-06-15 v3.15 Key value format for package options
(HO)
(c:/texlive/2024/texmf-dist/tex/generic/ltxcmds/ltxcmds.sty
Package: ltxcmds 2023-12-04 v1.26 LaTeX kernel commands for general use
(HO)
) (c:/texlive/2024/texmf-dist/tex/latex/kvsetkeys/kvsetkeys.sty
Package: kvsetkeys 2022-10-05 v1.19 Key value parser (HO)
)) (c:/texlive/2024/texmf-dist/tex/latex/zref/zref-abspage.sty
Package: zref-abspage 2023-09-14 v2.35 Module abspage for zref (HO)
(c:/texlive/2024/texmf-dist/tex/latex/zref/zref-base.sty
Package: zref-base 2023-09-14 v2.35 Module base for zref (HO)
(c:/texlive/2024/texmf-dist/tex/generic/infwarerr/infwarerr.sty
Package: infwarerr 2019/12/03 v1.5 Providing info/warning/error messages
(HO)
) (c:/texlive/2024/texmf-dist/tex/generic/kvdefinekeys/kvdefinekeys.sty
Package: kvdefinekeys 2019-12-19 v1.6 Define keys (HO)
) (c:/texlive/2024/texmf-dist/tex/generic/pdftexcmds/pdftexcmds.sty
Package: pdftexcmds 2020-06-27 v0.33 Utility functions of pdfTeX for
LuaTeX (HO
)
Package pdftexcmds Info: \pdf@primitive is available.
Package pdftexcmds Info: \pdf@ifprimitive is available.
Package pdftexcmds Info: \pdfdraftmode found.
) (c:/texlive/2024/texmf-dist/tex/generic/etexcmds/etexcmds.sty
Package: etexcmds 2019/12/15 v1.7 Avoid name clashes with e-TeX commands
(HO)
) (c:/texlive/2024/texmf-dist/tex/latex/auxhook/auxhook.sty
Package: auxhook 2019-12-17 v1.6 Hooks for auxiliary files (HO)
)
Package zref Info: New property list: main on input line 767.
Package zref Info: New property: default on input line 768.
Package zref Info: New property: page on input line 769.
)
\c@abspage=\count276
Package zref Info: New property: abspage on input line 67.
) (c:/texlive/2024/texmf-dist/tex/latex/needspace/needspace.sty
Package: needspace 2010/09/12 v1.3d reserve vertical space
)
\mdf@templength=\skip71
\c@mdf@globalstyle@cnt=\count277
\mdf@skipabove@length=\skip72
\mdf@skipbelow@length=\skip73
\mdf@leftmargin@length=\skip74
\mdf@rightmargin@length=\skip75
\mdf@innerleftmargin@length=\skip76
\mdf@innerrightmargin@length=\skip77
\mdf@innertopmargin@length=\skip78
\mdf@innerbottommargin@length=\skip79
\mdf@splittopskip@length=\skip80
\mdf@splitbottomskip@length=\skip81
\mdf@outermargin@length=\skip82
\mdf@innermargin@length=\skip83
\mdf@linewidth@length=\skip84
\mdf@innerlinewidth@length=\skip85
```

```
\mdf@middlelinewidth@length=\skip86
\mdf@outerlinewidth@length=\skip87
\mdf@roundcorner@length=\skip88
\mdf@footenotedistance@length=\skip89
\mdf@userdefinedwidth@length=\skip90
\mdf@needspace@length=\skip91
\mdf@frametitleaboveskip@length=\skip92
\mdf@frametitlebelowskip@length=\skip93
\mdf@frametitlerulewidth@length=\skip94
\mdf@frametitleleftmargin@length=\skip95
\mdf@frametitlerightmargin@length=\skip96
\mdf@shadowsize@length=\skip97
\mdf@extratopheight@length=\skip98
\mdf@subtitleabovelinewidth@length=\skip99
\mdf@subtitlebelowlinewidth@length=\skip100
\mdf@subtitleaboveskip@length=\skip101
\mdf@subtitlebelowskip@length=\skip102
\mdf@subtitleinneraboveskip@length=\skip103
\mdf@subtitleinnerbelowskip@length=\skip104
\mdf@subsubtitleabovelinewidth@length=\skip105
\mdf@subsubtitlebelowlinewidth@length=\skip106
\mdf@subsubtitleaboveskip@length=\skip107
\mdf@subsubtitlebelowskip@length=\skip108
\mdf@subsubtitleinneraboveskip@length=\skip109
\mdf@subsubtitleinnerbelowskip@length=\skip110
(c:/texlive/2024/texmf-dist/tex/latex/mdframed/md-frame-0.mdf
File: md-frame-0.mdf 2013/07/01\ 1.9b: md-frame-0
)
\mdf@frametitlebox=\box61
\mdf@footnotebox=\box62
\mdf@splitbox@one=\box63
\mdf@splitbox@two=\box64
\mdf@splitbox@save=\box65
\mdfsplitboxwidth=\skip111
\mdfsplitboxtotalwidth=\skip112
\mdfsplitboxheight=\skip113
\mdfsplitboxdepth=\skip114
\mdfsplitboxtotalheight=\skip115
\mdfframetitleboxwidth=\skip116
\mdfframetitleboxtotalwidth=\skip117
\mdfframetitleboxheight=\skip118
\mdfframetitleboxdepth=\skip119
\mdfframetitleboxtotalheight=\skip120
\mdffootnoteboxwidth=\skip121
\mdffootnoteboxtotalwidth=\skip122
\mdffootnoteboxheight=\skip123
\mdffootnoteboxdepth=\skip124
\mdffootnoteboxtotalheight=\skip125
\mdftotallinewidth=\skip126
\mdfboundingboxwidth=\skip127
\mdfboundingboxtotalwidth=\skip128
\mdfboundingboxheight=\skip129
\mdfboundingboxdepth=\skip130
\mdfboundingboxtotalheight=\skip131
```

```
\mdf@freevspace@length=\skip132
\mdf@horizontalwidthofbox@length=\skip133
\mdf@verticalmarginwhole@length=\skip134
\mdf@horizontalspaceofbox=\skip135
\mdfsubtitleheight=\skip136
\mdfsubsubtitleheight=\skip137
\c@mdfcountframes=\count278

****** mdframed patching \endmdf@trivlist

****** -- success******

\mdf@envdepth=\count279
\c@mdf@env@i=\count280
\c@mdf@env@ii=\count281
\c@mdf@zref@counter=\count282
Package zref Info: New property: mdf@pagevalue on input line 895.
) (c:/texlive/2024/texmf-dist/tex/latex/titlesec/titlesec.sty
Package: titlesec 2023/10/27 v2.16 Sectioning titles
\ttl@box=\box66
\beforetitleunit=\skip138
\aftertitleunit=\skip139
\ttl@plus=\dimen154
\ttl@minus=\dimen155
\ttl@toksa=\toks34
\titlewidth=\dimen156
\titlewidthlast=\dimen157
\titlewidthfirst=\dimen158
) (c:/texlive/2024/texmf-dist/tex/latex/koma-script/scrextend.sty
Package: scrextend 2023/07/07 v3.41 KOMA-Script package (extend other
classes w
ith features of KOMA-Script classes)
(c:/texlive/2024/texmf-dist/tex/latex/koma-script/scrkbase.sty
Package: scrkbase 2023/07/07 v3.41 KOMA-Script package (KOMA-Script-
dependent b
asics and keyval usage)
(c:/texlive/2024/texmf-dist/tex/latex/koma-script/scrbase.sty
Package: scrbase 2023/07/07 v3.41 KOMA-Script package (KOMA-Script-
independent
basics and keyval usage)
(c:/texlive/2024/texmf-dist/tex/latex/koma-script/scrlfile.sty
Package: scrlfile 2023/07/07 v3.41 KOMA-Script package (file load hooks)
(c:/texlive/2024/texmf-dist/tex/latex/koma-script/scrlfile-hook.sty
Package: scrlfile-hook 2023/07/07 v3.41 KOMA-Script package (using LaTeX
hooks)

(c:/texlive/2024/texmf-dist/tex/latex/koma-script/scrlogo.sty
Package: scrlogo 2023/07/07 v3.41 KOMA-Script package (logo)
)))
Applying: [2021/05/01] Usage of raw or classic option list on input line
252.
Already applied: [0000/00/00] Usage of raw or classic option list on
input line
 368.
```

```
))
Package scrextend Info: unexpected definition of `\@makefnmark'.
(scrextend)             Trying to patch it on input line 1762.
Package scrextend Info: patch seems to be successfull on input line 1762.
)

LaTeX Font Warning: Font shape `T1/cmr/m/n' in size <7.5> not available
(Font)              size <7> substituted on input line 69.

(c:/texlive/2024/texmf-dist/tex/latex/tools/calc.sty
Package: calc 2023/07/08 v4.3 Infix arithmetic (KKT,FJ)
\calc@Acount=\count283
\calc@Bcount=\count284
\calc@Adimen=\dimen159
\calc@Bdimen=\dimen160
\calc@Askip=\skip140
\calc@Bskip=\skip141
LaTeX Info: Redefining \setlength on input line 80.
LaTeX Info: Redefining \addtolength on input line 81.
\calc@Ccount=\count285
\calc@Cskip=\skip142
) (c:/texlive/2024/texmf-dist/tex/latex/geometry/geometry.sty
Package: geometry 2020/01/02 v5.9 Page Geometry
(c:/texlive/2024/texmf-dist/tex/generic/iftex/ifvtex.sty
Package: ifvtex 2019/10/25 v1.7 ifvtex legacy package. Use iftex instead.
)
\Gm@cnth=\count286
\Gm@cntv=\count287
\c@Gm@tempcnt=\count288
\Gm@bindingoffset=\dimen161
\Gm@wd@mp=\dimen162
\Gm@odd@mp=\dimen163
\Gm@even@mp=\dimen164
\Gm@layoutwidth=\dimen165
\Gm@layoutheight=\dimen166
\Gm@layouthoffset=\dimen167
\Gm@layoutvoffset=\dimen168
\Gm@dimlist=\toks35
) (c:/texlive/2024/texmf-dist/tex/latex/preprint/authblk.sty
Package: authblk 2001/02/27 1.3 (PWD)
\affilsep=\skip143
\@affilsep=\skip144
\c@Maxaffil=\count289
\c@authors=\count290
\c@affil=\count291
) (c:/texlive/2024/texmf-dist/tex/latex/footmisc/footmisc.sty
Package: footmisc 2023/07/05 v6.0f a miscellany of footnote facilities
\FN@temptoken=\toks36
\footnotemargin=\dimen169
\@outputbox@depth=\dimen170
Package footmisc Info: Declaring symbol style bringhurst on input line
696.
Package footmisc Info: Declaring symbol style chicago on input line 704.
Package footmisc Info: Declaring symbol style wiley on input line 713.
```

```
Package footmisc Info: Declaring symbol style lamport-robust on input
line 724.

Package footmisc Info: Declaring symbol style lamport* on input line 744.
Package footmisc Info: Declaring symbol style lamport*-robust on input
line 765
.
) (c:/texlive/2024/texmf-dist/tex/latex/fancyhdr/fancyhdr.sty
Package: fancyhdr 2024/07/23 v4.3.1 Extensive control of page headers and
foote
rs
\f@nch@headwidth=\skip145
\f@nch@O@elh=\skip146
\f@nch@O@erh=\skip147
\f@nch@O@olh=\skip148
\f@nch@O@orh=\skip149
\f@nch@O@elf=\skip150
\f@nch@O@erf=\skip151
\f@nch@O@olf=\skip152
\f@nch@O@orf=\skip153
) (c:/texlive/2024/texmf-dist/tex/generic/alphalph/alphalph.sty
Package: alphalph 2019/12/09 v2.6 Convert numbers to letters (HO)
(c:/texlive/2024/texmf-dist/tex/generic/intcalc/intcalc.sty
Package: intcalc 2019/12/15 v1.3 Expandable calculations with integers
(HO)
))
\c@authorfn=\count292
(c:/texlive/2024/texmf-dist/tex/latex/abstract/abstract.sty
Package: abstract 2009/06/08 v1.2a configurable abstracts
\abstitleskip=\skip154
\absleftindent=\skip155
\absrightindent=\skip156
\absparindent=\skip157
\absparsep=\skip158
)
Package newfloat Info: New float `keypoints' with options
`placement=t!,name=kp
t' on input line 291.
\c@keypoints=\count293
\newfloat@ftype=\count294
Package newfloat Info: float type `keypoints'=8 on input line 291.
(c:/texlive/2024/texmf-dist/tex/latex/enumitem/enumitem.sty
Package: enumitem 2019/06/20 v3.9 Customized lists
\labelindent=\skip159
\enit@outerparindent=\dimen171
\enit@toks=\toks37
\enit@inbox=\box67
\enit@count@id=\count295
\enitdp@description=\count296
) (c:/texlive/2024/texmf-dist/tex/latex/quoting/quoting.sty
Package: quoting 2014/01/28 v0.1c Consolidated environment for displayed
text
\quo@toppartop=\skip160
) (c:/texlive/2024/texmf-dist/tex/latex/sttools/stfloats.sty
```

```
Package: stfloats 2017/03/27 v3.3 Improve float mechanism and
baselineskip sett
ings
\@dblbotnum=\count297
\c@dblbotnumber=\count298
) (c:/texlive/2024/texmf-dist/tex/latex/booktabs/booktabs.sty
Package: booktabs 2020/01/12 v1.61803398 Publication quality tables
\heavyrulewidth=\dimen172
\lightrulewidth=\dimen173
\cmidrulewidth=\dimen174
\belowrulesep=\dimen175
\belowbottomsep=\dimen176
\aboverulesep=\dimen177
\abovetopsep=\dimen178
\cmidrulesep=\dimen179
\cmidrulekern=\dimen180
\defaultaddspace=\dimen181
\@cmidla=\count299
\@cmidlb=\count300
\@aboverulesep=\dimen182
\@belowrulesep=\dimen183
\@thisruleclass=\count301
\@lastruleclass=\count302
\@thisrulewidth=\dimen184
) (c:/texlive/2024/texmf-dist/tex/latex/tools/tabularx.sty
Package: tabularx 2023/12/11 v2.12a `tabularx' package (DPC)
\TX@col@width=\dimen185
\TX@old@table=\dimen186
\TX@old@col=\dimen187
\TX@target=\dimen188
\TX@delta=\dimen189
\TX@cols=\count303
\TX@ftn=\toks38
)
\enitdp@tablenotes=\count304
(c:/texlive/2024/texmf-dist/tex/latex/caption/caption.sty
Package: caption 2023/08/05 v3.6o Customizing captions (AR)
(c:/texlive/2024/texmf-dist/tex/latex/caption/caption3.sty
Package: caption3 2023/07/31 v2.4d caption3 kernel (AR)
\caption@tempdima=\dimen190
\captionmargin=\dimen191
\caption@leftmargin=\dimen192
\caption@rightmargin=\dimen193
\caption@width=\dimen194
\caption@indent=\dimen195
\caption@parindent=\dimen196
\caption@hangindent=\dimen197
Package caption Info: Standard document class detected.
)
\c@caption@flags=\count305
\c@continuedfloat=\count306
Package caption Info: rotating package is loaded.
Package caption Info: scrextend package is loaded.
\caption@addmargin@hsize=\dimen198
```

```
\caption@addmargin@linewidth=\dimen199
) (c:/texlive/2024/texmf-dist/tex/latex/natbib/natbib.sty
Package: natbib 2010/09/13 8.31b (PWD, AO)
\bibhang=\skip161
\bibsep=\skip162
LaTeX Info: Redefining \cite on input line 694.
\c@NAT@ctr=\count307
)) (c:/texlive/2024/texmf-dist/tex/latex/svg/svg.sty
Package: svg 2020/11/26 v2.02k (include SVG pictures)
(c:/texlive/2024/texmf-dist/tex/latex/tools/shellesc.sty
Package: shellesc 2023/07/08 v1.0d unified shell escape interface for
LaTeX
Package shellesc Info: Restricted shell escape enabled on input line 77.
)
\c@svg@param@lastpage=\count308
\svg@box=\box68
\c@svg@param@currpage=\count309
) (c:/texlive/2024/texmf-dist/tex/latex/transparent/transparent.sty
Package: transparent 2022-10-27 v1.5 Transparency with color stacks
(c:/texlive/2024/texmf-dist/tex/latex/transparent/transparent-
nometadata.sty
Package: transparent-nometadata 2022-10-27 v1.5 Transparency via pdfTeX's
color
 stack (HO)
)) (c:/texlive/2024/texmf-dist/tex/latex/pgf/systemlayer/pgfsys.sty
(c:/texlive
/2024/texmf-dist/tex/latex/pgf/utilities/pgfrcs.sty
(c:/texlive/2024/texmf-dist
/tex/generic/pgf/utilities/pgfutil-common.tex
\pgfutil@everybye=\toks39
\pgfutil@tempdima=\dimen256
\pgfutil@tempdimb=\dimen257
) (c:/texlive/2024/texmf-dist/tex/generic/pgf/utilities/pgfutil-latex.def
\pgfutil@abb=\box69
) (c:/texlive/2024/texmf-dist/tex/generic/pgf/utilities/pgfrcs.code.tex
(c:/tex
live/2024/texmf-dist/tex/generic/pgf/pgf.revision.tex)
Package: pgfrcs 2023-01-15 v3.1.10 (3.1.10)
)) (c:/texlive/2024/texmf-
dist/tex/generic/pgf/systemlayer/pgfsys.code.tex
Package: pgfsys 2023-01-15 v3.1.10 (3.1.10)
(c:/texlive/2024/texmf-dist/tex/generic/pgf/utilities/pgfkeys.code.tex
\pgfkeys@pathtoks=\toks40
\pgfkeys@temptoks=\toks41

(c:/texlive/2024/texmf-
dist/tex/generic/pgf/utilities/pgfkeyslibraryfiltered.co
de.tex
\pgfkeys@tmptoks=\toks42
))
\pgf@x=\dimen258
\pgf@y=\dimen259
\pgf@xa=\dimen260
\pgf@ya=\dimen261
```

```
\pgf@xb=\dimen262
\pgf@yb=\dimen263
\pgf@xc=\dimen264
\pgf@yc=\dimen265
\pgf@xd=\dimen266
\pgf@yd=\dimen267
\w@pgf@writea=\write3
\r@pgf@reada=\read2
\c@pgf@counta=\count310
\c@pgf@countb=\count311
\c@pgf@countc=\count312
\c@pgf@countd=\count313
\t@pgf@toka=\toks43
\t@pgf@tokb=\toks44
\t@pgf@tokc=\toks45
\pgf@sys@id@count=\count314
(c:/texlive/2024/texmf-dist/tex/generic/pgf/systemlayer/pgf.cfg
File: pgf.cfg 2023-01-15 v3.1.10 (3.1.10)
)
Driver file for pgf: pgfsys-pdftex.def
(c:/texlive/2024/texmf-dist/tex/generic/pgf/systemlayer/pgfsys-pdftex.def
File: pgfsys-pdftex.def 2023-01-15 v3.1.10 (3.1.10)
(c:/texlive/2024/texmf-dist/tex/generic/pgf/systemlayer/pgfsys-common-
pdf.def
File: pgfsys-common-pdf.def 2023-01-15 v3.1.10 (3.1.10)
)))
(c:/texlive/2024/texmf-
dist/tex/generic/pgf/systemlayer/pgfsyssoftpath.code.tex
File: pgfsyssoftpath.code.tex 2023-01-15 v3.1.10 (3.1.10)
\pgfsyssoftpath@smallbuffer@items=\count315
\pgfsyssoftpath@bigbuffer@items=\count316
)
(c:/texlive/2024/texmf-
dist/tex/generic/pgf/systemlayer/pgfsysprotocol.code.tex
File: pgfsysprotocol.code.tex 2023-01-15 v3.1.10 (3.1.10)
)) (c:/texlive/2024/texmf-dist/tex/latex/siunitx/siunitx.sty
Package: siunitx 2024-06-24 v3.3.19 A comprehensive (SI) units package
\l__siunitx_number_uncert_offset_int=\count317
\l__siunitx_number_exponent_fixed_int=\count318
\l__siunitx_number_min_decimal_int=\count319
\l__siunitx_number_min_integer_int=\count320
\l__siunitx_number_round_precision_int=\count321
\l__siunitx_number_lower_threshold_int=\count322
\l__siunitx_number_upper_threshold_int=\count323
\l__siunitx_number_group_first_int=\count324
\l__siunitx_number_group_size_int=\count325
\l__siunitx_number_group_minimum_int=\count326
\l__siunitx_angle_tmp_dim=\dimen268
\l__siunitx_angle_marker_box=\box70
\l__siunitx_angle_unit_box=\box71
\l__siunitx_compound_count_int=\count327
(c:/texlive/2024/texmf-dist/tex/latex/translations/translations.sty
Package: translations 2022/02/05 v1.12 internationalization of LaTeX2e
packages
```

```
 (CN)
) (c:/texlive/2024/texmf-dist/tex/latex/amsmath/amstext.sty
Package: amstext 2021/08/26 v2.01 AMS text
(c:/texlive/2024/texmf-dist/tex/latex/amsmath/amsgen.sty
File: amsgen.sty 1999/11/30 v2.0 generic functions
\@emptytoks=\toks46
\ex@=\dimen269
))
\l__siunitx_table_tmp_box=\box72
\l__siunitx_table_tmp_dim=\dimen270
\l__siunitx_table_column_width_dim=\dimen271
\l__siunitx_table_integer_box=\box73
\l__siunitx_table_decimal_box=\box74
\l__siunitx_table_uncert_box=\box75
\l__siunitx_table_before_box=\box76
\l__siunitx_table_after_box=\box77
\l__siunitx_table_before_dim=\dimen272
\l__siunitx_table_carry_dim=\dimen273
\l__siunitx_unit_tmp_int=\count328
\l__siunitx_unit_position_int=\count329
\l__siunitx_unit_total_int=\count330
) (c:/texlive/2024/texmf-dist/tex/latex/framed/framed.sty
Package: framed 2011/10/22 v 0.96: framed or shaded text with page breaks
\OuterFrameSep=\skip163
\fb@frw=\dimen274
\fb@frh=\dimen275
\FrameRule=\dimen276
\FrameSep=\dimen277
) (c:/texlive/2024/texmf-dist/tex/generic/soul/soul.sty
Package: soul 2023-06-14 v3.1 Permit use of UTF-8 characters in soul (HO)
(c:/texlive/2024/texmf-dist/tex/generic/soul/soul-ori.sty
Package: soul-ori 2023-06-14 v3.1 letterspacing/underlining (mf)
\SOUL@word=\toks47
\SOUL@lasttoken=\toks48
\SOUL@syllable=\toks49
\SOUL@cmds=\toks50
\SOUL@buffer=\toks51
\SOUL@token=\toks52
\SOUL@syllgoal=\dimen278
\SOUL@syllwidth=\dimen279
\SOUL@charkern=\dimen280
\SOUL@hyphkern=\dimen281
\SOUL@dimen=\dimen282
\SOUL@dimeni=\dimen283
\SOUL@minus=\count331
\SOUL@comma=\count332
\SOUL@apo=\count333
\SOUL@grave=\count334
\SOUL@spaceskip=\skip164
\SOUL@ttwidth=\dimen284
\SOUL@uldp=\dimen285
\SOUL@ulht=\dimen286
)) (c:/texlive/2024/texmf-dist/tex/latex/hyperref/hyperref.sty
Package: hyperref 2024-07-10 v7.01j Hypertext links for LaTeX
```

```
(c:/texlive/2024/texmf-dist/tex/generic/pdfescape/pdfescape.sty
Package: pdfescape 2019/12/09 v1.15 Implements pdfTeX's escape features
(HO)
) (c:/texlive/2024/texmf-dist/tex/latex/hycolor/hycolor.sty
Package: hycolor 2020-01-27 v1.10 Color options for hyperref/bookmark
(HO)
) (c:/texlive/2024/texmf-dist/tex/latex/hyperref/nameref.sty
Package: nameref 2023-11-26 v2.56 Cross-referencing by name of section
(c:/texlive/2024/texmf-dist/tex/latex/refcount/refcount.sty
Package: refcount 2019/12/15 v3.6 Data extraction from label references
(HO)
) (c:/texlive/2024/texmf-
dist/tex/generic/gettitlestring/gettitlestring.sty
Package: gettitlestring 2019/12/15 v1.6 Cleanup title references (HO)
)
\c@section@level=\count335
) (c:/texlive/2024/texmf-dist/tex/generic/stringenc/stringenc.sty
Package: stringenc 2019/11/29 v1.12 Convert strings between diff.
encodings (HO
)
)
\@linkdim=\dimen287
\Hy@linkcounter=\count336
\Hy@pagecounter=\count337
(c:/texlive/2024/texmf-dist/tex/latex/hyperref/pd1enc.def
File: pd1enc.def 2024-07-10 v7.01j Hyperref: PDFDocEncoding definition
(HO)
Now handling font encoding PD1 ...
... no UTF-8 mapping file for font encoding PD1
)
\Hy@SavedSpaceFactor=\count338
(c:/texlive/2024/texmf-dist/tex/latex/hyperref/puenc.def
File: puenc.def 2024-07-10 v7.01j Hyperref: PDF Unicode definition (HO)
Now handling font encoding PU ...
... no UTF-8 mapping file for font encoding PU
)
Package hyperref Info: Option `colorlinks' set `true' on input line 4040.
Package hyperref Info: Hyper figures OFF on input line 4157.
Package hyperref Info: Link nesting OFF on input line 4162.
Package hyperref Info: Hyper index ON on input line 4165.
Package hyperref Info: Plain pages OFF on input line 4172.
Package hyperref Info: Backreferencing OFF on input line 4177.
Package hyperref Info: Implicit mode ON; LaTeX internals redefined.
Package hyperref Info: Bookmarks ON on input line 4424.
\c@Hy@tempcnt=\count339
LaTeX Info: Redefining \url on input line 4763.
\XeTeXLinkMargin=\dimen288
(c:/texlive/2024/texmf-dist/tex/generic/bitset/bitset.sty
Package: bitset 2019/12/09 v1.3 Handle bit-vector datatype (HO)
(c:/texlive/2024/texmf-dist/tex/generic/bigintcalc/bigintcalc.sty
Package: bigintcalc 2019/12/15 v1.5 Expandable calculations on big
integers (HO
)
))
```

```
\Fld@menulength=\count340
\Field@Width=\dimen289
\Fld@charsize=\dimen290
Package hyperref Info: Hyper figures OFF on input line 6042.
Package hyperref Info: Link nesting OFF on input line 6047.
Package hyperref Info: Hyper index ON on input line 6050.
Package hyperref Info: backreferencing OFF on input line 6057.
Package hyperref Info: Link coloring ON on input line 6060.
Package hyperref Info: Link coloring with OCG OFF on input line 6067.
Package hyperref Info: PDF/A mode OFF on input line 6072.
(c:/texlive/2024/texmf-dist/tex/latex/base/atbegshi-ltx.sty
Package: atbegshi-ltx 2021/01/10 v1.0c Emulation of the original atbegshi
package with kernel methods
)
\Hy@abspage=\count341
\c@Item=\count342
\c@Hfootnote=\count343
)
Package hyperref Info: Driver (autodetected): hpdftex.
(c:/texlive/2024/texmf-dist/tex/latex/hyperref/hpdftex.def
File: hpdftex.def 2024-07-10 v7.01j Hyperref driver for pdfTeX
(c:/texlive/2024/texmf-dist/tex/latex/base/atveryend-ltx.sty
Package: atveryend-ltx 2020/08/19 v1.0a Emulation of the original
atveryend pac
kage
with kernel methods
)
\HyAnn@Count=\count344
\Fld@listcount=\count345
\c@bookmark@seq@number=\count346
(c:/texlive/2024/texmf-dist/tex/latex/rerunfilecheck/rerunfilecheck.sty
Package: rerunfilecheck 2022-07-10 v1.10 Rerun checks for auxiliary files
(HO)
(c:/texlive/2024/texmf-dist/tex/generic/uniquecounter/uniquecounter.sty
Package: uniquecounter 2019/12/15 v1.4 Provide unlimited unique counter
(HO)
)
Package uniquecounter Info: New unique counter `rerunfilecheck' on input
line 2
85.
)
\Hy@SectionHShift=\skip165
)
Package translations Info: No language package found. I am going to use
`englis
h' as default language. on input line 66.
LaTeX Font Info:    Trying to load font information for T1+Merriwthr-OsF
on inp
ut line 66.
(c:/texlive/2024/texmf-dist/tex/latex/merriweather/T1Merriwthr-OsF.fd
File: T1Merriwthr-OsF.fd 2020/08/30 (autoinst) Font definitions for
T1/Merriwth
r-OsF.
)
```

```
LaTeX Font Info:    Font shape `T1/Merriwthr-OsF/m/n' will be
(Font)              scaled to size 7.5pt on input line 66.
(./HVSeeke-Manuscript.aux)
\openout1 = `HVSeeke-Manuscript.aux'.

LaTeX Font Info:    Checking defaults for OML/cmm/m/it on input line 66.
LaTeX Font Info:    ... okay on input line 66.
LaTeX Font Info:    Checking defaults for OMS/cmsy/m/n on input line 66.
LaTeX Font Info:    ... okay on input line 66.
LaTeX Font Info:    Checking defaults for OT1/cmr/m/n on input line 66.
LaTeX Font Info:    ... okay on input line 66.
LaTeX Font Info:    Checking defaults for T1/cmr/m/n on input line 66.
LaTeX Font Info:    ... okay on input line 66.
LaTeX Font Info:    Checking defaults for TS1/cmr/m/n on input line 66.
LaTeX Font Info:    ... okay on input line 66.
LaTeX Font Info:    Checking defaults for OMX/cmex/m/n on input line 66.
LaTeX Font Info:    ... okay on input line 66.
LaTeX Font Info:    Checking defaults for U/cmr/m/n on input line 66.
LaTeX Font Info:    ... okay on input line 66.
LaTeX Font Info:    Checking defaults for PD1/pdf/m/n on input line 66.
LaTeX Font Info:    ... okay on input line 66.
LaTeX Font Info:    Checking defaults for PU/pdf/m/n on input line 66.
LaTeX Font Info:    ... okay on input line 66.
LaTeX Info: Redefining \microtypecontext on input line 66.
Package microtype Info: Applying patch `item' on input line 66.
Package microtype Info: Applying patch `toc' on input line 66.
Package microtype Info: Applying patch `eqnum' on input line 66.
Package microtype Info: Applying patch `footnote' on input line 66.
Package microtype Info: Applying patch `verbatim' on input line 66.
Package microtype Info: Generating PDF output.
Package microtype Info: Character protrusion enabled (level 2).
Package microtype Info: Using default protrusion set `alltext'.
Package microtype Info: Automatic font expansion enabled (level 2),
(microtype)             stretch: 20, shrink: 20, step: 1, non-selected.
Package microtype Info: Using default expansion set `alltext-nott'.
LaTeX Info: Redefining \showhyphens on input line 66.
Package microtype Info: No adjustment of tracking.
Package microtype Info: No adjustment of interword spacing.
Package microtype Info: No adjustment of character kerning.
Package microtype Info: Loading generic protrusion settings for font
family
(microtype)             `Merriwthr-OsF' (encoding: T1).
(microtype)             For optimal results, create family-specific
settings.
(microtype)             See the microtype manual for details.
LaTeX Font Info:    Redeclaring symbol font `operators' on input line 66.
LaTeX Font Info:    Encoding `OT1' has changed to `T1' for symbol font
(Font)              `operators' in the math version `normal' on input
line 66.
LaTeX Font Info:    Overwriting symbol font `operators' in version
`normal'
(Font)                  OT1/cmr/m/n --> T1/Merriwthr-OsF/m/up on input
line 66.
```

```
LaTeX Font Info:    Encoding `OT1' has changed to `T1' for symbol font
(Font)              `operators' in the math version `bold' on input line
66.
LaTeX Font Info:    Overwriting symbol font `operators' in version `bold'
(Font)                  OT1/cmr/bx/n --> T1/Merriwthr-OsF/m/up on input
line 66
.
LaTeX Font Info:    Overwriting symbol font `operators' in version `bold'
(Font)                  T1/Merriwthr-OsF/m/up --> T1/Merriwthr-OsF/b/up
on inpu
t line 66.
LaTeX Font Info:    Redeclaring math alphabet \mathbf on input line 66.
LaTeX Font Info:    Overwriting math alphabet `\mathbf' in version
`normal'
(Font)                  OT1/cmr/bx/n --> T1/Merriwthr-OsF/b/up on input
line 66
.
LaTeX Font Info:    Overwriting math alphabet `\mathbf' in version `bold'
(Font)                  OT1/cmr/bx/n --> T1/Merriwthr-OsF/b/up on input
line 66
.
LaTeX Font Info:    Redeclaring math alphabet \mathsf on input line 66.
LaTeX Font Info:    Overwriting math alphabet `\mathsf' in version
`normal'
(Font)                  OT1/cmss/m/n --> T1/MerriwthrSans-OsF/m/up on
input lin
e 66.
LaTeX Font Info:    Overwriting math alphabet `\mathsf' in version `bold'
(Font)                  OT1/cmss/bx/n --> T1/MerriwthrSans-OsF/m/up on
input li
ne 66.
LaTeX Font Info:    Redeclaring math alphabet \mathit on input line 66.
LaTeX Font Info:    Overwriting math alphabet `\mathit' in version
`normal'
(Font)                  OT1/cmr/m/it --> T1/Merriwthr-OsF/m/it on input
line 66
.
LaTeX Font Info:    Overwriting math alphabet `\mathit' in version `bold'
(Font)                  OT1/cmr/bx/it --> T1/Merriwthr-OsF/m/it on input
line 6
6.
LaTeX Font Info:    Redeclaring math alphabet \mathtt on input line 66.
LaTeX Font Info:    Overwriting math alphabet `\mathtt' in version
`normal'
(Font)                  OT1/cmtt/m/n --> T1/lmtt/m/up on input line 66.
LaTeX Font Info:    Overwriting math alphabet `\mathtt' in version `bold'
(Font)                  OT1/cmtt/m/n --> T1/lmtt/m/up on input line 66.
LaTeX Font Info:    Overwriting math alphabet `\mathsf' in version `bold'
(Font)                  T1/MerriwthrSans-OsF/m/up --> T1/MerriwthrSans-
OsF/b/up
 on input line 66.
LaTeX Font Info:    Overwriting math alphabet `\mathit' in version `bold'
(Font)                  T1/Merriwthr-OsF/m/it --> T1/Merriwthr-OsF/b/it
on inpu
```

```
t line 66.
\c@mv@tabular=\count347
\c@mv@boldtabular=\count348
(c:/texlive/2024/texmf-dist/tex/context/base/mkii/supp-pdf.mkii
[Loading MPS to PDF converter (version 2006.09.02).]
\scratchcounter=\count349
\scratchdimen=\dimen291
\scratchbox=\box78
\nofMPsegments=\count350
\nofMParguments=\count351
\everyMPshowfont=\toks53
\MPscratchCnt=\count352
\MPscratchDim=\dimen292
\MPnumerator=\count353
\makeMPintoPDFobject=\count354
\everyMPtoPDFconversion=\toks54
) (c:/texlive/2024/texmf-dist/tex/latex/epstopdf-pkg/epstopdf-base.sty
Package: epstopdf-base 2020-01-24 v2.11 Base part for package epstopdf
Package epstopdf-base Info: Redefining graphics rule for `.eps' on input
line 4
85.
(c:/texlive/2024/texmf-dist/tex/latex/latexconfig/epstopdf-sys.cfg
File: epstopdf-sys.cfg 2010/07/13 v1.3 Configuration of (r)epstopdf for
TeX Liv
e
))
*geometry* driver: auto-detecting
*geometry* detected driver: pdftex
*geometry* verbose mode - [ preamble ] result:
* driver: pdftex
* paper: a4paper
* layout: <same size as paper>
* layoutoffset:(h,v)=(0.0pt,0.0pt)
* modes: includefoot twoside
* h-part:(L,W,R)=(54.64pt, 488.22787pt, 54.64pt)
* v-part:(T,H,B)=(66.0pt, 745.04684pt, 34.0pt)
* \paperwidth=597.50787pt
* \paperheight=845.04684pt
* \textwidth=488.22787pt
* \textheight=715.04684pt
* \oddsidemargin=-17.62999pt
* \evensidemargin=-17.62999pt
* \topmargin=-47.76999pt
* \headheight=17.5pt
* \headsep=24.0pt
* \topskip=10.0pt
* \footskip=30.0pt
* \marginparwidth=48.0pt
* \marginparsep=10.0pt
* \columnsep=18.0pt
* \skip\footins=22.0pt plus 2.0pt
* \hoffset=0.0pt
* \voffset=0.0pt
* \mag=1000
```

```
* \@twocolumntrue
* \@twosidetrue
* \@mparswitchtrue
* \@reversemarginfalse
* (1in=72.27pt=25.4mm, 1cm=28.453pt)

Package caption Info: Begin \AtBeginDocument code.
Package caption Info: hyperref package is loaded.
Package caption Info: End \AtBeginDocument code.

(c:/texlive/2024/texmf-dist/tex/latex/translations/translations-basic-
dictionar
y-english.trsl
File: translations-basic-dictionary-english.trsl (english translation
file `tra
nslations-basic-dictionary')
)
Package translations Info: loading dictionary `translations-basic-
dictionary' f
or `english'. on input line 66.
Package hyperref Info: Link coloring ON on input line 66.
(./HVSeeke-Manuscript.out) (./HVSeeke-Manuscript.out)
\@outlinefile=\write4
\openout4 = `HVSeeke-Manuscript.out'.

\@gscitedetails=\box79
\@gscitedetailsheight=\skip166
\@gsheadbox=\box80
\@gsheadboxheight=\skip167
LaTeX Font Info:    Font shape `T1/Merriwthr-OsF/b/n' will be
(Font)              scaled to size 6.5pt on input line 66.
LaTeX Font Info:    Calculating math sizes for size <7.5> on input line
66.

LaTeX Font Warning: Font shape `T1/Merriwthr-OsF/m/up' undefined
(Font)              using `T1/Merriwthr-OsF/m/n' instead on input line
66.

LaTeX Font Info:    Font shape `T1/Merriwthr-OsF/m/up' will be
(Font)              scaled to size 6.24973pt on input line 66.
LaTeX Font Info:    Font shape `T1/Merriwthr-OsF/m/up' will be
(Font)              scaled to size 5.24997pt on input line 66.
LaTeX Font Info:    Trying to load font information for U+eur on input
line 66.

(c:/texlive/2024/texmf-dist/tex/latex/amsfonts/ueur.fd
File: ueur.fd 2013/01/14 v3.01 Euler Roman
) (c:/texlive/2024/texmf-dist/tex/latex/microtype/mt-eur.cfg
File: mt-eur.cfg 2006/07/31 v1.1 microtype config. file: AMS Euler Roman
(RS)
)

LaTeX Font Warning: Font shape `OMS/cmsy/m/n' in size <7.5> not available
(Font)              size <7> substituted on input line 66.
```

```
LaTeX Font Info:    External font `cmex10' loaded for size
(Font)              <7.5> on input line 66.
LaTeX Font Info:    External font `cmex10' loaded for size
(Font)              <6.24973> on input line 66.
LaTeX Font Info:    External font `cmex10' loaded for size
(Font)              <5.24997> on input line 66.
LaTeX Font Info:    Trying to load font information for U+euf on input
line 66.

(c:/texlive/2024/texmf-dist/tex/latex/amsfonts/ueuf.fd
File: ueuf.fd 2013/01/14 v3.01 Euler Fraktur
) (c:/texlive/2024/texmf-dist/tex/latex/microtype/mt-euf.cfg
File: mt-euf.cfg 2006/07/03 v1.1 microtype config. file: AMS Euler
Fraktur (RS)

)
LaTeX Font Info:    Trying to load font information for U+eus on input
line 66.

(c:/texlive/2024/texmf-dist/tex/latex/amsfonts/ueus.fd
File: ueus.fd 2013/01/14 v3.01 Euler Script
) (c:/texlive/2024/texmf-dist/tex/latex/microtype/mt-eus.cfg
File: mt-eus.cfg 2006/07/28 v1.2 microtype config. file: AMS Euler Script
(RS)
)
LaTeX Font Info:    Trying to load font information for U+euex on input
line 66
.
(c:/texlive/2024/texmf-dist/tex/latex/amsfonts/ueuex.fd
File: ueuex.fd 2013/01/14 v3.01 Euler extra symbols
)

LaTeX Font Warning: Font shape `OML/cmm/m/it' in size <7.5> not available
(Font)              size <7> substituted on input line 66.

LaTeX Font Info:    Font shape `T1/Merriwthr-OsF/m/n' will be
(Font)              scaled to size 6.24973pt on input line 66.
LaTeX Font Info:    Font shape `T1/Merriwthr-OsF/m/n' will be
(Font)              scaled to size 5.24997pt on input line 66.
LaTeX Font Info:    Font shape `T1/Merriwthr-OsF/m/it' will be
(Font)              scaled to size 7.5pt on input line 66.
LaTeX Font Info:    Font shape `T1/Merriwthr-OsF/m/it' will be
(Font)              scaled to size 6.24973pt on input line 66.
LaTeX Font Info:    Font shape `T1/Merriwthr-OsF/m/it' will be
(Font)              scaled to size 5.24997pt on input line 66.
LaTeX Font Info:    Font shape `T1/Merriwthr-OsF/m/n' will be
(Font)              scaled to size 8.0pt on input line 66.
LaTeX Font Info:    Font shape `T1/Merriwthr-OsF/m/it' will be
(Font)              scaled to size 8.0pt on input line 66.
LaTeX Font Info:    Font shape `T1/Merriwthr-OsF/b/it' will be
(Font)              scaled to size 8.0pt on input line 66.
TextBlockOrigin set to 4pc+6.64pt x 4pc+6pt
<oup.pdf, id=108, 597.50829pt x 845.0471pt>
```

```
File: oup.pdf Graphic file (type pdf)
<use oup.pdf>
Package pdftex.def Info: oup.pdf  used on input line 96.
(pdftex.def)             Requested size: 41.03665pt x 58.038pt.
<gigascience-logo.pdf, id=109, 99.37125pt x 33.12375pt>
File: gigascience-logo.pdf Graphic file (type pdf)
<use gigascience-logo.pdf>
Package pdftex.def Info: gigascience-logo.pdf  used on input line 96.
(pdftex.def)             Requested size: 126.00902pt x 42.0pt.

Overfull \hbox (54.64pt too wide) in paragraph at lines 96--96
[][]
 []

LaTeX Font Info:    Font shape `T1/Merriwthr-OsF/m/n' will be
(Font)              scaled to size 14.0pt on input line 96.
LaTeX Font Info:    Font shape `T1/Merriwthr-OsF/m/n' will be
(Font)              scaled to size 8.99997pt on input line 96.
LaTeX Font Info:    Calculating math sizes for size <14> on input line
96.
LaTeX Font Info:    Font shape `T1/Merriwthr-OsF/m/up' will be
(Font)              scaled to size 14.0pt on input line 96.
LaTeX Font Info:    Font shape `T1/Merriwthr-OsF/m/up' will be
(Font)              scaled to size 11.66617pt on input line 96.
LaTeX Font Info:    Font shape `T1/Merriwthr-OsF/m/up' will be
(Font)              scaled to size 9.79996pt on input line 96.
LaTeX Font Info:    External font `cmex10' loaded for size
(Font)              <14> on input line 96.
LaTeX Font Info:    External font `cmex10' loaded for size
(Font)              <11.66617> on input line 96.
LaTeX Font Info:    External font `cmex10' loaded for size
(Font)              <9.79996> on input line 96.
LaTeX Font Info:    Font shape `T1/Merriwthr-OsF/m/n' will be
(Font)              scaled to size 11.66617pt on input line 96.
LaTeX Font Info:    Font shape `T1/Merriwthr-OsF/m/n' will be
(Font)              scaled to size 9.79996pt on input line 96.
LaTeX Font Info:    Font shape `T1/Merriwthr-OsF/m/it' will be
(Font)              scaled to size 14.0pt on input line 96.
LaTeX Font Info:    Font shape `T1/Merriwthr-OsF/m/it' will be
(Font)              scaled to size 11.66617pt on input line 96.
LaTeX Font Info:    Font shape `T1/Merriwthr-OsF/m/it' will be
(Font)              scaled to size 9.79996pt on input line 96.
LaTeX Font Info:    Font shape `T1/Merriwthr-OsF/b/n' will be
(Font)              scaled to size 18.0pt on input line 96.
LaTeX Font Info:    Font shape `T1/Merriwthr-OsF/m/n' will be
(Font)              scaled to size 13.0pt on input line 96.
LaTeX Font Info:    Calculating math sizes for size <13> on input line
96.
LaTeX Font Info:    Font shape `T1/Merriwthr-OsF/m/up' will be
(Font)              scaled to size 13.0pt on input line 96.
LaTeX Font Info:    Font shape `T1/Merriwthr-OsF/m/up' will be
(Font)              scaled to size 10.83287pt on input line 96.
LaTeX Font Info:    Font shape `T1/Merriwthr-OsF/m/up' will be
(Font)              scaled to size 9.09996pt on input line 96.
```

```
LaTeX Font Warning: Font shape `OMS/cmsy/m/n' in size <13> not available
(Font)              size <12> substituted on input line 96.

LaTeX Font Info:    External font `cmex10' loaded for size
(Font)              <13> on input line 96.
LaTeX Font Info:    External font `cmex10' loaded for size
(Font)              <10.83287> on input line 96.
LaTeX Font Info:    External font `cmex10' loaded for size
(Font)              <9.09996> on input line 96.

LaTeX Font Warning: Font shape `OML/cmm/m/it' in size <13> not available
(Font)              size <12> substituted on input line 96.

LaTeX Font Info:    Font shape `T1/Merriwthr-OsF/m/n' will be
(Font)              scaled to size 10.83287pt on input line 96.
LaTeX Font Info:    Font shape `T1/Merriwthr-OsF/m/n' will be
(Font)              scaled to size 9.09996pt on input line 96.
LaTeX Font Info:    Font shape `T1/Merriwthr-OsF/m/it' will be
(Font)              scaled to size 13.0pt on input line 96.
LaTeX Font Info:    Font shape `T1/Merriwthr-OsF/m/it' will be
(Font)              scaled to size 10.83287pt on input line 96.
LaTeX Font Info:    Font shape `T1/Merriwthr-OsF/m/it' will be
(Font)              scaled to size 9.09996pt on input line 96.
LaTeX Font Info:    Trying to load font information for TS1+Merriwthr-OsF
on in
put line 96.
(c:/texlive/2024/texmf-dist/tex/latex/merriweather/TS1Merriwthr-OsF.fd
File: TS1Merriwthr-OsF.fd 2020/08/30 (autoinst) Font definitions for
TS1/Merriw
thr-OsF.
)
LaTeX Font Info:    Font shape `TS1/Merriwthr-OsF/m/n' will be
(Font)              scaled to size 10.83287pt on input line 96.
Package microtype Info: Loading generic protrusion settings for font
family
(microtype)             `Merriwthr-OsF' (encoding: TS1).
(microtype)             For optimal results, create family-specific
settings.
(microtype)             See the microtype manual for details.
LaTeX Font Info:    Font shape `T1/Merriwthr-OsF/m/n' will be
(Font)              scaled to size 9.0pt on input line 96.
LaTeX Font Info:    Font shape `T1/Merriwthr-OsF/m/up' will be
(Font)              scaled to size 9.0pt on input line 96.
LaTeX Font Info:    Font shape `T1/Merriwthr-OsF/m/up' will be
(Font)              scaled to size 7.0pt on input line 96.
LaTeX Font Info:    Font shape `T1/Merriwthr-OsF/m/up' will be
(Font)              scaled to size 5.0pt on input line 96.
LaTeX Font Info:    External font `cmex10' loaded for size
(Font)              <9> on input line 96.
LaTeX Font Info:    External font `cmex10' loaded for size
(Font)              <7> on input line 96.
LaTeX Font Info:    External font `cmex10' loaded for size
(Font)              <5> on input line 96.
```

```
LaTeX Font Info:    Font shape `T1/Merriwthr-OsF/m/n' will be
(Font)              scaled to size 7.0pt on input line 96.
LaTeX Font Info:    Font shape `T1/Merriwthr-OsF/m/n' will be
(Font)              scaled to size 5.0pt on input line 96.
LaTeX Font Info:    Font shape `T1/Merriwthr-OsF/m/it' will be
(Font)              scaled to size 9.0pt on input line 96.
LaTeX Font Info:    Font shape `T1/Merriwthr-OsF/m/it' will be
(Font)              scaled to size 7.0pt on input line 96.
LaTeX Font Info:    Font shape `T1/Merriwthr-OsF/m/it' will be
(Font)              scaled to size 5.0pt on input line 96.
LaTeX Font Info:    Font shape `T1/Merriwthr-OsF/m/n' will be
(Font)              scaled to size 6.5pt on input line 96.
LaTeX Font Info:    Calculating math sizes for size <6.5> on input line
96.
LaTeX Font Info:    Font shape `T1/Merriwthr-OsF/m/up' will be
(Font)              scaled to size 6.5pt on input line 96.
LaTeX Font Info:    Font shape `T1/Merriwthr-OsF/m/up' will be
(Font)              scaled to size 5.41643pt on input line 96.
LaTeX Font Info:    Font shape `T1/Merriwthr-OsF/m/up' will be
(Font)              scaled to size 4.54997pt on input line 96.

LaTeX Font Warning: Font shape `OMS/cmsy/m/n' in size <6.5> not available
(Font)              size <6> substituted on input line 96.


LaTeX Font Warning: Font shape `OMS/cmsy/m/n' in size <5.41643> not
available
(Font)              size <5> substituted on input line 96.


LaTeX Font Warning: Font shape `OMS/cmsy/m/n' in size <4.54997> not
available
(Font)              size <5> substituted on input line 96.

LaTeX Font Info:    External font `cmex10' loaded for size
(Font)              <6.5> on input line 96.
LaTeX Font Info:    External font `cmex10' loaded for size
(Font)              <5.41643> on input line 96.
LaTeX Font Info:    External font `cmex10' loaded for size
(Font)              <4.54997> on input line 96.

LaTeX Font Warning: Font shape `OML/cmm/m/it' in size <6.5> not available
(Font)              size <6> substituted on input line 96.


LaTeX Font Warning: Font shape `OML/cmm/m/it' in size <5.41643> not
available
(Font)              size <5> substituted on input line 96.


LaTeX Font Warning: Font shape `OML/cmm/m/it' in size <4.54997> not
available
(Font)              size <5> substituted on input line 96.
```

```
LaTeX Font Info:    Font shape `T1/Merriwthr-OsF/m/n' will be
(Font)              scaled to size 5.41643pt on input line 96.
LaTeX Font Info:    Font shape `T1/Merriwthr-OsF/m/n' will be
(Font)              scaled to size 4.54997pt on input line 96.
LaTeX Font Info:    Font shape `T1/Merriwthr-OsF/m/it' will be
(Font)              scaled to size 6.5pt on input line 96.
LaTeX Font Info:    Font shape `T1/Merriwthr-OsF/m/it' will be
(Font)              scaled to size 5.41643pt on input line 96.
LaTeX Font Info:    Font shape `T1/Merriwthr-OsF/m/it' will be
(Font)              scaled to size 4.54997pt on input line 96.
LaTeX Font Info:    Font shape `TS1/Merriwthr-OsF/m/n' will be
(Font)              scaled to size 5.41643pt on input line 96.

Overfull \hbox (54.64pt too wide) in paragraph at lines 96--96
[][][]
 []

LaTeX Font Info:    Font shape `T1/Merriwthr-OsF/b/n' will be
(Font)              scaled to size 10.0pt on input line 96.
LaTeX Font Info:    Font shape `T1/Merriwthr-OsF/b/n' will be
(Font)              scaled to size 8.0pt on input line 96.
\myboxwidth=\skip168

Overfull \hbox (54.64pt too wide) in paragraph at lines 96--96
[][][]
 []

Package mdframed Info: mdframed works in twoside mode on input line 100.
LaTeX Font Info:    Font shape `T1/Merriwthr-OsF/b/n' will be
(Font)              scaled to size 8.2pt on input line 100.
LaTeX Font Info:    Font shape `TS1/Merriwthr-OsF/m/n' will be
(Font)              scaled to size 7.5pt on input line 103.
Package mdframed Info: mdframed inside float
 mdframed uses option nobreak mdframed on input line 110.
Package mdframed Info: mdframed inside a box
 mdframed uses option nobreak mdframed on input line 110.
LaTeX Font Info:    Font shape `T1/Merriwthr-OsF/b/n' will be
(Font)              scaled to size 7.5pt on input line 116.

Package natbib Warning: Citation `Paez2016virome' on page 1 undefined on
input
line 116.


Package natbib Warning: Citation `woolhouse2007pathogenes' on page 1
undefined
on input line 116.


Package natbib Warning: Citation `zhang_cai_tan_lu_jiang_zhang_peng_2019'
on pa
ge 1 undefined on input line 116.
```

```
Package natbib Warning: Citation `vander_elst_meyer_2018' on page 1
undefined o
n input line 116.


Package natbib Warning: Citation `vander_elst_meyer_2018' on page 1
undefined o
n input line 117.


Underfull \vbox (badness 10000) has occurred while \output is active []



Overfull \vbox (22.43274pt too high) has occurred while \output is active
[]



LaTeX Font Info:    Font shape `T1/Merriwthr-OsF/m/n' will be
(Font)              scaled to size 7.8pt on input line 118.
LaTeX Font Info:    Font shape `T1/Merriwthr-OsF/b/n' will be
(Font)              scaled to size 7.8pt on input line 118.
[1{c:/texlive/2024/texmf-
var/fonts/map/pdftex/updmap/pdftex.map}{c:/texlive/202
4/texmf-
dist/fonts/enc/dvips/merriweather/merriwthr_posqbl.enc}{c:/texlive/2024
/texmf-dist/fonts/enc/dvips/merriweather/merriwthr_owzwzj.enc}


 <./oup.pdf> <./gigascience-logo.pdf>]

Package natbib Warning: Citation `stone_campbell_grant_mcauliffe_2019' on
page
2 undefined on input line 120.


Package natbib Warning: Citation `weber' on page 2 undefined on input
line 121.



Package natbib Warning: Citation `weber' on page 2 undefined on input
line 121.



Package natbib Warning: Citation `brown_2002' on page 2 undefined on
input line
 121.
```

Package natbib Warning: Citation
`tonkovic_kalajdziski_zdravevski_lameski_coriz
zo_pires_garcia_loncar-turukalo_trajkovik_2020' on page 2 undefined on
input li
ne 121.

Package natbib Warning: Citation
`tonkovic_kalajdziski_zdravevski_lameski_coriz
zo_pires_garcia_loncar-turukalo_trajkovik_2020' on page 2 undefined on
input li
ne 121.

Package natbib Warning: Citation
`alves_westmann_lovate_de_siqueira_borelli_gua
zzaroni_2018' on page 2 undefined on input line 121.

Package natbib Warning: Citation `Wood2019' on page 2 undefined on input
line 1
28.

Package natbib Warning: Citation `Kim2016' on page 2 undefined on input
line 12
8.

Package natbib Warning: Citation `Pratas2018' on page 2 undefined on
input line
 128.

Package natbib Warning: Citation
`pappas_roux_hölzer_lamkiewicz_mock_marz_duti
lh_2021' on page 2 undefined on input line 129.

Package natbib Warning: Citation `strous_kraft_bisdorf_tegetmeyer_2012'
on page
 2 undefined on input line 132.

Package natbib Warning: Citation
`alneberg_bjarnason_de_bruijn_schirmer_quick_i
jaz_lahti_loman_andersson_quince_et_al._2014' on page 2 undefined on
input line
 132.

Package natbib Warning: Citation
`tonkovic_kalajdziski_zdravevski_lameski_coriz

```
zo_pires_garcia_loncar-turukalo_trajkovik_2020' on page 2 undefined on
input li
ne 133.


Package natbib Warning: Citation
`khandelwal_sharma_agrawal_shrivastava_2017' o
n page 2 undefined on input line 135.


Package natbib Warning: Citation `raza-k' on page 2 undefined on input
line 135
.


Package natbib Warning: Citation
`tonkovic_kalajdziski_zdravevski_lameski_coriz
zo_pires_garcia_loncar-turukalo_trajkovik_2020' on page 2 undefined on
input li
ne 136.


Package natbib Warning: Citation `ren_ahlgren_lu_fuhrman_sun_2017' on
page 2 un
defined on input line 139.


Package natbib Warning: Citation
`ren_song_deng_ahlgren_fuhrman_li_xie_poplin_s
un_2020' on page 2 undefined on input line 139.



Package natbib Warning: Citation
`auslander_gussow_benler_wolf_koonin_2020' on
page 2 undefined on input line 142.


Package natbib Warning: Citation `liu_miao_liu_hou_2020' on page 2
undefined on
 input line 142.


Package natbib Warning: Citation `PPR_META' on page 2 undefined on input
line 1
42.


Package natbib Warning: Citation `mock_viehweger_barth_marz_2020' on page
2 und
efined on input line 143.
```

```
Underfull \hbox (badness 1502) in paragraph at lines 142--144
\T1/Merriwthr-OsF/m/n/7.5 (+20) also uti-liz-ing LSTMs and out-per-forms
other
state-of-the-art
 []


Underfull \hbox (badness 1077) in paragraph at lines 142--144
\T1/Merriwthr-OsF/m/n/7.5 (+20) that clas-si-fies con-tigs into phage,
plas-mid
, and chro-mo-some
 []


Underfull \hbox (badness 10000) in paragraph at lines 142--144

 []

LaTeX Font Info:    Font shape `T1/Merriwthr-OsF/b/n' will be
(Font)              scaled to size 8.5pt on input line 158.

Underfull \vbox (badness 10000) has occurred while \output is active []



LaTeX Font Info:    Font shape `T1/Merriwthr-OsF/m/n' will be
(Font)              scaled to size 8.5pt on input line 158.
LaTeX Font Info:    Font shape `T1/Merriwthr-OsF/m/it' will be
(Font)              scaled to size 7.8pt on input line 158.
[2]

Package natbib Warning: Citation `NCBI' on page 3 undefined on input line
159.


Package natbib Warning: Citation `grigoriev2012genome' on page 3
undefined on i
nput line 159.


Package natbib Warning: Citation `nordberg2014genome' on page 3 undefined
on in
put line 159.


LaTeX Warning: Reference `fig:padding' on page 3 undefined on input line
174.


LaTeX Warning: Reference `fig:contigs_assembly' on page 3 undefined on
input li
ne 174.
```

```
LaTeX Warning: Reference `fig:sliding_window' on page 3 undefined on
input line
 174.


Underfull \hbox (badness 1383) in paragraph at lines 176--177
[]\T1/Merriwthr-OsF/m/n/7.5 (+20) Our model for clas-si-fy-ing pro-teins,
based
 on their amino
 []


Underfull \hbox (badness 10000) in paragraph at lines 176--177

 []




LaTeX Warning: File `images/windows_combined.pdf' not found on input line
183.


! Package pdftex.def Error: File `images/windows_combined.pdf' not found:
using
 draft setting.

See the pdftex.def package documentation for explanation.
Type  H <return>  for immediate help.
 ...

l.183 ...s[scale=0.5]{images/windows_combined.pdf}

Try typing  <return>  to proceed.
If that doesn't work, type  X <return>  to quit.

LaTeX Font Info:    Trying to load font information for T1+lmtt on input
line 1
83.
(c:/texlive/2024/texmf-dist/tex/latex/lm/t1lmtt.fd
File: t1lmtt.fd 2015/05/01 v1.6.1 Font defs for Latin Modern
)
Package microtype Info: Loading generic protrusion settings for font
family
(microtype)             `lmtt' (encoding: T1).
(microtype)             For optimal results, create family-specific
settings.
(microtype)             See the microtype manual for details.
LaTeX Font Info:    Font shape `T1/Merriwthr-OsF/m/n' will be
(Font)              scaled to size 6.0pt on input line 184.
LaTeX Font Info:    Font shape `T1/Merriwthr-OsF/b/n' will be
(Font)              scaled to size 6.0pt on input line 184.
```

```
Package natbib Warning: Citation `brandes2022proteinbert' on page 3
undefined o
n input line 200.

LaTeX Font Info:    Font shape `T1/Merriwthr-OsF/b/sl' in size <7.5> not
availa
ble
(Font)              Font shape `T1/Merriwthr-OsF/b/it' tried instead on
input l
ine 202.
LaTeX Font Info:    Font shape `T1/Merriwthr-OsF/b/it' will be
(Font)              scaled to size 7.5pt on input line 202.
LaTeX Font Info:    Font shape `T1/Merriwthr-OsF/m/up' will be
(Font)              scaled to size 7.5pt on input line 203.

! Package svg Error: File `dna_model2.svg' is missing.

See the svg package documentation for explanation.
Type  H <return>  for immediate help.
 ...

l.208 ...udesvg[scale=0.25]{images/dna_model2.svg}

There's no file `dna_model2.svg'
in folder `images/'.


Package natbib Warning: Citation `brandes2022proteinbert' on page 3
undefined o
n input line 215.


Underfull \vbox (badness 10000) has occurred while \output is active []



LaTeX Font Info:    Calculating math sizes for size <7.8> on input line
216.
LaTeX Font Info:    Font shape `T1/Merriwthr-OsF/m/up' will be
(Font)              scaled to size 7.8pt on input line 216.
LaTeX Font Info:    Font shape `T1/Merriwthr-OsF/m/up' will be
(Font)              scaled to size 6.49971pt on input line 216.
LaTeX Font Info:    Font shape `T1/Merriwthr-OsF/m/up' will be
(Font)              scaled to size 5.45998pt on input line 216.

LaTeX Font Warning: Font shape `OMS/cmsy/m/n' in size <6.49971> not
available
(Font)              size <6> substituted on input line 216.


LaTeX Font Warning: Font shape `OMS/cmsy/m/n' in size <5.45998> not
available
(Font)              size <5> substituted on input line 216.
```

```
LaTeX Font Info:    External font `cmex10' loaded for size
(Font)              <7.8> on input line 216.
LaTeX Font Info:    External font `cmex10' loaded for size
(Font)              <6.49971> on input line 216.
LaTeX Font Info:    External font `cmex10' loaded for size
(Font)              <5.45998> on input line 216.

LaTeX Font Warning: Font shape `OML/cmm/m/it' in size <6.49971> not
available
(Font)              size <6> substituted on input line 216.


LaTeX Font Warning: Font shape `OML/cmm/m/it' in size <5.45998> not
available
(Font)              size <5> substituted on input line 216.

LaTeX Font Info:    Font shape `T1/Merriwthr-OsF/m/n' will be
(Font)              scaled to size 6.49971pt on input line 216.
LaTeX Font Info:    Font shape `T1/Merriwthr-OsF/m/n' will be
(Font)              scaled to size 5.45998pt on input line 216.
LaTeX Font Info:    Font shape `T1/Merriwthr-OsF/m/it' will be
(Font)              scaled to size 6.49971pt on input line 216.
LaTeX Font Info:    Font shape `T1/Merriwthr-OsF/m/it' will be
(Font)              scaled to size 5.45998pt on input line 216.
[3{c:/texlive/2024/texmf-dist/fonts/enc/dvips/lm/lm-ec.enc}]


LaTeX Warning: File
`images/DNA_Sequence_Prediction_Experiments_Comparison_Metr
ics-rolf.pdf' not found on input line 268.


! Package pdftex.def Error: File
`images/DNA_Sequence_Prediction_Experiments_Co
mparison_Metrics-rolf.pdf' not found: using draft setting.

See the pdftex.def package documentation for explanation.
Type  H <return>  for immediate help.
 ...

l.268 ..._Experiments_Comparison_Metrics-rolf.pdf}

Try typing  <return>  to proceed.
If that doesn't work, type  X <return>  to quit.


Package hyperref Warning: Suppressing empty link on input line 275.



[4]

LaTeX Warning: File `images/train_acc_size_20.png' not found on input
line 280.
```

```
! Package pdftex.def Error: File `images/train_acc_size_20.png' not
found: usin
g draft setting.

See the pdftex.def package documentation for explanation.
Type  H <return>  for immediate help.
 ...

l.280 ...scale=0.30]{images/train_acc_size_20.png}

Try typing  <return>  to proceed.
If that doesn't work, type  X <return>  to quit.


LaTeX Warning: File `images/val_acc_size_20.png' not found on input line
287.


! Package pdftex.def Error: File `images/val_acc_size_20.png' not found:
using
draft setting.

See the pdftex.def package documentation for explanation.
Type  H <return>  for immediate help.
 ...

l.287 ...s[scale=0.35]{images/val_acc_size_20.png}

Try typing  <return>  to proceed.
If that doesn't work, type  X <return>  to quit.


Package natbib Warning: Citation
`auslander_gussow_benler_wolf_koonin_2020' on
page 5 undefined on input line 296.


Package natbib Warning: Citation `liu_miao_liu_hou_2020' on page 5
undefined on
 input line 296.


Package natbib Warning: Citation
`ren_song_deng_ahlgren_fuhrman_li_xie_poplin_s
un_2020' on page 5 undefined on input line 296.


Package natbib Warning: Citation `PPR_META' on page 5 undefined on input
line 2
96.
```

```
Underfull \vbox (badness 10000) has occurred while \output is active []



LaTeX Font Info:    Font shape `T1/Merriwthr-OsF/b/n' will be
(Font)              scaled to size 7.0pt on input line 309.

Overfull \hbox (4.0541pt too wide) in paragraph at lines 306--317
 [][]
 []


Package natbib Warning: Citation `Shah2023ExpandingViralDiversity' on
page 5 un
defined on input line 346.


Underfull \vbox (badness 4181) has occurred while \output is active []



[5]

Package natbib Warning: Citation `mafft' on page 6 undefined on input
line 368.



Package natbib Warning: Citation `HMM' on page 6 undefined on input line
368.


Underfull \vbox (badness 10000) has occurred while \output is active []




Package natbib Warning: Citation `HVSeeker-github' on page 6 undefined on
input
 line 385.


Underfull \hbox (badness 10000) in paragraph at lines 386--387
[]\T1/Merriwthr-OsF/m/up/7.5 (+20) Project home page:
[][]$\T1/lmtt/m/n/7.5 ht
tps : / / github . com / BackofenLab /
 []


Package natbib Warning: Citation `HVSeeker-software' on page 6 undefined
on inp
ut line 392.
```

```
Package natbib Warning: Citation `HVSeeker-DomeML' on page 6 undefined on
input
 line 393.


Package natbib Warning: Citation `HVSeeker-github' on page 6 undefined on
input
 line 400.


Package natbib Warning: Citation `HVSeeker-DomeML' on page 6 undefined on
input
 line 400.


Underfull \hbox (badness 10000) in paragraph at lines 400--402
\T1/Merriwthr-OsF/m/up/7.5 (+20) The used model is avail-able un-der
 []


Underfull \hbox (badness 10000) in paragraph at lines 400--402
\T1/Merriwthr-OsF/m/up/7.5 (+20) https://github.com/BackofenLab/HVSeeker
[\T1/M
erriwthr-OsF/b/n/7.5 (+20) ? \T1/Merriwthr-OsF/m/up/7.5 (+20) ].DOME-ML
 []


Underfull \vbox (badness 2903) has occurred while \output is active []



[6]
No file HVSeeke-Manuscript.bbl.

Package natbib Warning: There were undefined citations.





[7

]
enddocument/afterlastpage: lastpage setting LastPage.
(./HVSeeke-Manuscript.aux)
 ***********
LaTeX2e <2024-06-01> patch level 2
L3 programming layer <2020/03/25>
 ***********

LaTeX Font Warning: Size substitutions with differences
(Font)              up to 1.0pt have occurred.
```

```
LaTeX Font Warning: Some font shapes were not available, defaults
substituted.


LaTeX Warning: There were undefined references.

Package rerunfilecheck Info: File `HVSeeke-Manuscript.out' has not
changed.
(rerunfilecheck)             Checksum:
3814329A2922A173158AB023B78A12DA;5086.
 )
Here is how much of TeX's memory you used:
 26224 strings out of 473583
 521240 string characters out of 5732343
 1978908 words of memory out of 5000000
 48012 multiletter control sequences out of 15000+600000
 1964280 words of font info for 563 fonts, out of 8000000 for 9000
 1141 hyphenation exceptions out of 8191
 123i,13n,131p,1966b,1042s stack positions out of
10000i,1000n,20000p,200000b,200000s
<c:/texlive/2024/texmf-dist/fonts/type1/sorkin/merriweather/Merriwthr-
Bold.pf
b><c:/texlive/2024/texmf-dist/fonts/type1/sorkin/merriweather/Merriwthr-
BoldIta
lic.pfb><c:/texlive/2024/texmf-
dist/fonts/type1/sorkin/merriweather/Merriwthr-I
talic.pfb><c:/texlive/2024/texmf-
dist/fonts/type1/sorkin/merriweather/Merriwthr
-Regular.pfb><c:/texlive/2024/texmf-
dist/fonts/type1/public/amsfonts/cm/cmsy7.p
fb><c:/texlive/2024/texmf-dist/fonts/type1/public/lm/lmtt8.pfb>
Output written on HVSeeke-Manuscript.pdf (7 pages, 474725 bytes).
PDF statistics:
 261 PDF objects out of 1000 (max. 8388607)
 223 compressed objects within 3 object streams
 47 named destinations out of 1000 (max. 500000)
 211679 words of extra memory for PDF output out of 221844 (max.
10000000)
```

Click here to access/download;LaTeX - Other (clo, cls, sty etc.);example-grid-100x100pt.png

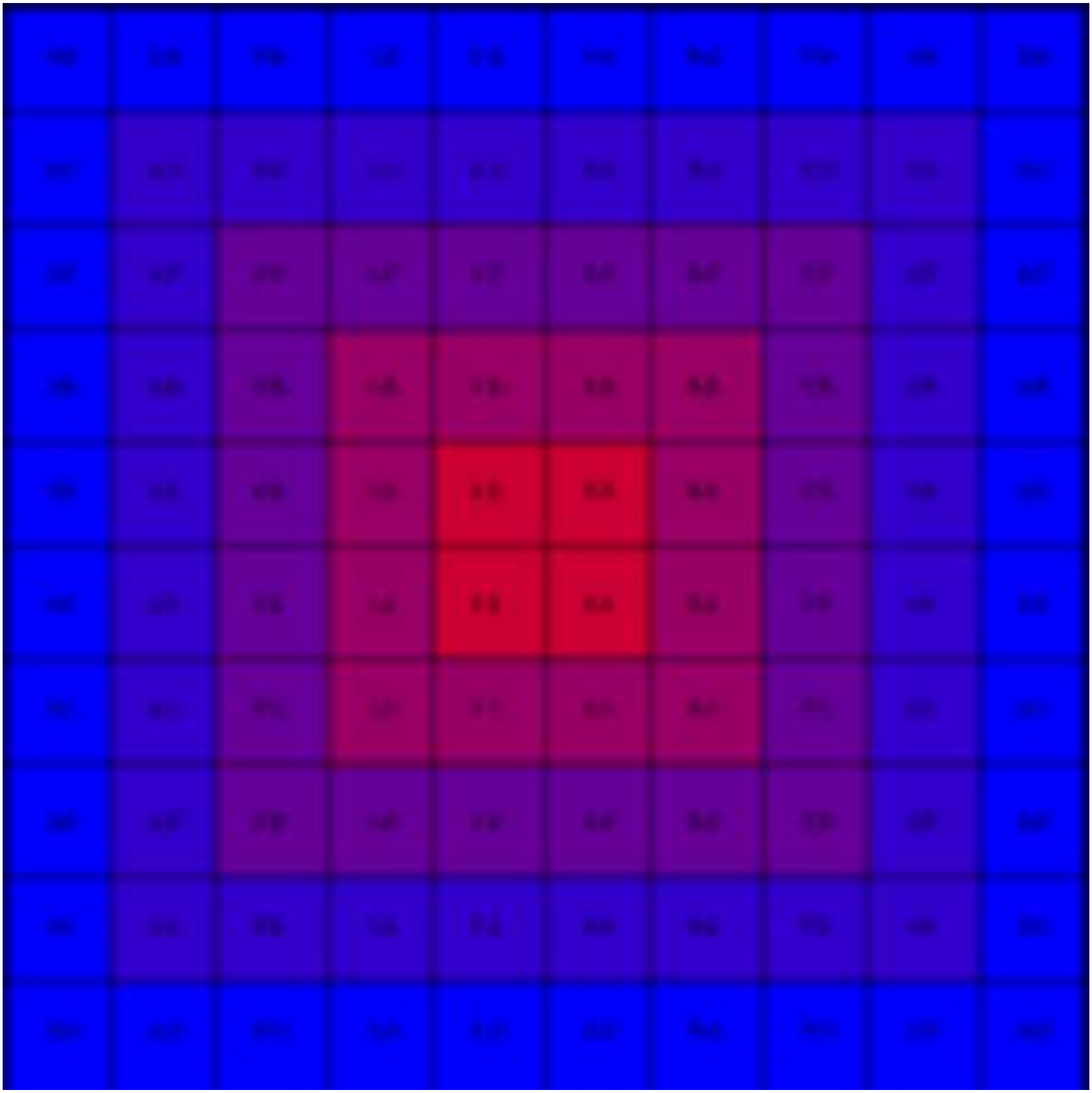

Click here to access/download;LaTeX - Other (clo, cls, sty etc.);example-image.png

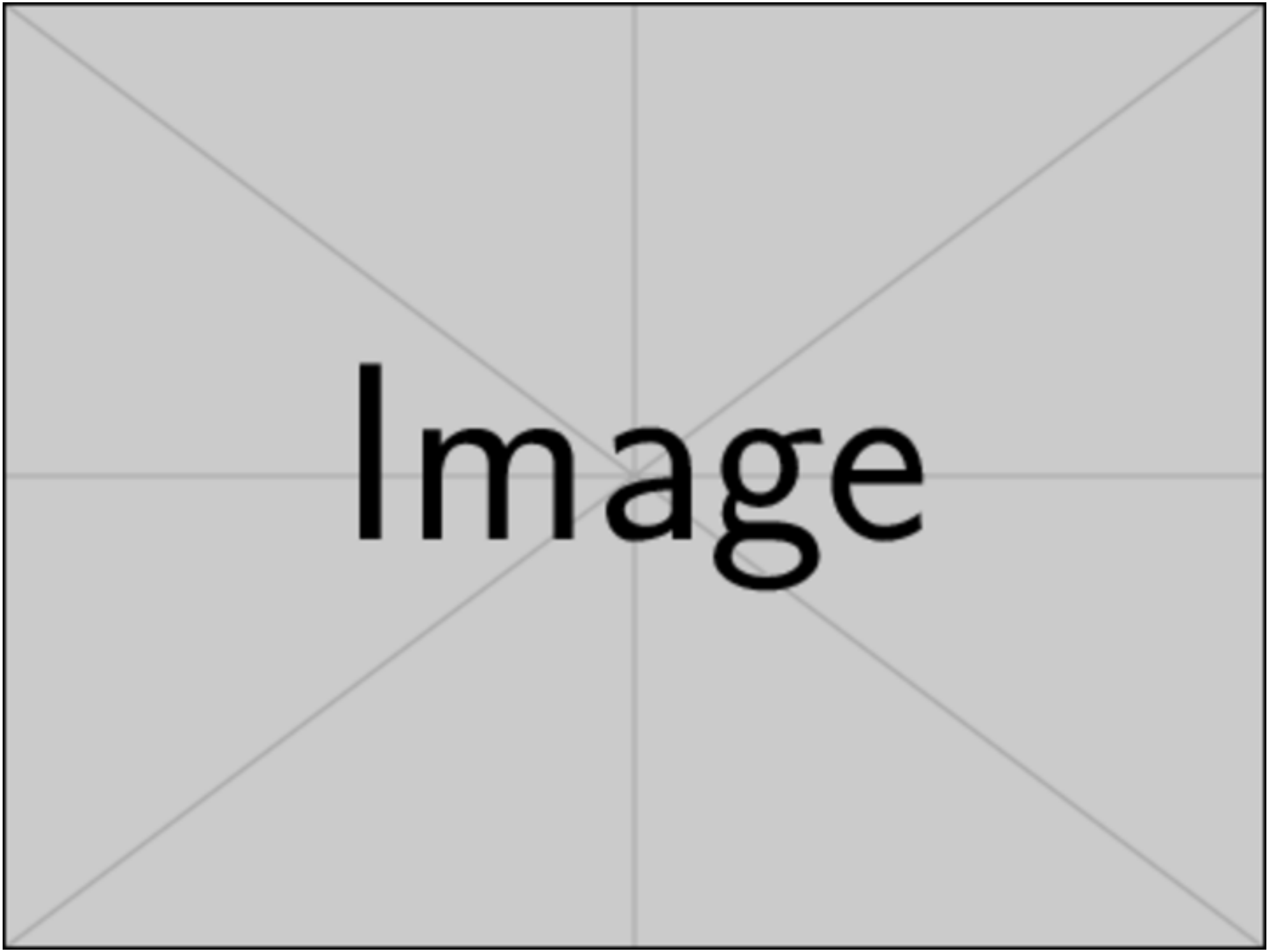

Manuscript

Click here to access/download;Manuscript;HVSeeke-Manuscript.pdf

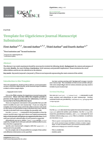

GigaScience, 2023, 1–7

doi: xx.xxxx/xxxx
Manuscript in Preparation
Paper

PAPER

HVSeeker: A Deep Learning-based Method for Identification of Host and Viral DNA sequences

Abdullatif Al-Najim[1,†], Sven Hauns[2,†], Van Dinh Tran[1], Rolf Backofen[2, 3,*] and Omer S. Alkhnbashi[4, 5,*]

[1]Information and Computer Science department, King Fahd University of Petroleum and Minerals, Dhahran, 34462, Saudi Arabia and [2]Bioinformatics group,Department of Computer Science, University of Freiburg,Georges-Köhler-Allee 101,79110,Germany and [3]Signalling Research Centres BIOSS and CIBSS, University of Freiburg, Schänzlestr. 18, 79104 Freiburg, Germany and [4]Center for Applied and Translational Genomics (CATG) and [5]College of Medicine, Mohammed Bin Rashid University of Medicine and Health Sciences, Dubai Healthcare City, Dubai, United Arab Emirates

[*]To whom correspondence should be addressed: omer.alkhnbashi@dubaihealth.ae;backofen@informatik.uni-freiburg.de
[†] shared first authorship

Abstract

Background Bacteriophages are among the most abundant organisms on Earth, significantly impacting ecosystems and human society. The identification of viral sequences, especially novel ones, from mixed metagenomes is a critical first step in analyzing the viral components of host samples. This plays a key role in many downstream tasks. However, this is a challenging task due to their rapid evolution rate. The identification process typically involves two steps: distinguishing viral sequences from the host and identifying if they come from novel viral genomes. Traditional metagenomic techniques that rely on sequence similarity with known entities often fall short, especially when dealing with short or novel genomes. Meanwhile, deep learning has demonstrated its efficacy across various domains, including the Bioinformatics field.
Results We have developed HVSeeker - a host/virus seeker method - based on deep learning for distinguishing between bacterial and phage sequences. HVSeeker consists of two separate models: one analyzing DNA sequences and the other focusing on proteins. In addition to the robust architecture of HVSeeker, three distinct preprocessing methods were introduced to enhance the learning process: padding, contigs assembly, and sliding window. This method has shown promising results on sequences with various lengths, ranging from 200 to 1500 base pairs. Tested on both NCBI and IMGVR databases, HVSeeker outperformed several methods from the literature such as Seeker, Rnn-VirSeeker, DeepVirFinder, and PPR-Meta. Moreover, when compared with other methods on benchmark datasets, HVSeeker has shown better performance, establishing its effectiveness in identifying unknown phage genomes.
Conclusions These results demonstrate the exceptional structure of HVSeeker, which encompasses both the preprocessing methods and the model design. The advancements provided by HVSeeker are significant for identifying viral genomes and developing new therapeutic approaches, such as phage therapy. Therefore, HVSeeker serves as an essential tool in prokaryotic and phage taxonomy, offering a crucial first step toward analyzing the host-viral component of samples by identifying the host and viral sequences in mixed metagenomes.

Key words: Genomics, Bacteria, Phages, Deep Learning

Introduction

Viruses, the most common organisms on the planet [?], significantly affect both ecosystems and human health [?]. Capable of

Compiled on: February 3, 2025.
Draft manuscript prepared by the author.

Key Points

- We introduce HVSeeker , a novel DeepLearning method for classification of bacteria and phage genomes.
- We create three different strategies for creating genomic input sequences and benchmark their effectiveness.
- Data preprocessing with padding achieved better results than using contigs assembly or a sliding window
- HVSeeker compares favorably to alternative classification method, Seeker, Rnn-VirSeeker,DeepVirFinder, PPR-Meta, even on low-homology datasets.
- Additionally we finetune a small ProtBert based model to provide an additional mechanism to evaluate genomic sequences.

infecting a wide range of species, including humans and bacteria, viruses exert a profound effect on bacteria populations. Phages, which specifically target bacteria, have a meaningful impact on their host and are also influencing human health. This underscores the importance of the role that the interaction between bacteria and phages has on humans [?]. Phages infect bacteria by first injecting their viral DNA into the bacteria, after breaking down the cell wall with endolysins. In the next step, the phage DNA either integrates into the bacterial DNA or initiates a lytic cycle, using the bacterial replication instruments to reproduce its DNA [?]. Ultimately, the viral genome and proteins assemble to form a new virion [?].

Due to the constant competition between phages and bacteria, the former have developed defense mechanisms that can be used as an alternative to antibiotics [?]. Furthermore, due to their ability to lyse bacterial cell walls, phage-derived endolysins were suggested as an antimicrobial agent [?]. For that purpose, the phage-bacteria relationship is a hot topic in recent research [?]. Since phages insert their DNA into the bacterial host as part of their replication process, it is crucial to distinguish the phage-derived sequences from the bacterial sequences in any genome found in nature. The detection of phage within the host genome may have a significant impact on studying and understanding such viruses, however, the task is time-consuming and needs extensive lab work [?]. One possible approach to overcome such problems is to analyze metagenomic data that embed virus information as it is shorter than the full genome, ranging from 600 to 25K base pairs (bp) [?], which requires less time and effort. Metagenomics is the study of metagenomes from various environmental samples [?]. It is divided into two main areas: structural and functional metagenomics. Structural metagenomics primarily focuses on gene structure, whereas functional metagenomics examines the functions of genes, specifically the proteins they encode [?]. To classify the origin of a genome found in the environment, sequencing methods can be used. Earlier sequencing methods were based on gene similarity. In other words, it is required for a found genome to be similar to the known virus genome to classify it as an organism of that virus. This works by comparing the found genes to already known genes by creating an alignment. Examples of tools built based on similarity method are Kraken2 [?], Centrifuge [?], and FALCON-meta [?]. However, this method suffers from different shortcomings such as its low ability to discover new viruses since there is no universal viral marker gene exists at the moment [?].

Another proposed method is called binning, which is implemented in tools like MetaWatt [?] and CONCOT [?]. Binning introduces an additional step after the assembly method which groups the given contig into categories that correspond to a biological taxon—a classification used to denote a grouping of organisms, which can range from a single species to broader categories like genus or family, reflecting various levels of the biological hierarchy [?].

In addition, classical sequence comparison approaches such as BLAST [? ?], has been employed (for a discussion, see [?]).

In addition to this algorithmic approaches, researchers have also investigated the use machine learning (ML) approaches for classifying metagenomic sequences, due to the success of ML in various bioinformatics applications. J. Ren et al [?], developed VirFinder, a logistic regression model to identify viral sequences given the genome. The proposed method can identify viruses within sequences of varying lengths, ranging from 500 to 10K bp. Testing VirFinder over NCBI data, however, it was found that VirFinder performs better on larger sequence lengths, which is often unrealistic. Expanding on this work, a new method called DeepVirFinder was introduced [?], serving the same objective as VirFinder. This deep learning approach uses a convolutional neural network (CNN) to identify viral sequences within DNA viral sequences. Unlike the original VirFinder algorithm, DeepVirFinder has been improved to identify viral sequences with shorter lengths, specifically between 150 and 3000 bp. Additionally, this method is capable of identifying viral sequences in real human gut metagenomic samples.

There are several other deep-learning based models employing different neuronal architectures for metagenomics based task. Thus, Seeker [?] solves the problem of differentiating phage sequences from bacterial ones and is based on long-short-term memory (LSTM) architecture. F. Liu et al [?] proposed RNN-VirSeeker, a deep-learning method for viral sequence identification also utilizing LSTMs and outperforms other state-of-the-art methods. PPR-META [?] is a deep learning based method that classifies contigs into phage, plasmid, and chromosome categories. The method was tested on artificial and real genomes of different lengths, ranging from 100 to 10,000 bp, and successfully outperformed other state-of-art methods. Finally, VIDHOP [?] is a method that can identify the original host of the virus in addition to the potential host genome. Two different deep learning methods were proposed the first one was based on LSTM, while the other one combines LSTM with CNN.

Despite these advances, previous state-of-the-art methods often underperform, particularly in identifying new genomes, as we demonstrate in our benchmark based on a viral metagenomic dataset from infant guts. Additionally, authors fail to provide a method for cross-verifying classifications based on DNA sequences with those based on proteins derived from these DNA sequences. Our tool outperforms previous approaches in the mentioned benchmark and also introduces a method to classify protein sequences, which vastly outperforms hidden markov model (HMM) based methods.

This study proposes a deep learning-based method, HVSeeker, to enhance the identification of host and viral sequences in metagenomic data. We developed a robust approach for HVSeeker by testing different preprocessing methods and validating the performance across different degrees of homology. Combined with models architectures particular sensitive to sequence data, this allows us to create an approach that outperforms previous models. Experimental results demonstrate that HVSeeker can accurately identify both short and long host and viral sequences in metagenomes, outperforming a variety of widely used methods: Seeker, Rnn-VirSeeker, DeepVirFinder, and PPR-Meta.

Methods

Data Collection & Description

The research data for this study was gathered from well-known bioinformatics databases, including the National Center for Biotechnology Information (NCBI) [?] and the Integrated Microbial Genomes & Microbiomes - Viruses (IMGVR) [? ?]. The data consists of Bacterial and Phage DNA sequences. Each file contains a complete genome of different lengths and some metadata, e.g. the unique ID for each sequence and its length. The full dataset comprises 536 and 2687 bacterial and phage sequences, respectively.

Data Preprocessing

The initial dataset consists of various DNA sequences encapsulated within Fasta files. These sequences vary in both biological origins and lengths. A preliminary task in our methodology involves the extraction of proteins from these diverse DNA sequences, which correspond to multiple classes. Thus, the input data for the subsequent training models constitute both DNA and protein sequences.

To ensure consistency in our data processing, we adjust the length of each DNA sequence to a uniform standard. In this process, we split the entire genome into segments of 1000 base pairs (bp), a procedure that often results in sequences shorter than the designated length as the genomes are usually not a multiple of our subsequence length (i.e. 1000bp).

To address this issue, we employ three distinct strategies. The first strategy involves sequence padding, wherein we repetitively cycle through the sequence until it attains the required length. When padding sequences that are naturally shorter than the maximum number of base pairs, they are extended by duplicating their own sequence, therefore completing the short sequence by repeating it in the same order. The second strategy, which we termed contigs assembly approach, is a two-fold process: we initially combine multiple shorter left-over sequences to generate a new, longer sequence and split it into subsequences of our chosen length. Subsequently we apply padding to any residual segment that hasn't been incorporated into another sequence. The last strategy is a sliding-window process. In other words, for each sequence, we will select the first 1000 bp, and continue the processes by moving the window by 100bp until the end of the DNA. These techniques allow us to process input data of arbitrary length.

Upon achieving DNA sequences of uniform length, we proceed to eliminate any exact duplicate sequences.

Subsequently, we employ one-hot encoding to transform the contigs from their original nucleotide form (adenine, cytosine, guanine, thymine, or ACGT) to a binary matrix of 0s and 1s. This conversion facilitates easier processing and interpretation of the genetic data by our computational model.

In the final preprocessing step, we adopt an undersampling approach to balance the classes in our dataset. This procedure ensures that our model does not exhibit bias towards any particular class, leading to more reliable and generalizable predictions. With these steps, we conclude the preprocessing of the genomic sequence data. Following preprocessing, the dataset increased to 565,760 DNA sequences. For the model training process, we employed the holdout method, a recommended approach for larger datasets. The allocation of the data was set at 80% for the training set, with the remaining 20% equally split between validation and testing, at 10% each. To prevent overfitting, we employed an early-stopping technique, which terminates the training when the performance on the validation set degrades. The below points provide a summary of the preprocessing methodology adopted in this research. Additionally, a visual summary of the same can be found in Figures ??, ??, and ??.

Our model for classifying proteins, based on their amino acid sequences, uses a dataset comprising 98720 unique phage sequences and 122366 unique bacterial sequences. To ensure a broad representation of protein diversity, we employ BLAST to limit sequence homology between sets. This approach allows us to effectively capture the wide range of diversity found in bacterial and phage proteins. For testing purposes, we divided the data into 80% training data and 20% test data and repeated this process 5 times.

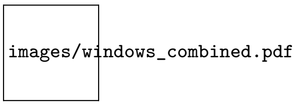

Figure 1. A) Data preprocessing with padding. We cycle through the sequence until it attains the required length. B) Data preprocessing with contigs assembly method. Initially, we combine multiple shorter sequences to generate a longer sequence, which is then split again into subsequences of length 1000bp. Finally, we apply padding to any residual sequence. C) Data preprocessing with 1000/100 bp sliding window method. We use a window of 1000 bp size to slide over the input DNA sequence in 100 bp steps.

These steps encapsulate the preprocessing stage of our research which will create two datasets, one for HVSeeker-DNA and another for HVSeeker-Protein:

- Extraction of proteins from DNA sequences.
- Standardization of DNA sequence lengths to 1000 bp.
- Handling of shorter sequences via padding, a combination of multiple sequences, or a sliding-window process.
- Removal of duplicate sequences.
- Transformation of nucleotide sequences to a binary matrix using one-hot encoding.
- Class balance in the dataset achieved through undersampling.

The proposed models

To leverage the availability of both DNA and protein sequences, we develop two distinguished models. The first model, called HVSeeker-DNA, is an LSTM-based model that takes as input DNA sequences with a length of 1000 bps. LSTMs are primarily designed for sequential data where each input influences subsequent or preceding entries. Similarly, in DNA sequences, each nucleotide is related to its context, providing distinct meaning compared to individual elements. Therefore, we consider LSTMs to be an optimal choice for addressing DNA-related challenges. For the second one, HVSeeker-Protein, we adopt the ProteinBERT [?] as a pre-train model and perform a fine-tuning phase using the constructed protein sequences. ProteinBERT has been trained on diverse protein sequence corpus in a self-supervised manner. Therefore, it allows to have a generic representation for every input protein sequence. In the following, each model will be described in detail.

HVSeeker-DNA structure

HVSeeker-DNA consists of three bidirectional connected LSTM units, followed by two fully connected layers, and then a softmax activation function for the final prediction. The first LSTM unit reads each data entry as a 6×1000 matrix due to the one-hot encoding process, then it will output a vector of length 150 to be passed to the next two LSTM models. Then, a fully connected layer with the elu activation function and dropout of 0.2 will read the output of the last LSTM model and pass it to the next fully connected layer before using the softmax activation function for the final prediction. A visual summary of the model is presented in Figure 2

Figure 2. HVSeeker-DNA Architecture. First, the DNA data is encoded using different principles (padding, contigs-assembly, sliding window). We then process the one-hot encoded DNA using 3 bidirectional LSTM layers, followed by a linear layer with a moderate amount of dropout (0.2) and an elu activation function, and an output layer.

HVSeeker-Protein structure

In the event that the classification provided by the first model requires support with additional information about the proteins expressed by the DNA, we offer a second model that relies solely on the expressed protein sequences. To classify proteins as either phage or bacterial, we utilize embeddings generated by ProteinBERT. These embeddings accurately represent the input proteins, thanks to the extensive dataset of training proteins used in ProteinBERT [?]. The model's architecture includes a transformer with four attention heads and six layers, featuring a key size of 64. It has been trained on a large dataset of 160 million protein sequences, focusing on a reconstruction task. To enhance the finetuned model's precision in predicting protein types, we employ a Bayesian optimizer. This optimizer uses an expected improvement acquisition function, initiating with eight random starts and conducting 25 evaluations on 1000 proteins sampled from the training set. These evaluations fine-tune the learning rate, the number of training epochs, and the learning rate decay factor, optimizing the model's performance.

To evaluate the effectiveness of the optimization and fine-tuning process, we conduct an assessment using 5-fold cross-validation for the optimization process for five runs. For each test set, we use BLAST to ensure that sequence homology between the training and test set does not exceed 0.95. By employing this method, we can rigorously test the model across different subsets of the data, guaranteeing that our assessment of its performance is both fair and reliable.

Evaluation Criteria

To evaluate the effectiveness of the proposed models we focus on four key metrics: Accuracy, Precision, Recall, and F-1 score.

To evaluate model performance accuracy

$$Accuracy = \frac{TP + TN}{TP + FP + TN + FN} \tag{1}$$

is commonly used to calculate the ratio of correctly predicted instances to the total number of predictions.

However, accuracy can be misleading in the case of imbalanced datasets. Therefore, relying solely on accuracy is insufficient, and other metrics must be considered.

When wanting to focus on the positive class, precision

$$Precision = \frac{TP}{TP + FP} \tag{2}$$

can be used to measures the proportion of correctly predicted positive instances relative to all predicted positives.

Measuring the proportion of correctly predicted positive instances out of actual positives, recall

$$Recall = \frac{TP}{TP + FN} \tag{3}$$

is a crucial metric for addressing the impact of model bias in imbalanced datasets.

To balance precision and recall, the F1 measure

$$F1\ measure = 2 \times \frac{Precision \times Recall}{Precision + Recall} \tag{4}$$

provides a robust metric, offering a more comprehensive view of a model's predictive strength.

Results & Discussion

To assess the effectiveness of the algorithm, four experiments were designed and executed. In the first experiment, we trained the model across a variety of sequence lengths to determine the optimal length for Bacteriophage prediction. In the second experiment, we performed a self-comparison of our model under three distinct preprocessing conditions to understand the impact of these variations on the model's performance. The third experiment compared our model with other models reported in the literature, on unseen data sequences from the same environment as our training set. The last experiment involved benchmarking our model against others in the literature using a standardized dataset. All the experiments are further discussed in the following sections.

Optimizing Sequence Length for Improved Prediction Performance

To find the best sequence length for Bacteriophage prediction and to assess model performance across different sequence lengths, we trained our model using sequences of 2000, 1500, 1000, 500, 200, and 100 base pairs. We used padding as a preprocessing method for shorter sequences to ensure consistent input lengths. Figure 3 displays a comparative analysis of model performance, presenting Precision, Recall, Accuracy, and F1-score for each evaluated sequence length. Figure 3 shows that the model trained on 2000 bp sequences consistently predicted the bacteria class, indicating potential overfitting and an inability to generalize. This could be due to the longer sequence lengths introducing noise. On the other hand, the model trained on 1000 bp sequences showed the best performance in terms of F1-score, outperforming other models with its Recall rate, despite the 1500 bp model achieving slightly higher Precision. Conversely, the model with 100 bp sequences underperformed, as expected due to the reduced informational content of shorter sequences for Bacteriophage identification. The findings also indicate that sequence lengths between 1500-2000 bp resulted in similar accuracy levels. This suggests that there is a threshold beyond which shorter sequence lengths start to noticeably affect model performance. Based on these results, subsequent experiments focused solely on the 1000 bp sequence length, which demonstrated the most balanced performance across all evaluated metrics.

images/DNA_Sequence_Prediction_Experiments_Comp

Figure 3. Comparison on different sequence lengths in terms of Precision, Recall, F1-score, and Accuracy. We see the best performance in terms of the F1-score when using a length of 1000 bp and the best performance in terms of recall for a length of 1500 bp. Length 2000 bp achieved a score 0 for all metrics except for accuracy due to overfitting. Here, HVSeeker potentially fails to capture local sequence properties as effectively as with smaller sequence sizes. Overall, we can conclude that excessively large sequence lengths overfit, while overly small sequence lengths underperformed.

Evaluating the Impact of Preprocessing Techniques on Model Accuracy

In section , we introduce three preprocessing methods of the data: padding method, assembly of shorter sequences method, and sliding window method. To assess the impact of each preprocessing technique, we trained a separate model for each method. The train-

ing and validation accuracies of these models are shown in Figures 4, 5 respectively. According to these figures, the sliding window method initially led to higher accuracy during the early training epochs. However, as training progressed, all three models converged to comparable levels of accuracy for both the training and validation phases. Despite the overall similarity in performance, a closer inspection reveals that the padding method slightly outperformed the others in terms of validation accuracy, whereas the assembly of contigs method was slightly behind. This could be attributed to the padding method's tendency to duplicate nucleotides, potentially providing the model with more consistent training data. In contrast, the assembly method, which might combine sequences from varied origins, could introduce a higher degree of variability and confusion to the model's learning process. Eventually, the three models achieved validation accuracy exceeding 80% which indicates a robust model architecture capable of adapting to various preprocessing strategies. Another interesting observation is that all three methods achieved similar scores across all metrics, unlike others that may be biased towards recall or precision, like DeepVirFinder or PPR-Meta. This consistency could be attributed to the balanced dataset used for training HVSeeker, highlighting the importance of the undersampling step in preprocessing. Additionally, this demonstrates the robustness of HVSeeker's preprocessing and architecture, achieving an excellent balance between generalization and adapting to the training data.

images/train_acc_size_20.png

Figure 4. Training accuracy of the three models. All models converge relatively quickly to their maximal performance with small differences between the accuracies of the embedding methods.

images/val_acc_size_20.png

Figure 5. Validation accuracy of the three models. All models converge relatively quickly to their maximal performance with small differences between the accuracies of the embedding methods.

Benchmarking the Proposed Method Against Existing Tools

To extend our evaluation of the proposed models, we compared them with existing bacteriophage classification approaches found in the literature, specifically Seeker [?], Rnn-VirSeeker [?], DeepVirFinder [?], and PPR-Meta [?]. For a fair comparison, we ensured that each algorithm was trained using the same dataset introduced in our study. Table 1 outlines the final results of each model on unseen testing data. As shown in the table, the three proposed models outperformed both Seeker and Rnn-VirSeeker in terms of Precision, Recall, Accuracy, and F1 Score. While both DeepVirFinder and PPR-Meta achieved higher precision score, they achieved lower Recall, Accuracy, and F1 score. Notably, all the models posted comparable results across various evaluation metrics, suggesting that our model construction was robust enough to mitigate potential biases. Conversely, Seeker's model yielded an F1 score of approximately 58%, hinting at a need for more extensive data to enhance its performance. In contrast, Rnn-VirSeeker appeared to struggle with underfitting, being unable to adapt to the complexity of the training data, it only achieved an accuracy of roughly 50% on the training data. This underperformance could stem from the label encoding technique RNN-Seeker employed for encoding their data, which assigns numerical values to nucleotides. This method is generally not recommended because it can prioritize some features over others and suggests an order between nucleotides, generally not found in the natural world, which potentially skews the data analysis and interpretation. In the case of DeepVirFinder and PPR-Meta, these models exhibit high precision but low recall. This indicates that while they are correctly identifying phages, many actual phage genomes remain undetected, thus lacking comprehensive accuracy and F1 score in phage prediction.

	Precision	Recall	Accuracy	F1-Score
Padding	83.72%	**90.07%**	**87.25%**	**86.78%**
Contigs-assembly	80.76%	80.02%	80.30%	80.39%
Sliding-window	83.40%	82.43%	82.81%	82.91%
Seeker	56.98%	58.95%	59.39%	57.95%
Rnn-VirSeeker	00.00%	00.00%	50.00%	00.00%
DeepVirFinder	90.50%	65.14%	79.23%	75.75%
PPR-Meta	**92.00%**	67.00%	81.00%	77.00%

Table 1. Comparison of the proposed approach with different preprocessing methods against Seeker, Rnn-seeker DeepVirFinder, and PPR-Meta. We find that our method outperforms others in most of the used metrics, specifically in Accuracy and F1 score, regardless of the input embedding used. Both using padding and a sliding-window approach result in similar good results, with both methods showing a slight advantage over using contigs-assembly.

Performance on low-homology datasets

To challenge the generalization ability of our model, we use our previously created test set and remove all sequences above a certain homology cutoff by using BLAST to compare the sequences to our training set. Based on the resulting alignment files, we create five different homology datasets. The results for all datasets can be found in supplementary figure S3-S7. HVSeeker consistently achieves the best F1-Score for all data splits. When using a homology cutoff of 60%, HVSeeker still achieves an F1-Score of 68.60% with a precision of 57.70% and a recall of 84.80%. Other methods usually do not balance precision and recall as well, resulting in a considerably lower F1-Score.

	Precision	Recall	Accuracy	F1-Score
HVSeeker	57.70%	84.80%	70.30%	**68.60%**
Seeker	38.29%	**98.51%**	38.57%	55.15%
Rnn-VirSeeker	00.00%	00.00%	62.00%	00.00%
DeepVirFinder	63.33%	51.93%	70.03%	57.06%
PPR-Meta	**78.00%**	44.00%	**74.00%**	56.00%

Table 2. Comparsion of the proposed approach with previous methods against Seeker, Rnn-seeker DeepVirFinder, and PPR-Meta on a low-homology dataset of 60% maximum homology. We find HVSeeker achieves the highest F1-Score with 68.60%, roughly 10% better than the second best method DeepVirFinder with 57.06%. Additionally used low-homology sets can also be found in the supplementary under Table S3-S7.

Performance on a diverse viral metagenomic dataset from infant guts metagenomic dataset

A key challenge in bacteriophage detection is the recognition of new sequence patterns. To evaluate our algorithm's capability in this regard, we conducted a performance comparison using benchmark datasets collected from [?] with Seeker, Rnn-VirSeeker, Deep-

VirFinder, and PPR-Meta. The study analyzed viral diversity in the fecal viromes of 647 one-year-olds from the Copenhagen Prospective Studies on Asthma in Childhood 2010 (COPSAC2010). Fecal samples were successfully collected and viromes were characterized for 647 children at one year, with metagenomes sequenced in parallel. The study's authors identified 10,000 viral species from 248 virus family-level clades, with 232 being newly discovered, primarily from the Caudoviricetes class. Hosts for 79% of the phages were determined using CRISPR spacers from bacterial metagenomes of the same children. The results of our comparison are detailed in Table 3. The proposed algorithm consistently outperformed all methods across all evaluated metrics except accuracy. Notably, our algorithm demonstrated a greater recall relative to precision, which can be attributed to the benchmark dataset's composition—featuring a higher count of negative than positive instances. With a final F1 Score of 0.767, closely mirroring its performance on testing data, our model proves its proficiency in accurately processing sequences from varied environments, comparably to how it performs with the training set's environment. Conversely, Seeker, with an F1 Score of 0.578, DeepVirFinder with an F1 score of 0.417, and PPR-Meta with 44, adapt to the data during training only to a limited extent, suggesting either insufficient data or inadequate model complexity. Meanwhile, Rnn-VirSeeker exhibited underfitting, indicating a failure to learn effectively from the training data.

	Precision	Recall	Accuracy	F1-Score
HVSeeker	**67.01%**	**89.74%**	65.23%	**76.73%**
Seeker	42.92%	88.43%	46.40%	57.79%
Rnn-VirSeeker	00.00%	00.00%	14.46%	00.00%
DeepVirFinder	32.18%	59.04%	74.22%	41.66%
PPR-Meta	34.00%	61.00%	**77.00%**	44.00%

Table 3. Comparison on benchmark data. Again HVSeeker outperforms the alternative method Seeker by an impressive margin of 18.9 for the F1-Score and 18.83 in terms of the classification accuracy. Meanwhile, Rnn-VirSeeker seems to be unable to learn properly from the provided data.

Protein based classification

We report the average performance over all five runs of our method assessed on our test-sets, achieving an average AUC value of 0.89. This indicates a strong performance in the protein-based classification task. Additionally, the classification accuracy across all splits is 82%, further demonstrating the method's effectiveness. The weighted F1-score stands at 0.82, with a weighted precision of 0.82 and a weighted recall of 0.82. The additional information created by this classification mechanism can be used reliably to support the prediction created purely based on the DNA. For the minority of the sequences of a length from 571 to 1144 we find a similar AUC value of 0.88, indicating the stability of the results for greater sequence lengths. The weighted F1-score stands at 0.84, with a weighted precision of 0.84 and a weighted recall of 0.84. To compare our method with a baseline approach, we constructed Hidden Markov Models (HMMs) for all five subsets of the training dataset. Initially, we generated multiple sequence alignments using MAFFT (FFT-NS-2) [?], followed by the construction of HMMs with HMMbuild [?]. The subsequent search over the test data yielded only 12 correct hits above the standard reporting threshold. This result indicates the model's inability to adequately represent the diversity of the input data, translating to an accuracy of approximately 0.0051%.

Conclusion

In this study, we introduced HVSeeker, a novel methodology designed to differentiate between viral and bacterial genomic sequences. HVSeeker surpasses the existing state-of-the-art methods, DeepVirFinder, PPR-Meta, RNN-VirSeeker, and Seeker, in performance on two benchmarks and additionally evaluates corresponding proteins, offering improved insights. Unlike previous methods HVSeeker can work with DNA input as well as protein inputs, allowing researchers to combine evidence for both. This advancement is important for accurately identifying viral genomes and therefore the creation of new therapeutic approaches such as phage therapy. Identifying the host and viral sequences in mixed metagenomes is the initial step toward analyzing the host viral component of samples. This process is crucial for downstream work. HVseeker proves to be an essential tool in prokaryotic and phage taxonomy, as well as in bacterial-host interactions.

Availability of source code and requirements

- Project name: HVSeeker [?]
- Project home page: `https://github.com/BackofenLab/HVSeeker`
- Programming language: Python
- Other requirements: To install software requirements we provide a conda environment file for HVSeeker-DNA and HVSeeker-Protein in the github repo
- License: MIT licence
- in bio.tools registered as hvseeker
- rrid: SCR_026120
- An archival copy of the github repository is available via software heritage [?]
- see DOME-ML questionary for more details [?]

Data availability

The used model is available under https://github.com/BackofenLab/HVSeeker [?].DOME-ML annotations are available vie the DOME registry under accession igr5x3a1vs [?].

Additional Files

- Supplementary table S1: Comperative performance metrics of sequence identification methods on test set
- Supplementary table S2: Performance metrics of HVSeeker, Seeker, and Rnn-VirSeeker on unseen benchmark dataset
- Supplementary table S3: Comparative performance metrics of sequence identification methods on test dataset with a maximum of 95% homology
- Supplementary table S4: Comparative performance metrics of sequence identification methods on test dataset with a maximum of 90% homology
- Supplementary table S5: Comparative performance metrics of sequence identification methods on test dataset with a maximum of 80% homology
- Supplementary table S6: Comparative performance metrics of sequence identification methods on test dataset with a maximum of 70% homology
- Supplementary table S7: Comparative performance metrics of sequence identification methods on test dataset with a maximum of 60% homology

Declarations

List of abbreviations

- LSTM: Long-short-term-memory.
- bp: base-pairs
- ML: machine-learning
- Pfam: protein family
- CNN: convolutional neural network
- HMM: hidden markov model
- NCBI: National Center for Biotechnology Information
- IMGVR: Integrated Microbial Genomes & Microbiomes - Viruses

Consent for publication

Not applicable

Competing Interests

The author(s) declare that they have no competing interests.

Funding

Deutsche Forschungsgemeinschaft Grant, BA 2168/23, Much more than Defence: the Multiple Functions and Facets of CRISPR–Cas. And Deutsche Forschungsgemeinschaft, Grant, BA 2168/25-1, Einluss von RNA-bindenden Proteinen und mRNA-Strukturen auf alternative Translation-Regulationsmechanismen im entzündlichen Tumorgeschehen. The article processing charge is funded by the Baden-Wuerttemberg Ministry of Science, Research and Art and the University of Freiburg in the funding programme Open Access Publishing

Author's Contributions

Al-Najim and S.H. developed the software. Al-Najim, S.H. and O.S.A wrote the initial draft of the manuscript. O.S.A. conceived the study and analyses. O.S.A and V.D.T. data acquisition. O.S.A., and R.B. oversaw the project. All authors reviewed, contributed to, and approved the manuscript.

Acknowledgements

The authors acknowledge support by the High Performance and Cloud Computing Group at the Zentrum für Datenverarbeitung of the University of Tübingen, the state of Baden-Württemberg through bwHPC and the German Research Foundation (DFG) through grant no INST 37/935-1 FUGG. The authors also would like to thank King Fahd University of Petroleum and Minerals (KFUMP), Mohammed Bin Rashid University of Medicine and Health Sciences (MBRU), and the reviewers for their valuable suggestions.

Click here to access/download;Figure;dna_model2.png

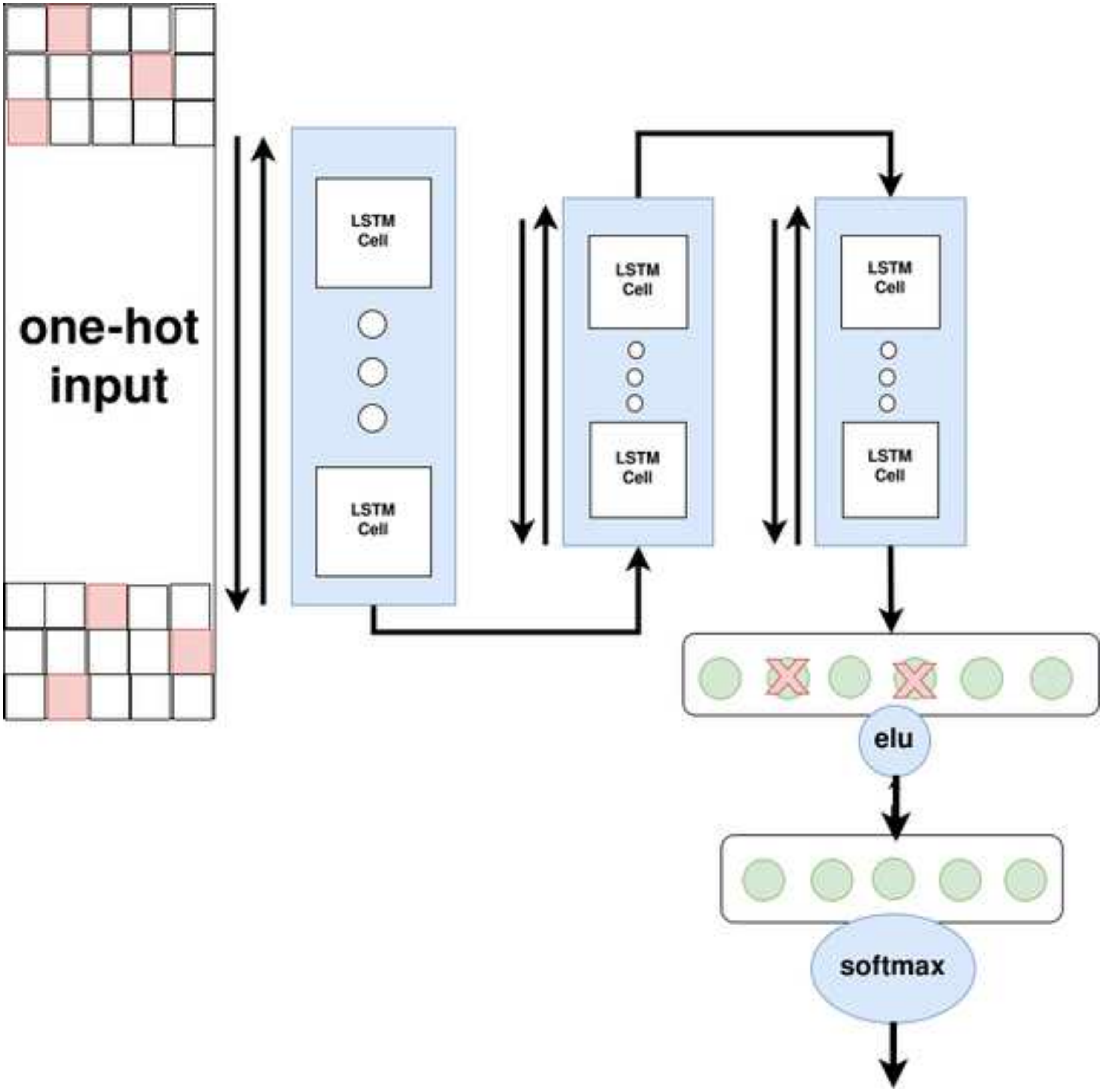

Click here to access/download;Figure;DNA_Sequence_Prediction_E

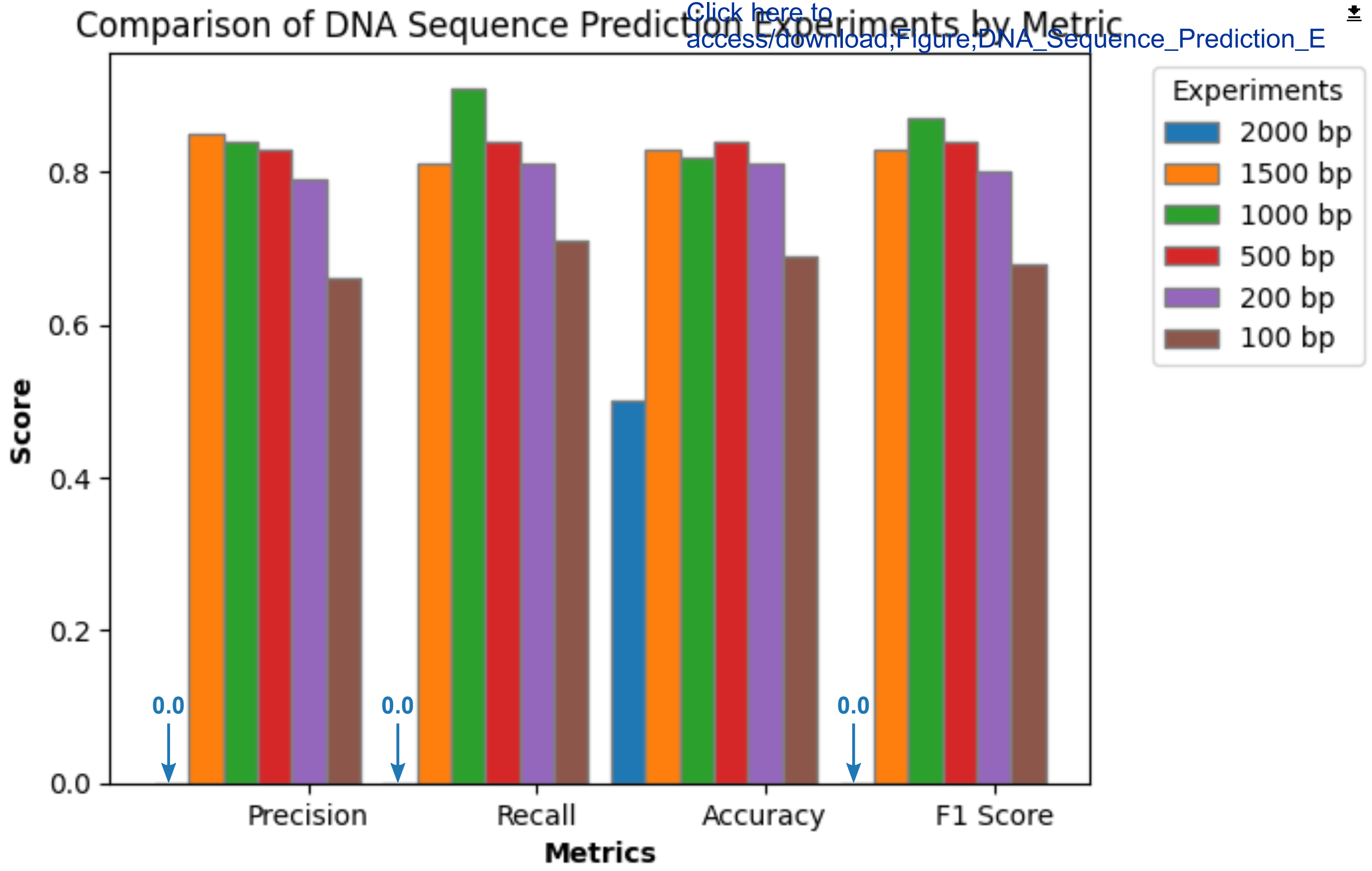

Click here to access/download;Figure;train_acc_size_20.png

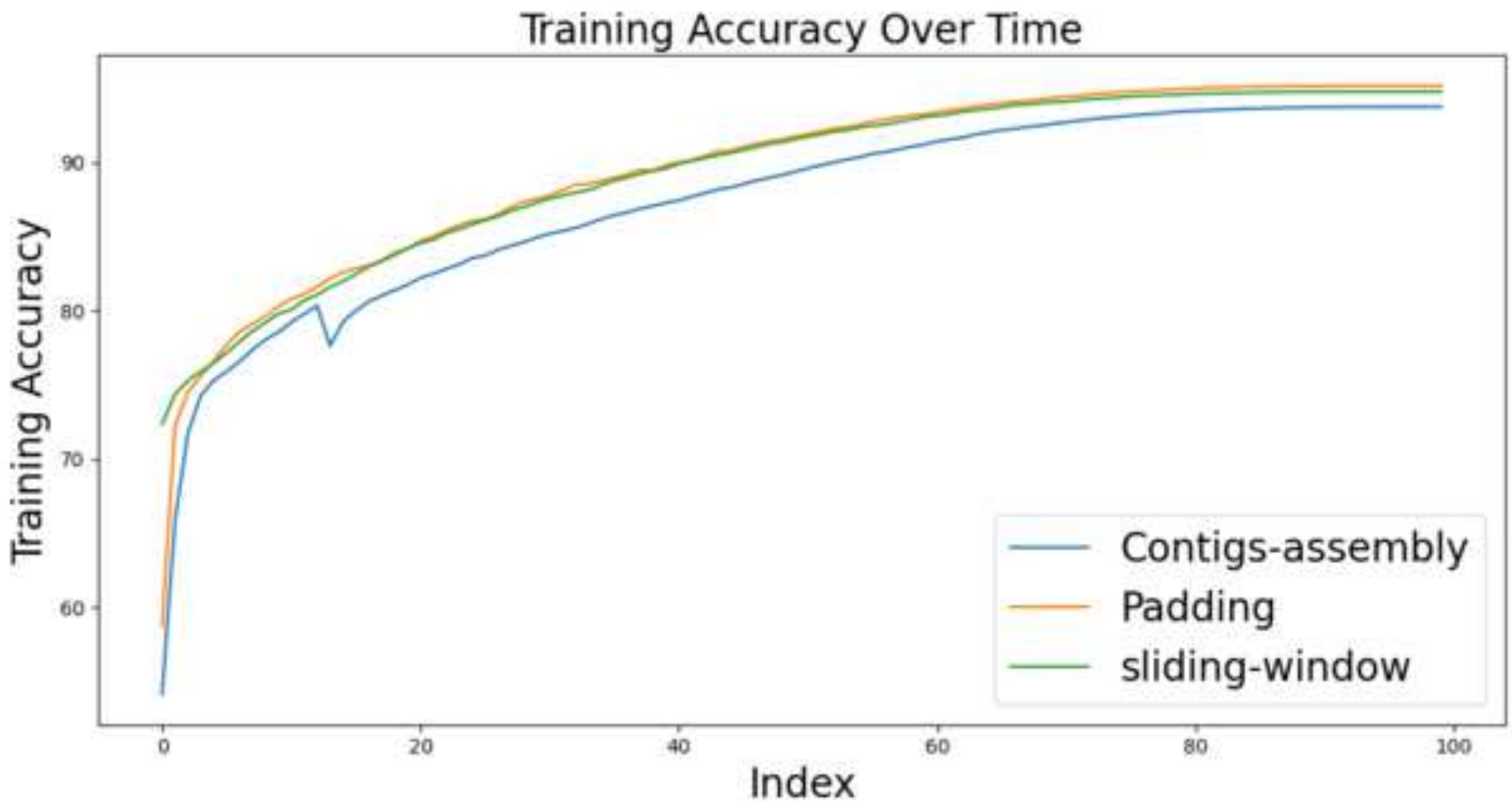

Click here to access/download;Figure;val_acc_size_20.png

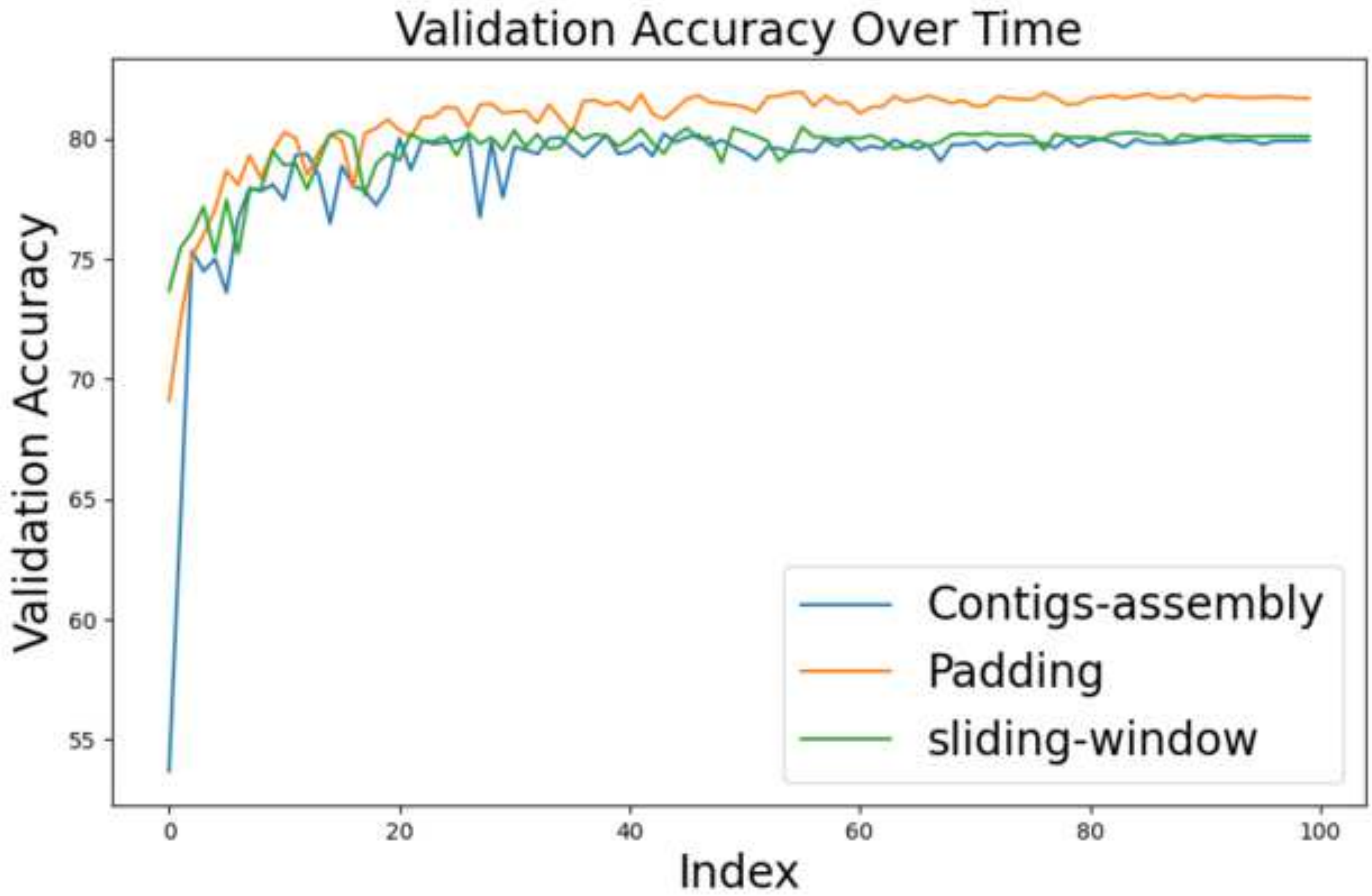

Click here to access/download;Figure;windows_combined.p

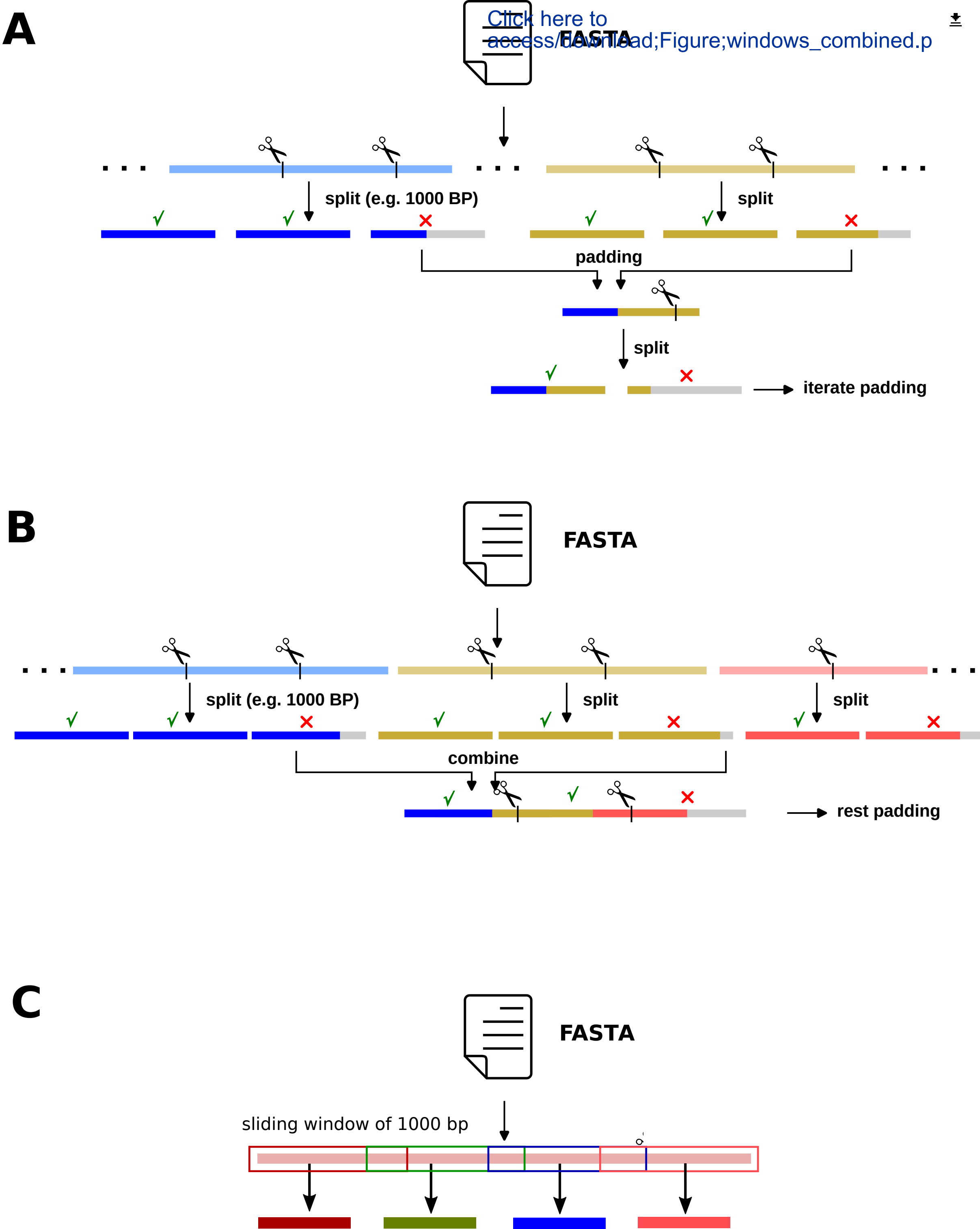

Click here to access/download;Figure;

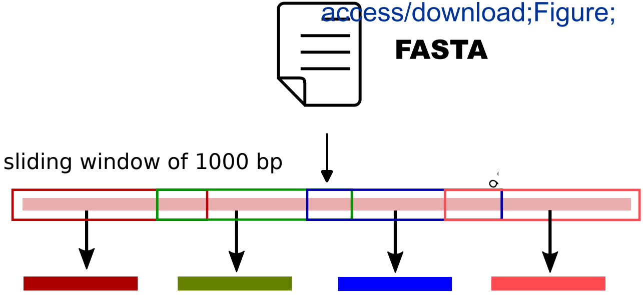

Click here to access/download
Supplementary Material
HVSeeke_Supplementary.pdf

Click here to access/download
Supplementary Material
windows_combined.svg

Click here to access/download
Supplementary Material
dna_model2.svg

Click here to access/download
Supplementary Material
DNA_Sequence_Prediction_Experiments_Comparison_
Metrics-rolf.svg

Dear Dr. Hans
Editor-in-Chief of GiGascience

Herewith we submit the revised version of our manuscript “HVSeeker: A Deep Learning-based Method for Identification of Host and Viral DNA sequences”

We have carefully addressed all the comments and suggestions provided by the journal and integrated them into a thoroughly revised manuscript version.

Additionally, we would like to take this opportunity to thank you, the reviewers, and the editorial team for the acceptance of our paper. We greatly appreciate the constructive feedback and guidance throughout the review process, which has significantly contributed to improving our manuscript. Our response to each comment below is in blue.

I am looking forward to hearing from you.

Sincerely yours,

Dr. Omer Alkhnbashi

Point-to-point response to reviewer comments

- In the data availability section, you write "The data belonging to the study can be found in the GigaDB of the publication." - As far as I understood, our curators didn't feel it was necessary to prepare a GigaDB set, as the data and code are available form other sources (e.g. Software Heritage) - if that's the case, you can delete this sentence.

Author's response: Done

- Also in the data availability section, you write "The used model is available under https://github.com/BackofenLab/HVSeeker" - please also cite the URL here, i.e " "The used model is available via the github repository [31]"

Author's response: Done

- In the Data availability section, please also include the sentence "DOME-ML annotations are available vie the DOME registry under accession igr5x3a1vs [33]"

Author's response: Done

- Importantly, at this stage, we need editable files (word or LaTex source code and bib/style files) - please upload them to EM in addition to an converted PDF.

Author's response: Done
