# Supplementary material for: HVSeeker: a deep-learning-based method for identification of host and viral DNA sequences
Source: Gigascience. 2025 May 15;14:giaf037. doi: 10.1093/gigascience/giaf037 (PMC12080225; doi:10.1093/gigascience/giaf037)
Supplement: giaf037_Authors_Response_To_Reviewer_Comments_Original_Submission [file giaf037_authors_response_to_reviewer_comments_original_submission.pdf]

Author's Response To Reviewer Comments

Dear Dr. Hans
Editor-in-Chief of GiGascience

Herewith we submit the revised version of our manuscript “HVSeeker: A Deep Learning-based Method for Identification of Host and Viral DNA sequences”.

We have answered each point and have modified our submission accordingly. We have also carefully considered the reviewer’s comments and integrated them into a thoroughly revised manuscript version.

We thank the reviewers for their supportive comments and constructive feedback and the editor for the opportunity to submit a revised manuscript. Our response to each comment below is in blue, while the corresponding changes to the main text are in green.

I am looking forward to hearing from you.

Sincerely yours,
Dr. Omer Alkhnbashi

Point-to-point response to reviewer comments

Reviewer #1:
This article presents a well-structured and comprehensive study on HVSeeker, a novel deep learning method for classifying bacterial and phage genomes (for DNA and protein sequences). The work is timely and relevant, offering a valuable contribution to the field. However, there are some points that require attention.

Major points:

- Prophages are bacterial viruses that have integrated their DNA into the genome of a host bacterium. The article does not discuss the impact of prophages in the classification, specially in the metagenomics context. Since prophages are relevant to the research context, it would be beneficial to at least do one of these two: a) include prophage analysis: integrate an analysis of prophages in the study to ensure comprehensive genomic and phenotypic insights; b) acknowledge prophage influence: discuss how prophages might influence the results and interpretations of the study, particularly if they are known to play a significant role in the bacterial strains under investigation.

Author’s response: We thank the reviewer for his comment. We intend to add this function to HVSeeker, but the phaster server (https://phastest.ca/submissions/new) is currently under maintenance. We will update the script accordingly as soon as possible.

- The following sentence is confusing: "the functions of genes, specifically the proteins they encode [9]. To determine whether an organism be longs to a species characterized by a genome, sequencing methods can be used. Earlier sequencing methods were based on gene similarity. In other words, it is required for a provided genome to be similar to the known virus genome to classify it as an organism of that virus.". It seems that sequencing and reconstruction/classification is somewhat mixed. Please, clarify.

Author’s response: We thank the reviewer for their insight and have clarified the text accordingly.

- The paper does not provide sufficient details about the dataset's diversity, such as the sources and environments of the samples. Additionally, please clarify what removal of duplicate sequences mean (exact duplications?).

Author’s response: We thank the reviewer for the comment. To ensure diversity of our dataset we analyzed the similarity of the sequences used and ensured the generalization of the model to new sequences by testing on low-homology samples. The details are explained in the supplementary. In the first step we only remove exact duplicates, but address different homology levels later in the study to gain further insight.

- Given the performance reported, especially on validation datasets, there might be concerns about overfitting. The paper poorly mention techniques used to prevent overfitting, such as data augmentation or early stopping criteria.

Author’s response: Thank you for your comment. As suggested we improved the paper by adding a paragraph detailing methods used to prevent overfitting. Additionally we tested the generalization of our models by providing test statistics on sequences that are very much unlike our training data. Testing on such low-homology sequences shows that our model does not overfit on the provided training data.

- The 2000bp provided a f1-score of 0. Please better explain this: "Figure 5 shows that the model trained on 2000 bp sequences consistently predicted the bacteria class, indicating potential overfitting and an inability to generalize".

Author’s response: Thank you for your comment. It seems that HVSeeker fails to generalize to the test data for very large sequences, potentially due to failing to represent local sequence properties as well as smaller sequence sizes. To remedy that we would need to inflate both our model and our training data. We have clarified this point in the corresponding figure.

- Please, clarify: "Rnn-VirSeeker appeared to struggle with underfitting, showing a preference for the Phage class".

Author’s response: Thank you for the insight. We clarified the point in the paper.

- About the implementation: I was unable to test HVSeeker. To use HVSeeker many installations are required. Please, provide a container for proper testing.

Author’s response: Thank you for the comment. We added a docker container to the github to facilitate testing.

Moderate points:

- The following sentence: "An example of tools that are built on the binning method is MetaWatt [12] and CONCOT [13]." is in some way repeated (this information was provided before).

Author’s response: Thank you for the comment. We improved the paper accordingly.

- "When padding sequences that are naturally shorter than the maximum number of base pairs, they are therefore extended by duplicating their own sequence." Does this strategy create new sub sequences from synthetic fusion? Please, clarify.

Author’s response: Thank you for your comment. We clarified the point in the paper.

- Consider adding more explanation on why certain model choices (e.g., LSTM for DNA, ProteinBERT for protein sequences) were made.

Author’s response: Thank you for your comment. We added a section to the paper accordingly.

- Consider adding more detailed discussion on why HVSeeker outperforms other methods, particularly in terms of Recall and F1-score, would enhance the impact of these findings.

Author’s response: We thank the reviewer for the comment. We did add a paragraph to the paper explaining the performance of HVSeeker.

- As a general comment, consider adding deeper analysis of potential limitations or challenges faced during the study, such as the impact of data imbalance or the choice of model architectures.

Author’s response: We thank the reviewer for their insight and added comments to the paper where appropriate.

- While the paper mentions the availability of code on GitHub, it lacks detailed implementation details, such as the specific version of libraries used, the computing resources required, and a comprehensive list of dependencies. This information is crucial for the reproducibility of the results.

Author’s response: We thank the reviewer for his comment. The environments used for using HVSeeker are detailed in the corresponding environment.yml files. Additionally we now added a docker container to GitHub to make using HVSeeker easier. For training we use a single NVIDIA Tesla T4, 16GB GPU and now indicated so in the Readme.

Minor points:

- I recommend formatting the equations within the text to ensure they are presented consistently and seamlessly. By incorporating equations as part of the text flow, the readability and coherence of the paper can be significantly improved. This approach helps to integrate mathematical expressions more naturally into the narrative and makes it easier for readers to follow the argument without having to shift focus between different types of content. An example of how to use equations within the text can be found here: https://i.stack.imgur.com/l4p15.png

Author’s response: We thank the reviewer for his comment and have integrated the suggested changes in

the paper.

- Consider to create a Release of the repository code.

Author’s response: We added a release to the current repository code.

- Missing close ")" in "(for a discussion, see [8].".

Author’s response: Thank you for the comment. We fixed the error.

- Other classification tools based on similarity are Kraken2 (https://doi.org/10.1186/s13059-019-1891-0), Centrifuge (https://doi.org/10.1101/gr.210641.116), FALCON-meta (https://doi.org/10.3390/genes9090445), etc.

Author’s response: Thank you for the comment. We completed the listing accordingly.

Reviewer #2:
In this manuscript, the authors developed a new tool, HVSeeker, that allows users to predict phage sequences from metagenomic sequence data using deep-learning-based methods. The results showed outperformance compared to the existing tools. As the reviewers introduced, the accurate prediction of phage genomes from metagenomic data is a crucial but still challenging task, and thus good bioinformatic tools have been demanded. The deep-learning-based approach is a good way, especially for discovering novel phage genomes. Although I am not a specialist in machine learning, I feel the proposed tool is well-developed and may be useful in wide microbiology fields. I found writing problems and scientific issues that made it difficult to understand the study correctly. Also, some educational descriptions might be unnecessary as a scientific article. Those should be addressed before publication. I have some suggestions that would increase the clarity and impact of this
manuscript if addressed.

Major:

- Results. "Optimizing Sequence Length for Improved Prediction Performance" section. If my understanding is correct, the input data is fixed to be 1000 bp-length windows, and this part concludes that the model trained on the same 1000 bp window showed the best performance. It seems natural. Why did the authors do these tests? The sentence "This method has shown promising results on sequences with various lengths, ranging from 200 to 1500 base pairs." in the abstract means the sequence windows are used for training, and not for input, right? If so, the sentence is misleading. The proposed tool was designed for 1000 bp sequence windows as an input for phage sequence prediction. Metagenomic reads are typically short, such as 150 bp (Illumina HiSeq single-end), and up to about 550 bp (2x300 bp paired-end read after merge with 50 bp overlap) which might not be appropriate for HVSeeker. On the other hand, metagenomic assembled contigs easily reach over 1000 bp. What is the target data of the tool the authors assume (contigs, near-complete phage genome, or else)? The main targeted subjects and potential limitations of this tool would be better discussed in the manuscript.

Author’s response: We thank the reviewer for the comment. The model automatically preprocesses the input to facilitate the use of assemblies of different sizes. While the final model creates samples of a fixed size of 1000 for the input, we previously created models for different sizes to find out which will generalize best. We did clarify this now more in the paper.

- The term "Bacteria" is used overall in the manuscript. Can HVSeeker used for archaeal sequences? If so,

why were archaeal genomes and archaeophages not tested? Or, can the term Bacteria be rephrased as Prokaryotes? Can the tool detect viruses with large genomes (e.g., Megavirus), virophages, and eukaryotic viruses? Were only double-stranded DNA (dsDNA) viruses analyzed or were other types (single-strand DNA and RNA) viruses included? What is the difference between "Virus" and "Phage" in this manuscript?

Author’s response: HVSeeker is trained on bacterial genomes and bacteriophages, not on archaeal genomes and viruses that infect archaea. Bacterial and bacteriophage genome sequences are more available and more relevant to human health and industries. Archaeal genomes are less studied and often found in extreme environments, resulting in fewer reference genomes. However, HVSeeker's performance is acceptable for archaeal sequences. Therefore, in the future, we plan to train HVSeeker on archaeal sequences and viruses infecting archaea.

HVSeeker is capable of detecting viruses with large genomes, such as Megavirus, and virophages.

In the paper, the term "Virus" is used as a general term, while "Phage" is used to be more commonly used for bacteriophages.

- What substantial differences between HVseeker and other existing tools make the higher performance of the proposed tool? Is this related to the algorithm selection, data processing, or something else? This point should be clarified in the abstract and at the end of the Introduction.

Author’s response: Thank you for your comment. We integrated the suggested changes into the paper.

- Introduction. The sentences between "For that reason, other methods have been proposed." and "~ and phylogenetic analysis have been employed (for a discussion, see [8]." can be deleted. Assembly and binning are not related to the manuscript.

Author’s response: Thank you for the comment. We changed the manuscript accordingly.

- Methods. "The full dataset comprises 536 and 2687 bacterial and phage sequences, respectively.". Are there any potential biases, such as phylogeny, GC content, genome size, source environment, etc? Is the number of genomes sufficient for performance evaluation? The content and quality of the dataset are critical for following training and performance testing. Please provide more detailed information and discuss potential limitations.

Author’s response: To assess the quality of the dataset and the limits of the model, we test it comprehensively using different levels of test homologies. The details of this process can be found in the supplementary.

Minor:

- Abstract. Two models are proposed in this manuscript: HVseeker-DNA and HVSeeker-protein. It would be better to describe the two terms in the abstract

Author’s response: Thank you for the comment. We included the suggested change.

- Methods. The sentence "In this section, we provide detailed information about the research design, data collection process, preprocessing steps, and the structure of the proposed ML models." may be needless.

Author’s response: Thank you for the comment. We did remove the sentence.

- Methods. The authors employ three distinct strategies. Are these used for other previous studies, or the author's original settings? I feel the sliding window is a common way while the other two are a bit tricky.

Why the windows with <1000 bp were not removed? The proportion of such windows should be small in most cases I think.

Author’s response: Thank you for the comment. For other tools, we maintained their original preprocessing steps. These methods are key contributions of HV-Seeker, and we wanted to evaluate their impact on the results. In fact, preprocessing is one reason HV-Seeker outperformed the other approaches. We did not remove windows with a size < 1000 bp to not bias evaluation measures to our favor.

- Methods. The section "These steps encapsulate the preprocessing stage of our research". It is a bit hard to understand the items for which or both of HVSeeker-DNA and HVSeeker-Protein. It would be better to restructure this section to improve readability.

Author’s response: Thank you for the suggestion. We modified the sentence accordingly.

- Methods. The "Evaluation Criteria" section is too educational descriptions for a scientific article. This part can be shortened.

Author’s response: Thank you for your comment. We have shortened the description where appropriate and restructured the section to align with the feedback from another reviewer.

- Results. The sentence "benchmark datasets collected from [26]" is too rough writing. It would be better to provide details about the dataset for readers not to read the cited paper to understand this examination at least.

Author’s response: Thank you for the comment. We updated the results section to include more detailed information about the dataset.

- Results. The discussion "Conversely, Seeker, with an F1 Score of 0.578, DeepVirFinder with an F1 score of 0.417, and PPR-Meta with 44, appears to require additional training data to enhance its performance.". The discussion seems baseless. Why did the authors feel that further training data would improve the scores of those tools rather than other factors?

Author’s response: Thank you for your comment. During training, the model’s accuracy improved initially but then stopped after several epochs, suggesting it couldn’t extract further information from the existing data. This either indicates a need for more data or an insufficiently complex model architecture. We now clarified this point in the paper.

- Figure 1-3. Those can be merged into one large figure with three subfigures.

Author’s response: We merged the corresponding figures.

- Figure 5. What X-axis indicate? The words inside the figure are a bit difficult to read.

Author’s response: We recreated the corresponding plots to make them easier to read.

- Tables. Unify the significant digits and align the digits in each column. I feel the best scores in each column would be better to be bolded for easy understanding of which tool provided the best scores.

Author’s response: As suggested, we improved the tables.

Editorial Manager®

Cookie Preference Center

We use cookies which are necessary to make our site work. We may also use additional cookies to analyze, improve and personalize our content and your digital experience. For more information, see our Cookie Policy.

You may choose not to allow some types of cookies. However, blocking some types may impact your experience of our site and the services we are able to offer. See the different category headings below to find out more or change your settings.

Allow all

Manage Consent Preferences

Strictly Necessary Cookies

Always active

These cookies are necessary for the website to function and cannot be switched off in our systems. They are usually only set in response to actions made by you which amount to a request for services, such as setting your privacy preferences, logging in or filling in forms. You can set your browser to block or alert you about these cookies, but some parts of the site will not then work. These cookies do not store any personally identifiable information.

Functional Cookies

These cookies enable the website to provide enhanced functionality and personalisation. They may be set by us or by third party providers whose services we have added to our pages. If you do not allow these cookies then some or all of these services may not function properly.

Performance Cookies

These cookies allow us to count visits and traffic sources so we can measure and improve the performance of our site. They help us to know which pages are the most and least popular and see how visitors move around the site.

Targeting Cookies

These cookies may be set through our site by our advertising partners. They may be used by those companies to build a profile of your interests and show you relevant adverts on other sites. If you do not allow these cookies, you will experience less targeted advertising.

Cookie List

Clear

Apply Cancel
Consent Leg.Interest

Confirm my choices

Powered by onetrus
