# Supplementary material for: HVSeeker: a deep-learning-based method for identification of host and viral DNA sequences
Source: Gigascience. 2025 May 15;14:giaf037. doi: 10.1093/gigascience/giaf037 (PMC12080225; doi:10.1093/gigascience/giaf037)
Supplement: giaf037_Authors_Response_To_Reviewer_Comments_Revision_2 [file giaf037_authors_response_to_reviewer_comments_revision_2.pdf]

Author's Response To Reviewer Comments

Dear Dr. Hans
Editor-in-Chief of GiGascience

Herewith we submit the revised version of our manuscript "HVSeeker: A Deep Learning-based Method for Identification of Host and Viral DNA sequences".

We have answered each point and have modified our submission accordingly. We have also carefully considered the reviewer's comments and integrated them into a thoroughly revised manuscript version.

We thank the reviewers for their supportive comments and constructive feedback and the editor for the opportunity to submit a revised manuscript. Our response to each comment below is in blue.

I am looking forward to hearing from you.

Sincerely yours,

Editorial Manager®

Cookie Preference Center

We use cookies which are necessary to make our site work. We may also use additional cookies to analyze, improve and personalize our content and your digital experience. For more information, see our Cookie Policy.

You may choose not to allow some types of cookies. However, blocking some types may impact your experience of our site and the services we are able to offer. See the different category headings below to find out more or change your settings.

Allow all

Manage Consent Preferences

Strictly Necessary Cookies

Always active

These cookies are necessary for the website to function and cannot be switched off in our systems. They are usually only set in response to actions made by you which amount to a request for services, such as setting your privacy preferences, logging in or filling in forms. You can set your browser to block or alert you about these cookies, but some parts of the site will not then work. These cookies do not store any personally identifiable information.

Functional Cookies

These cookies enable the website to provide enhanced functionality and personalisation. They may be set by us or by third party providers whose services we have added to our pages. If you do not allow these cookies then some or all of these services may not function properly.

Performance Cookies

These cookies allow us to count visits and traffic sources so we can measure and improve the performance of our site. They help us to know which pages are the most and least popular and see how visitors move around the site.

Targeting Cookies

These cookies may be set through our site by our advertising partners. They may be used by those companies to build a profile of your interests and show you relevant adverts on other sites. If you do not allow these cookies, you will experience less targeted advertising.

Cookie List

Clear

Apply Cancel

Consent Leg.Interest

Confirm my choices

Powered by onetrust
