# Supplementary material for: HVSeeker: a deep-learning-based method for identification of host and viral DNA sequences
Source: Gigascience. 2025 May 15;14:giaf037. doi: 10.1093/gigascience/giaf037 (PMC12080225; doi:10.1093/gigascience/giaf037)
Supplement: giaf037_Reviewer_1_Report_Original_Submission — Diogo Pratas -- 8/1/2024 [file giaf037_reviewer_1_report_original_submission.pdf]

Reviewer Report

Title: HVSeeker: A Deep Learning-based Method for Identification of Host and Viral DNA sequences

Version: Original Submission **Date:** 8/1/2024

Reviewer name: Diogo Pratas

Reviewer Comments to Author:

This article presents a well-structured and comprehensive study on HVSeeker, a novel deep learning method for classifying bacterial and phage genomes (for DNA and protein sequences). The work is timely and relevant, offering a valuable contribution to the field. However, there are some points that require attention.Major points:Prophages are bacterial viruses that have integrated their DNA into the genome of a host bacterium. The article does not discuss the impact of prophages in the classification, specially in the metagenomics context. Since prophages are relevant to the research context, it would be beneficial to at least do one of these two: a) include prophage analysis: integrate an analysis of prophages in the study to ensure comprehensive genomic and phenotypic insights; b) acknowledge prophage influence: discuss how prophages might influence the results and interpretations of the study, particularly if they are known to play a significant role in the bacterial strains under investigation.The following sentence is confusing: "the functions of genes, specifically the proteins they encode [9]. To determine whether an organism belongs to a species characterized by a genome, sequencing methods can be used. Earlier sequencing methods were based on gene sim-ilarity. In other words, it is required for a provided genome to be similar to the known virus genome to classify it as an organismof that virus.". It seems that sequencing and reconstruction/classification is somewhat mixed. Please, clarify.The paper does not provide sufficient details about the dataset's diversity, such as the sources and environments of the samples. Additionally, please clarify what removal of duplicate sequences mean (exact duplications?).Given the performance reported, especially on validation datasets, there might be concerns about overfitting. The paper poorly mention techniques used to prevent overfitting, such as data augmentation or early stopping criteria.The 2000bp provided a f1-score of 0. Please better explain this: "Figure 5 shows that the model trained on 2000 bp sequences consistently predicted the bacteria class, indicating potential overfitting and an inability to generalize".Please, clarify: "Rnn-VirSeeker appeared to struggle with underfitting, showing a preference for the Phage class".About the implementation: I was uable to test HVSeeker. To use HVSeeker many installations are required. Please, provide a container for proper testing.Moderate points:The following sentence: "An example of tools that are built on the binning method is MetaWatt [12] and CONCOT [13]." is in some way repeated (this information was provided before)."When padding sequences that are naturally shorter than the maximum number of base pairs, they are therefore extended by duplicating their own sequence." Does this strategy create new sub sequences from synthetic fusion? Please, clarify.Consider adding more explanation on why certain model choices (e.g., LSTM for DNA, ProteinBERT for protein sequences) were made.Consider adding more detailed discussion on why HVSeeker outperforms other methods, particularly in terms of Recall and F1-score, would enhance the impact of these findings.As a general comment, consider adding deeper analysis of potential limitations or challenges faced during the study,

such as the impact of data imbalance or the choice of model architectures.While the paper mentions the availability of code on GitHub, it lacks detailed implementation details, such as the specific version of libraries used, the computing resources required, and a comprehensive list of dependencies. This information is crucial for the reproducibility of the results.Minor points:I recommend formatting the equations within the text to ensure they are presented consistently and seamlessly. By incorporating equations as part of the text flow, the readability and coherence of the paper can be significantly improved. This approach helps to integrate mathematical expressions more naturally into the narrative and makes it easier for readers to follow the argument without having to shift focus between different types of content. An example of how to use equations within the text can be found here: https://i.stack.imgur.com/l4p15.pngConsider to create a Release of the repository code.Missing close ")" in "(for a discussion, see [8].".Other classification tools based on similarity are Kraken2 (https://doi.org/10.1186/s13059-019-1891-0), Centrifuge (https://doi.org/10.1101/gr.210641.116), FALCON-meta (https://doi.org/10.3390/genes9090445), etc.

Level of Interest

Please indicate how interesting you found the manuscript: Choose an item.

Quality of Written English

Please indicate the quality of language in the manuscript: Choose an item.

Declaration of Competing Interests

Please complete a declaration of competing interests, considering the following questions:

- Have you in the past five years received reimbursements, fees, funding, or salary from an organisation that may in any way gain or lose financially from the publication of this manuscript, either now or in the future?
- Do you hold any stocks or shares in an organisation that may in any way gain or lose financially from the publication of this manuscript, either now or in the future?
- Do you hold or are you currently applying for any patents relating to the content of the manuscript?
- Have you received reimbursements, fees, funding, or salary from an organization that holds or has applied for patents relating to the content of the manuscript?
- Do you have any other financial competing interests?
- Do you have any non-financial competing interests in relation to this paper?

If you can answer no to all of the above, write 'I declare that I have no competing interests' below. If your reply is yes to any, please give details below.

I declare that I have no competing interests

I agree to the open peer review policy of the journal. I understand that my name will be included on my report to the authors and, if the manuscript is accepted for publication, my named report including any attachments I upload will be posted on the website along with the authors' responses. I agree for my report to be made available under an Open Access Creative Commons CC-BY license (http://creativecommons.org/licenses/by/4.0/). I understand that any comments which I do not wish to be included in my named report can be included as confidential comments to the editors, which will not be published.

Choose an item.

To further support our reviewers, we have joined with Publons, where you can gain additional credit to further highlight your hard work (see: https://publons.com/journal/530/gigascience). On publication of this paper, your review will be automatically added to Publons, you can then choose whether or not to claim your Publons credit. I understand this statement.

Yes Choose an item.
