# Supplementary material for: HVSeeker: a deep-learning-based method for identification of host and viral DNA sequences
Source: Gigascience. 2025 May 15;14:giaf037. doi: 10.1093/gigascience/giaf037 (PMC12080225; doi:10.1093/gigascience/giaf037)
Supplement: giaf037_Reviewer_2_Report_Original_Submission — Satoshi Hiraoka -- 8/18/2024 [file giaf037_reviewer_2_report_original_submission.pdf]

Reviewer Report

Title: HVSeeker: A Deep Learning-based Method for Identification of Host and Viral DNA sequences

Version: Original Submission **Date:** 8/18/2024

Reviewer name: Satoshi Hiraoka

Reviewer Comments to Author:

In this manuscript, the authors developed a new tool, HVSeeker, that allows users to predict phage sequences from metagenomic sequence data using deep-learning-based methods. The results showed outperformance compared to the existing tools. As the reviewers introduced, the accurate prediction of phage genomes from metagenomic data is a crucial but still challenging task, and thus good bioinformatic tools have been demanded. The deep-learning-based approach is a good way, especially for discovering novel phage genomes. Although I am not a specialist in machine learning, I feel the proposed tool is well-developed and may be useful in wide microbiology fields. I found writing problems and scientific issues that made it difficult to understand the study correctly. Also, some educational descriptions might be unnecessary as a scientific article. Those should be addressed before publication. I have some suggestions that would increase the clarity and impact of this manuscript if addressed.Major:Results. "Optimizing Sequence Length for Improved Prediction Performance" section. If my understanding is correct, the input data is fixed to be 1000 bp-length windows, and this part concludes that the model trained on the same 1000 bp window showed the best performance. It seems natural. Why did the authors do these tests? The sentence "This method has shown promising results on sequences with various lengths, ranging from 200 to 1500 base pairs." in the abstract means the sequence windows are used for training, and not for input, right? If so, the sentence is misleading. The proposed tool was designed for 1000 bp sequence windows as an input for phage sequence prediction. Metagenomic reads are typically short, such as 150 bp (Illumina HiSeq single-end), and up to about 550 bp (2x300 bp paired-end read after merge with 50 bp overlap) which might not be appropriate for HVSeeker. On the other hand, metagenomic assembled contigs easily reach over 1000 bp. What is the target data of the tool the authors assume (contigs, near-complete phage genome, or else)? The main targeted subjects and potential limitations of this tool would be better discussed in the manuscript.The term "Bacteria" is used overall in the manuscript. Can HVSeeker used for archaeal sequences? If so, why were archaeal genomes and archaeophages not tested? Or, can the term Bacteria be rephrased as Prokaryotes? Can the tool detect viruses with large genomes (e.g., Megavirus), virophages, and eukaryotic viruses? Were only double-stranded DNA (dsDNA) viruses analyzed or were other types (single-strand DNA and RNA) viruses included? What is the difference between "Virus" and "Phage" in this manuscript?What substantial differences between HVseeker and other existing tools make the higher performance of the proposed tool? Is this related to the algorithm selection, data processing, or something else? This point should be clarified in the abstract and at the end of the Introduction.Introduction. The sentences between "For that reason, other methods have been proposed." and "~ and phylogenetic analysis have been employed (for a discussion, see [8]." can be deleted. Assembly and binning are not related to the manuscript.Methods. "The full dataset comprises

536 and 2687 bacterial and phage sequences, respectively.". Are there any potential biases, such as phylogeny, GC content, genome size, source environment, etc? Is the number of genomes sufficient for performance evaluation? The content and quality of the dataset are critical for following training and performance testing. Please provide more detailed information and discuss potential limitations.Minor:Abstract. Two models are proposed in this manuscript: HVseeker-DNA and HVSeeker-protein. It would be better to describe the two terms in the abstract.Methods. The sentence "In this section, we provide detailed information about the researchdesign, data collection process, preprocessing steps, and the structure of the proposed ML models." may be needless.Methods. The authors employ three distinct strategies. Are these used for other previous studies, or the author's original settings? I feel the sliding window is a common way while the other two are a bit tricky. Why the windows with <1000 bp were not removed? The proportion of such windows should be small in most cases I think.Methods. The section "These steps encapsulate the preprocessing stage of our research". It is a bit hard to understand the items for which or both of HVSeeker-DNA and HVSeeker-Protein. It would be better to restructure this section to improve readability.Methods. The "Evaluation Criteria" section is too educational descriptions for a scientific article. This part can be shortened.Results. The sentence "benchmark datasets collected from [26]" is too rough writing. It would be better to provide details about the dataset for readers not to read the cited paper to understand this examination at least.Results. The discussion "Conversely, Seeker, with an F1 Score of 0.578, DeepVirFinder with an F1 score of 0.417, and PPR-Meta with 44, appears to require additional training data to enhance its performance.". The discussion seems baseless. Why did the authors feel that further training data would improve the scores of those tools rather than other factors?Figure 1-3. Those can be merged into one large figure with three subfigures.Figure 5. No error bars? A statistical test can be done in this analysis.Figure 6. What X-axis indicate? The words inside the figure are a bit difficult to read.Tables. Unify the significant digits and align the digits in each column. I feel the best scores in each column would be better to be bolded for easy understanding of which tool provided the best scores.

Level of Interest

Please indicate how interesting you found the manuscript: Choose an item.

Quality of Written English

Please indicate the quality of language in the manuscript: Choose an item.

Declaration of Competing Interests

Please complete a declaration of competing interests, considering the following questions:

- Have you in the past five years received reimbursements, fees, funding, or salary from an organisation that may in any way gain or lose financially from the publication of this manuscript, either now or in the future?

- Do you hold any stocks or shares in an organisation that may in any way gain or lose financially from the publication of this manuscript, either now or in the future?
- Do you hold or are you currently applying for any patents relating to the content of the manuscript?
- Have you received reimbursements, fees, funding, or salary from an organization that holds or has applied for patents relating to the content of the manuscript?
- Do you have any other financial competing interests?
- Do you have any non-financial competing interests in relation to this paper?

If you can answer no to all of the above, write 'I declare that I have no competing interests' below. If your reply is yes to any, please give details below.

I declare that I have no competing interests

I agree to the open peer review policy of the journal. I understand that my name will be included on my report to the authors and, if the manuscript is accepted for publication, my named report including any attachments I upload will be posted on the website along with the authors' responses. I agree for my report to be made available under an Open Access Creative Commons CC-BY license (http://creativecommons.org/licenses/by/4.0/). I understand that any comments which I do not wish to be included in my named report can be included as confidential comments to the editors, which will not be published.

Choose an item.

To further support our reviewers, we have joined with Publons, where you can gain additional credit to further highlight your hard work (see: https://publons.com/journal/530/gigascience). On publication of this paper, your review will be automatically added to Publons, you can then choose whether or not to claim your Publons credit. I understand this statement.

Yes Choose an item.
